# Supplementary figures and images for: Standardized effect sizes are far from “Standardized”: A primer and empirical illustration in depression psychotherapy meta-analyses
Source: PLOS Ment Health. 2025 Jul 1;2(7):e0000347. doi: 10.1371/journal.pmen.0000347 (PMC12798590; doi:10.1371/journal.pmen.0000347)

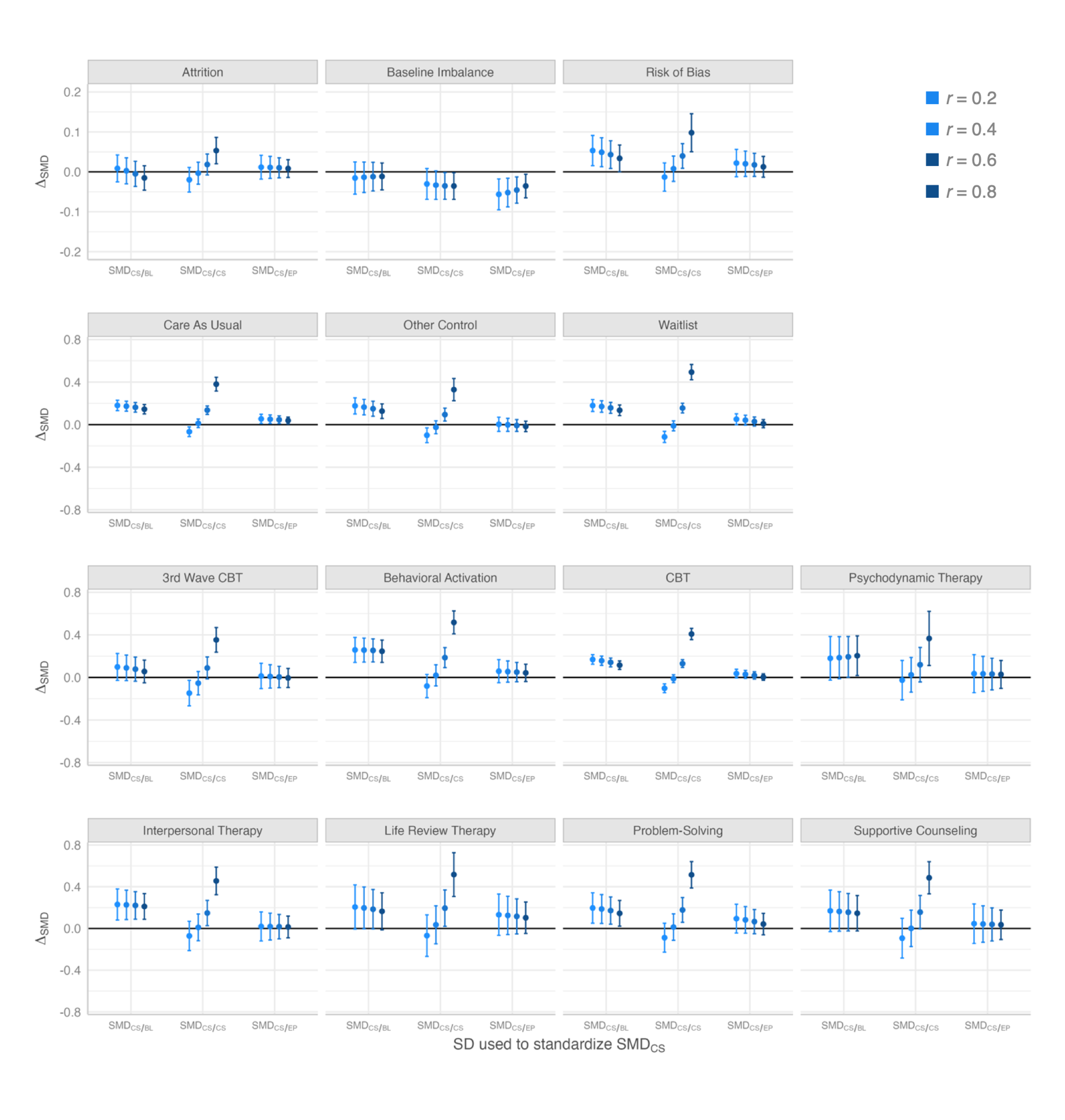

Supplement: S1 Fig — ΔSMD = Difference between the pooled effect based on endpoint SMDs (SMDEP/EP), and SMDs calculated using change scores (SMDCS). “Attrition” refers to the proportion of participants who were lost to follow-up, pooled across both trial arms (continuous covariate); “Baseline Imbalance” to the absolute value of the between-group SMD at baseline (continuous covariate); and “Risk of Bias” to the number of domains assessed to have a low risk of bias (continuous covariate; 0–4). (TIFF) [file pmen.0000347.s001.tiff]
