# Supplementary material for: Standardized effect sizes are far from “Standardized”: A primer and empirical illustration in depression psychotherapy meta-analyses
Source: PLOS Ment Health. 2025 Jul 1;2(7):e0000347. doi: 10.1371/journal.pmen.0000347 (PMC12798590; doi:10.1371/journal.pmen.0000347)
Supplement: S1 Text — No legend. (PDF) [file pmen.0000347.s002.pdf]

S1. *True Effect and Estimated SMD Conditional on Calculation Methods (Simulated Example).*

To demonstrate the behavior of different SMD calculation methods when the true treatment effect (θ) is known, we conducted a simulation using parameter values representative of psychological intervention trials. Following McKenzie et al. (2016)[1], we assumed that in a randomized trial i, control group pre- and post-test scores X_{C_i} and Y_{C_i} are draws from a bivariate normal distribution:

$$\begin{pmatrix} X_{C_i} \\ Y_{C_i} \end{pmatrix} \sim \mathrm{BVN}\left(\begin{pmatrix} 0 \\ \alpha \end{pmatrix}, \begin{pmatrix} 1 & \rho \\ \rho & 1 \end{pmatrix}\right) \tag{1}$$

with α representing the mean change over time, and ρ the pre-post-test correlation. In the intervention group, pre- and post-test scores are assumed to be drawn from the following bivariate normal distribution:

$$\begin{pmatrix} X_{T_i} \\ Y_{T_i} \end{pmatrix} \sim \mathrm{BVN}\left(\begin{pmatrix} 0 \\ \theta + \alpha + u_i \end{pmatrix}, \begin{pmatrix} 1 & \rho\sigma_{Y_T} \\ \rho\sigma & \sigma^2_{Y_T} \end{pmatrix}\right) \tag{2}$$

where θ is the true treatment effect, u_i is a study-specific random effect, and $\sigma^2_{Y_T}$ is the variance of post-test scores in the treatment group, assumed to be 1. Random effects u_i follow a normal distribution with mean zero and between-study heterogeneity variance τ^2. For each trial i, sample sizes were drawn from a Weibull distribution, ensuring that $n_C = n_T \geq 5$. Shape and scale parameters were determined by fitting a Weibull to the empirical distribution of sample sizes in the "Depression: Psychotherapy vs. Control" dataset used for the present study. Parameters for our exemplary simulation were thus set to the following values:

$\theta = 0.8$	Large effect of therapy
$\tau = 0.3$	Moderate-to-large heterogeneity
$\rho = 0.4$	Derived from empirical estimates[2]
n_C, n_T~Weibull$(k = 1.12, \lambda = 44)$	Derived from database.

The value of the "true" SMD δ was set to $\delta = \theta/\sigma = 0.8$. Following Hedges et al.[3], we define the true effect size as:

$$\delta = \frac{\mu_T - \mu_C}{\sigma} \tag{3}$$

whereby σ is explicitly defined as the variability of endpoint scores Y; no matter if an estimate of $\mu_T - \mu_C$ is obtained from unadjusted endpoint scores, change scores, or adjusted means in practice (cf. p. 266). Since post-test scores σ^2_Y are constrained to 1 in our simulation, (3) reduces to $\mu_T - \mu_C = \theta = 0.8$.

For our simulation, we assumed that pre-post correlations r are not reported, and therefore have to be imputed, choosing from $r \in \{0.2, 0.4, 0.6, 0.8\}$. For each of these scenarios, data of 10,000 studies were simulated, and we calculated $\mathrm{SMD}_{\mathrm{EP/EP}}$, $\mathrm{SMD}_{\mathrm{CS/BL}}$, $\mathrm{SMD}_{\mathrm{CS/CS}}$, $\mathrm{SMD}_{\mathrm{CS/EP}}$ (see "Calculation of SMDs" section in the manuscript for definitions). For each of these SMD variants, we also estimated the overall true effect $\hat{\mu}$ across all studies, employing the DerSimonian-Laird estimator. Results of the simulation are shown in the table below:

	Standardizer ($\mathrm{SMD}_{\mathrm{CS}}$)	$\mathrm{SMD}_{\mathrm{EP}}$				$\mathrm{SMD}_{\mathrm{CS}}$				$\Delta_{\mathrm{EP\text{-}CS}}$
		$\hat{\mu}$	Δ_μ	S.E.	$\hat{\tau}$	$\hat{\mu}$	Δ_μ	S.E.	$\hat{\tau}$	
r=0.2	Baseline SD	0.787	-0.013	0.004	0.293	0.789	-0.011	0.004	0.258	-0.002

[1] McKenzie JE, Herbison GP, Deeks JJ. Impact of analysing continuous outcomes using final values, change scores and analysis of covariance on the performance of meta-analytic methods: a simulation study. Research synthesis methods Wiley Online Library; 2016;7(4):371–386.
[2] Balk EM, Earley A, Patel K, Trikalinos TA, Dahabreh IJ. Empirical Assessment of Within-Arm Correlation Imputation in Trials of Continuous Outcome. 2012; PMID:23326900 (see main paper, discussion section for further details).
[3] Hedges LV, Tipton E, Zejnullahi R, Diaz KG. Effect sizes in ANCOVA and difference-in-differences designs. *Br J Math Stat Psychol.* 2023;76(2):259-282. doi:10.1111/bmsp.12296.

	Standardizer (SMD_{CS})	SMD_{EP} $\hat{\mu}$	Δ_μ	S.E.	$\hat{\tau}$	SMD_{CS} $\hat{\mu}$	Δ_μ	S.E.	$\hat{\tau}$	$\Delta_{EP\text{-}CS}$
	Change SD	0.787	-0.013	0.004	0.293	0.624	-0.176	0.003	0.126	0.163
	Endpoint SD	0.787	-0.013	0.004	0.293	0.790	-0.010	0.004	0.269	-0.003
r=0.4	Baseline SD	0.784	-0.016	0.004	0.289	0.787	-0.013	0.004	0.285	-0.003
	Change SD	0.784	-0.016	0.004	0.289	0.713	-0.087	0.004	0.244	0.071
	Endpoint SD	0.784	-0.016	0.004	0.289	0.787	-0.013	0.004	0.295	-0.003
r=0.6	Baseline SD	0.783	-0.017	0.004	0.292	0.779	-0.021	0.004	0.314	0.004
	Change SD	0.783	-0.017	0.004	0.292	0.855	0.055	0.004	0.360	-0.072
	Endpoint SD	0.783	-0.017	0.004	0.292	0.780	-0.020	0.004	0.320	0.003
r=0.8	Baseline SD	0.787	-0.013	0.004	0.289	0.779	-0.021	0.004	0.341	0.008
	Change SD	0.787	-0.013	0.004	0.289	1.177	0.377	0.006	0.545	-0.390
	Endpoint SD	0.787	-0.013	0.004	0.289	0.780	-0.020	0.004	0.345	0.007

Note. $\hat{\mu}$ = estimated pooled effect; Δ_μ = difference between estimated and true effect (θ=δ=0.8); $\hat{\tau}$ = estimated between-study heterogeneity; $\Delta_{EP\text{-}CS}$ = difference between endpoint and change score SMD.

Results of the simulations and effect size divergences are also displayed in the plots below. Green diamonds indicate the true effect underlying the simulation ($\theta = 0.8$); red crosses indicate the calculated SMD value.

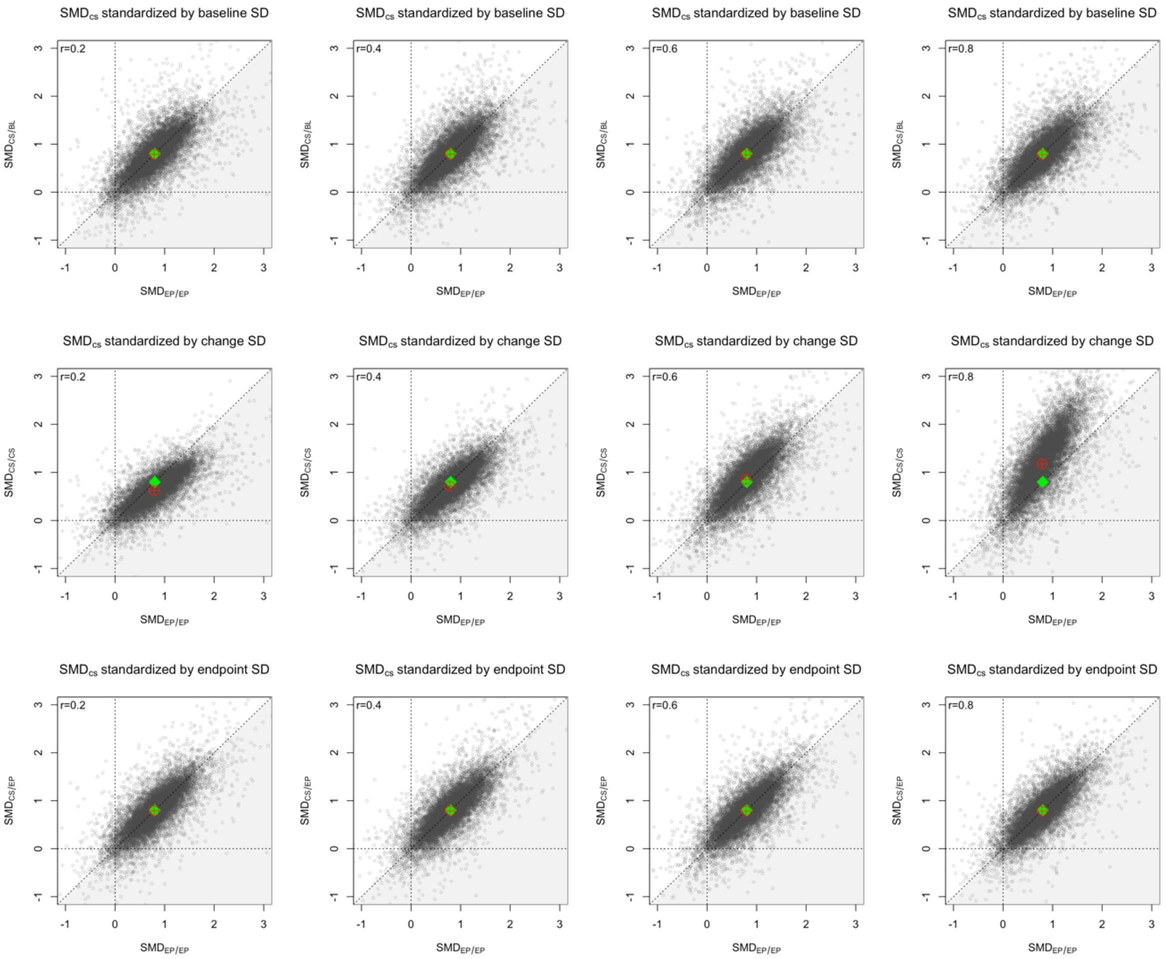

Overall, the simulated results above show that different SMDs variants can strongly and systematically diverge from each other. This behavior is most pronounced for $SMD_{CS/CS}$, which heavily depends on the (imputed) pre-post correlation r. Especially for very high or low r values, $SMD_{CS/CS}$ shows strong discrepancies to both the endpoint SMD and the true underlying effect θ in our simulation.

R code used to generate the simulations has been added to the online material repository of this study (doi.org/10.5281/zenodo.10694719).
