# Supplementary material for: Standardized effect sizes are far from “Standardized”: A primer and empirical illustration in depression psychotherapy meta-analyses
Source: PLOS Ment Health. 2025 Jul 1;2(7):e0000347. doi: 10.1371/journal.pmen.0000347 (PMC12798590; doi:10.1371/journal.pmen.0000347)
Supplement: S2 Text — No legend. (PDF) [file pmen.0000347.s003.pdf]

S6. *References of the Included Studies.*

Psychotherapy versus Control Groups

Aagaard, J., Foldager, L., Makki, A., Hansen, V., & M¸ller-Nielsen, K. (2017). The efficacy of psychoeducation on recurrent depression: a randomized trial with a 2-year follow-up. Nord J Psychiatry, 71(3), 223-229.

Abas M, Nyamayaro P, Bere T, et al. Feasibility and acceptability of a task-shifted intervention to enhance adherence to HIV medication and improve depression in people living with HIV in Zimbabwe, a low income country in sub-Saharan Africa. AIDS and Behavior 2018; 22(1): 86-101.

Abbas, Q., Latif, S., Ayaz Habib, H., Shahzad, S., Sarwar, U., Shahzadi, M., . . . Washdev, W. (2023). Cognitive behavior therapy for diabetes distress, depression, health anxiety, quality of life and treatment adherence among patients with Type-II diabetes mellitus: A randomized control trial. BMC Psychiatry, 23. doi:10.1186/s12888-023-04546-w

Afonso, R., & Bueno, B. (2009). Efectos de un programa de reminiscencia sobre la sintomatología depresiva en una muestra de población mayor portuguesa. Revista Española de Geriatría y Gerontología, 44(6), 317-322.

Ahmadpanah, M., Paghale, S. J., Bakhtyari, A., Kaikhavani, S., Aghaei, E., Nazaribadie, M., . . . Brand, S. (2016). Effects of psychotherapy in combination with pharmacotherapy, when compared to pharmacotherapy only on blood pressure, depression, and anxiety in female patients with hypertension.

Alhusen, J. L., Hayat, M. J., & Borg, L. (2021, Feb). A pilot study of a group-based perinatal depression intervention on reducing depressive symptoms and improving maternal-fetal attachment and maternal sensitivity. Arch Womens Ment Health, 24(1), 145-154. https://doi.org/10.1007/s00737-020-01032-0

Allart-Van Dam E, Hosman CMH, Hoogduin CAL, Schaap CPDR. The coping with depression course: Short-term outcomes and mediating effects of a randomized controlled trial in the treatment of subclinical depression. Behavior Therapy. 2003;34(3):381-96.

Amani, B., Merza, D., Savoy, C., Streiner, D., Bieling, P., Ferro, M. A., & Van Lieshout, R. J. (2021, Nov 9). Peer-Delivered Cognitive-Behavioral Therapy for Postpartum Depression: A Randomized Controlled Trial. J Clin Psychiatry, 83(1). https://doi.org/10.4088/JCP.21m13928

Amano, M., Katayama, N., Umeda, S., Terasawa, Y., Tabuchi, H., Kikuchi, T., . . . Nakagawa, A. (2023). The effect of cognitive behavioral therapy on future thinking in patients with major depressive disorder: A randomized controlled trial. Frontiers in Psychiatry, 14. doi:10.3389/fpsyt.2023.997154

Ammerman RT, Putnam FW, Altaye M, Stevens J, Teeters AR, Van Ginkel JB. A clinical trial of in-home CBT for depressed mothers in home visitation. Behavior Therapy. 2013;44(3):359-72.

Andersson G, Bergström J, Holländare F, Carlbring P, Kaldo V, Ekselius L. Internet-based self-help for depression: Randomised controlled trial. British Journal of Psychiatry. 2005;187(5):456-61.

Arean PA, Perri MG, Nezu AM, Schein RL, Christopher F, Joseph TX. Comparative effectiveness of social problem-solving therapy and reminiscence therapy as treatments for depression in older adults. Journal of Consulting and Clinical Psychology. 1993;61(6):1003-10.

Arjadi R, Nauta MH, Scholte WF, et al. Internet-based behavioural activation with lay counsellor support versus online minimal psychoeducation without support for treatment of depression: a randomised controlled trial in Indonesia. The lancet psychiatry 2018; 5(9): 707-16.

Au, A., Nan, H., Sum, R., Ng, F., Kwong, A., & Wong, S. (2022). Cognitive behavioural therapy for adherence and sub-clinical depression in type 2 diabetes: a randomised controlled trial (abridged secondary publication). Hong Kong Med J, 28 Suppl 3(3), 21-23.

Ayen I, Hautzinger M. Cognitive behavior therapy for depression in menopausal women. A controlled, randomized treatment study. Zeitschrift fur Klinische Psychologie und Psychotherapie. 2004;33(4):290-9.

Baker AL, Kavanagh DJ, Kay-Lambkin FJ, Hunt SA, Lewin TJ, Carr VJ, et al. Randomized controlled trial of cognitive-behavioural therapy for coexisting depression and alcohol problems: short-term outcome. Addiction (Abingdon, England). 2010;105(1):87-99.

Barnhofer T, Crane C, Hargus E, Amarasinghe M, Winder R, Williams JM. Mindfulness-based cognitive therapy as a treatment for chronic depression: A preliminary study. Behaviour Research and Therapy. 2009;47(5):366-73.

Basirat, Z., Kheirkhah, F., Faramarzi, M., Esmaelzadeh, S., Khafri, S., & Tajali, Z. (2022). Pharmacotherapy or Psychotherapy? Selective Treatment Depression in The Infertile Women with Recurrent Pregnancy Loss: A Triple-Arm Randomized Controlled Trial. International Journal of Fertility and Sterility, 16(3), 211-219. doi:10.22074/ijfs.2021.529258.1124

Baumeister, H., Paganini, S., Sander, L. B., Lin, J., Schlicker, S., Terhorst, Y., Moshagen, M., Bengel, J., Lehr, D., & Ebert, D. D. (2021). Effectiveness of a Guided Internet- and Mobile-Based Intervention for Patients with Chronic Back Pain and Depression (WARD-BP): A Multicenter, Pragmatic Randomized Controlled Trial. Psychother Psychosom, 90(4), 255-268. https://doi.org/10.1159/000511881

Baumgartner, C., Schaub, M. P., Wenger, A., Malischnig, D., Augsburger, M., Lehr, D., . . . Haug, S. (2021). "Take Care of You" - Efficacy of integrated, minimal-guidance, internet-based self-help for reducing co-occurring alcohol misuse and depression symptoms in adults: Results of a three-arm randomized controlled trial. Drug Alcohol Depend, 225, 108806. doi:10.1016/j.drugalcdep.2021.108806

Beach SR, O'Leary KD. Treating depression in the context of marital discord: Outcome and predictors of response of marital therapy versus cognitive therapy. Behavior Therapy. 1992;23(4):507-28.

Bedard M, Felteau M, Marshall S, Cullen N, Gibbons C, Dubois S, et al. Mindfulness-based cognitive therapy reduces symptoms of depression in people with a traumatic brain injury: Results from a randomized controlled trial. Journal of Head Trauma Rehabilitation. 2014;29(4):E13-E22.

Beeber LS, Holditch-Davis D, Perreira K, Schwartz TA, Lewis V, Blanchard H, et al. Short-term in-home intervention reduces depressive symptoms in Early Head Start Latina mothers of infants and toddlers. Research in Nursing and Health. 2010;33(1):60-76.

Bendig, E., Bauereiß, N., Buntrock, C., Habibović, M., Ebert, D. D., & Baumeister, H. (2021). Lessons learned from an attempted randomized-controlled feasibility trial on "WIDeCAD" - An internet-based depression treatment for people living with coronary artery disease (CAD) [Article]. Internet Interventions, 24. https://doi.org/10.1016/j.invent.2021.100375

Berger T, Hämmerli K, Gubser N, Andersson G, Caspar F. Internet-based treatment of depression: A randomized controlled trial comparing guided with unguided self-help. Cognitive Behaviour Therapy. 2011;40(4):251-66.

Berman, M. I., Park, J., Kragenbrink, M. E., & Hegel, M. T. (2022). Accept Yourself! A Pilot Randomized Controlled Trial of a Self-Acceptance-Based Treatment for Large-Bodied Women With Depression. Behav Ther, 53(5), 913-926. doi:10.1016/j.beth.2022.03.002

Bilich, L. L., Deane, F. P., Phipps, A. B., Barisic, M., & Gould, G. (2008). Effectiveness of bibliotherapy self-help for depression with varying levels of telephone helpline support. Clin Psychol Psychother, 15(2), 61-74. doi:10.1002/cpp.562

Boele FW, Klein M, Verdonck-de Leeuw IM, et al. Internet-based guided self-help for glioma patients with depressive symptoms: a randomized controlled trial. Journal of neuro-oncology 2018; 137(1): 191-203.

Boeschoten, R. E., Dekker, J., Uitdehaag, B. M. J., Beekman, A. T. F., Hoogendoorn, A. W., Collette, E. H., . . . Van Oppen, P. (2017). Internet-based treatment for depression in multiple sclerosis: A randomized controlled trial. Multiple Sclerosis, 23(8), 1112-1122.

Bohlmeijer ET, Fledderus M, Rokx TA, Pieterse ME. Efficacy of an early intervention based on acceptance and commitment therapy for adults with depressive symptomatology: Evaluation in a randomized controlled trial. Behaviour research and therapy. 2011;49(1):62-7.

Bolton P, Bass J, Neugebauer R, Verdeli H, Clougherty KF, Wickramaratne P, et al. Group interpersonal psychotherapy for depression in rural Uganda: A randomized controlled trial. JAMA. 2003;289(23):3117-24.

Bower, J. E., Partridge, A. H., Wolff, A. C., Thorner, E. D., Irwin, M. R., Joffe, H., Petersen, L., Crespi, C. M., & Ganz, P. A. (2021, Nov 1). Targeting Depressive Symptoms in Younger Breast Cancer Survivors: The Pathways to Wellness Randomized Controlled Trial of Mindfulness Meditation and Survivorship Education. J Clin Oncol, 39(31), 3473-3484. https://doi.org/10.1200/jco.21.00279

Bowman D, Scogin F, Lyrene B. The efficacy of self-examination therapy and cognitive bibliotherapy in the treatment of mild to moderate depression. Psychotherapy Research. 1995;5(2):131-40.

Braun, L., Titzler, I., Terhorst, Y., Freund, J., Thielecke, J., Ebert, D. D., & Baumeister, H. (2021, Jan 1). Effectiveness of guided internet-based interventions in the indicated prevention of depression in green professions (PROD-A): Results of a pragmatic randomized controlled trial. J Affect Disord, 278, 658-671. https://doi.org/10.1016/j.jad.2020.09.066

Brown RA, Lewinsohn PM. A psychoeducational approach to the treatment of depression: Comparison of group, individual, and minimal contact procedures. Journal of Consulting and Clinical Psychology. 1984;52(5):774.

Buhrman M, Syk M, Burvall O, Hartig T, Gordh T, Andersson G. Individualized Guided Internet-delivered Cognitive Behaviour Therapy for Chronic Pain Patients with Comorbid Depression and Anxiety: A Randomized Controlled Trial. Clinical Journal of Pain 2014.

Buntrock C, Ebert D, Lehr D, Riper H, Smit F, Cuijpers P, et al. Effectiveness of a web-based cognitive behavioural intervention for subthreshold depression: Pragmatic randomised controlled trial. Psychotherapy and Psychosomatics. 2015;84(6):348-58.

Burns A, O'Mahen H, Baxter H, Bennert K, Wiles N, Ramchandani P, et al. A pilot randomised controlled trial of cognitive behavioural therapy for antenatal depression. BMC psychiatry. 2013;13:33.

Carlbring P, Hagglund M, Luthstrom A, Dahlin M, Kadowaki A, Vernmark K, et al. Internet-based behavioral activation and acceptance-based treatment for depression: A randomized controlled trial. Journal of Affective Disorders. 2013;148(2-3):331-7.

Carr, A., Finnegan, L., Griffin, E., Cotter, P., & Hyland, A. (2017). A Randomized Controlled Trial of the Say Yes to Life (SYTL) Positive Psychology Group Psychotherapy Program for Depression: An Interim Report. Journal of Contemporary Psychotherapy, 47(3), 153-161.

Carta MG, Petretto D, Adamo S, Bhat KM, Lecca ME, Mura G, et al. Counseling in primary care improves depression and quality of life. Clinical Practice and Epidemiology in Mental Health. 2012;8.

Casanas R, Catalan R, del Val JL, Real J, Valero S, Casas M. Effectiveness of a psycho-educational group program for major depression in primary care: A randomized controlled trial. BMC psychiatry. 2012;12:230.

Castonguay LG, Schut AJ, Aikens DE, Constantino MJ, Laurenceau J-P, Bologh L, et al. Integrative cognitive therapy for depression: A preliminary investigation. Journal of Psychotherapy Integration. 2004;14(1):4-20.

Chan AS, Wong QY, Sze SL, Kwong PP, Han YM, Cheung MC. A Chinese Chan-based mind-body intervention for patients with depression. Journal of Affective Disorders. 2012;142(1-3):283-9.

Chan MF, Ng SE, Tien A, Man Ho RC, Thayala J. A randomised controlled study to explore the effect of life story review on depression in older Chinese in Singapore. Health and Social Care in the Community. 2013;21(5):545-53.

Chesney MA, Chambers DB, Taylor JM, Johnson LM, Folkman S. Coping effectiveness training for men living with HIV: Results from a randomized clinical trial testing a group-based intervention. Psychosomatic Medicine. 2003;65(6):1038-46.

Chiang KJ, Chen TH, Hsieh HT, Tsai JC, Ou KL, Chou KR. One-year follow-up of the effectiveness of cognitive behavioral group therapy for patients' depression: A randomized, single-blinded, controlled study. Scientific World Journal. 2015;2015:Article ID 373149.

Cho HJ, Kwon JH, Lee JJ. Antenatal cognitive-behavioral therapy for prevention of postpartum depression: A pilot study. Yonsei medical journal. 2008;49(4):553-62.

Choi I, Zou J, Titov N, Dear BF, Li S, Johnston L, et al. Culturally attuned Internet treatment for depression amongst Chinese Australians: A randomised controlled trial. Journal of Affective Disorders. 2012;136(3):459-68.

Choi NG, Marti CN, Wilson NL, et al. Effect of Telehealth Treatment by Lay Counselors vs by Clinicians on Depressive Symptoms Among Older Adults Who Are Homebound: A Randomized Clinical Trial. JAMA Netw Open. 2020;3(8):e2015648.

Choy JC, Lou VW. Effectiveness of the modified instrumental reminiscence intervention on psychological well-being among community-dwelling chinese older adults: A randomized controlled trial. American Journal of Geriatric Psychiatry. 2016;24(1):60-9.

Clark R, Tluczek A, Brown R. A mother–infant therapy group model for postpartum depression. Infant Mental Health Journal. 2008;29(5):514-36.

Clark R, Tluczek A, Wenzel A. Psychotherapy for postpartum depression: A preliminary report. American Journal of Orthopsychiatry. 2003;73(4):441-54.

Cohen S, O'Leary KD, Foran H. A randomized clinical trial of a brief, problem-focused couple therapy for depression. Behavior Therapy. 2010;41(4):433-46.

Cramer H, Salisbury C, Conrad J, Eldred J, Araya R. Group cognitive behavioural therapy for women with depression: Pilot and feasibility study for a randomised controlled trial using mixed methods. BMC psychiatry. 2011;11:82.

Cuijpers, P., Heim, E., Abi Ramia, J., Burchert, S., Carswell, K., Cornelisz, I., . . . El Chammay, R. (2022). Effects of a WHO-guided digital health intervention for depression in Syrian refugees in Lebanon: A randomized controlled trial. PLoS Med, 19(6), e1004025. doi:10.1371/journal.pmed.1004025

Cuijpers, P., Heim, E., Ramia, J. A., Burchert, S., Carswell, K., Cornelisz, I., . . . El Chammay, R. (2022). Guided digital health intervention for depression in Lebanon: randomised trial. Evidence Based Mental Health, 25(e1), e34. doi:10.1136/ebmental-2021-300416

Davoudi M, Taheri AA, Foroughi AA, Ahmadi SM, Heshmati K. Effectiveness of acceptance and commitment therapy (ACT) on depression and sleep quality in painful diabetic neuropathy: a randomized clinical trial. Journal of Diabetes and Metabolic Disorders. 2020.

De Groot, M., Shubrook, J. H., Hornsby, W. G., Pillay, Y., Mather, K. J., Fitzpatrick, K., . . . Saha, C. (2019). Program ACTIVE II: Outcomes from a randomized, multistate community-based depression treatment for rural and urban adults with type 2 diabetes. Diabetes care, 42(7), 1185-1193. doi:10.2337/dc18-2400

De Jong M, Peeters F, Gard T, et al. A randomized controlled pilot study on mindfulness-based cognitive therapy for unipolar depression in patients with chronic pain. Journal of clinical psychiatry 2018; 79(1): 26-34.

Dekker RL, Moser DK, Peden AR, Lennie TA. Cognitive therapy improves three-month outcomes in hospitalized patients with heart failure. Journal of cardiac failure. 2012;18(1):10-20.

Demir, S., & Ercan, F. (2022). The effectiveness of cognitive behavioral therapy-based group counseling on depressive symptomatology, anxiety levels, automatic thoughts, and coping ways Turkish nursing students: A randomized controlled trial. Perspectives in psychiatric care. doi:10.1111/ppc.13073

Dennis CL, Grigoriadis S, Zupancic J, Kiss A, Ravitz P. Telephone-based nurse-delivered interpersonal psychotherapy for postpartum depression: nationwide randomised controlled trial. Br J Psychiatry. 2020;216(4):189-196.

Desautels, C., Savard, J., Ivers, H., Savard, M. H., & Caplette-Gingras, A. (2018). Treatment of depressive symptoms in patients with breast cancer: a randomized controlled trial comparing cognitive therapy and bright light therapy [Journal Article; Randomized Controlled Trial]. Health Psychology, 37(1), 1-13. https://doi.org/10.1037/hea0000539

Dimidjian S, Hollon SD, Dobson KS, Schmaling KB, Kohlenberg RJ, Addis ME, et al. Randomized trial of behavioral activation, cognitive therapy, and antidepressant medication in the acute treatment of adults with major depression. Journal of Consulting and Clinical Psychology. 2006;74(4):658-70.

Dimidjian, S., Goodman, S., Sherwood, N., Simon, G., Ludman, E., Gallop, R., . . . Beck, A. (2017). A Pragmatic Randomized Clinical Trial of Behavioral Activation for Depressed Pregnant Women. Journal of Consulting and Clinical Psychology, 85(1), 26-36

Dindo L, Recober A, Marchman JN, Turvey C, O'Hara MW. One-day behavioral treatment for patients with comorbid depression and migraine: A pilot study. Behaviour Research and Therapy. 2012;50(9):537-43.

Dindo, L. N., Recober, A., Calarge, C. A., Zimmerman, B. M., Weinrib, A., Marchman, J. N., & Turvey, C. (2019). One-Day Acceptance and Commitment Therapy Compared to Support for Depressed Migraine Patients: a Randomized Clinical Trial. Neurotherapeutics. doi:10.1007/s13311-019-00818-0

Dobkin RD, Mann SL, Gara MA, Interian A, Rodriguez KM, Menza M. Telephone-based cognitive behavioral therapy for depression in Parkinson disease: A randomized controlled trial. Neurology. 2020;94(16):e1764-e1773.

Dobkin RD, Menza M, Allen LA, Gara MA, Mark MH, Tiu J, et al. Cognitive-behavioral therapy for depression in Parkinson's disease: A randomized, controlled trial. American Journal of Psychiatry. 2011;168(10):1066-74.

Dobkin, R. D., Mann, S. L., Weintraub, D., Rodriguez, K. M., Miller, R. B., St. Hill, L., King, A., Gara, M. A., & Interian, A. (2021). Innovating Parkinson's Care: A Randomized Controlled Trial of Telemedicine Depression Treatment [Article]. Movement disorders, 36(11), 2549-2558. https://doi.org/10.1002/mds.28548

Dong, X., Sun, G., Zhan, J., Liu, F., Ma, S., Li, P., . . . Liu, Y. (2019). Telephone-based reminiscence therapy for colorectal cancer patients undergoing postoperative chemotherapy complicated with depression: a three-arm randomised controlled trial. Supportive care in cancer : official journal of the Multinational Association of Supportive Care in Cancer, 27(8), 2761-2769. doi:10.1007/s00520-018-4566-6

Dowrick, C., Dunn, G., Ayuso-Mateos, J. L., Dalgard, O. S., Page, H., Lehtinen, V., ... Wilkinson, G. (2000). Problem solving treatment and group psychoeducation for depression: multicentre randomised controlled trial Outcomes of Depression International Network [ODIN] Group. Bmj, 321(7274), 1450-1454. Retrieved from http://onlinelibrary.wiley.com/o/cochrane/clcentral/articles/836/CN-00623836/frame.html

Duarte PS, Miyazaki MC, Blay SL, Sesso R. Cognitive-behavioral group therapy is an effective treatment for major depression in hemodialysis patients. Kidney international. 2009;76(4):414-21.

Dunn NJ, Rehm LP, Schillaci J, Souchek J, Mehta P, Ashton CM, et al. A randomized trial of self-management and psychoeducational group therapies for comorbid chronic posttraumatic stress disorder and depressive disorder. Journal of traumatic stress. 2007;20(3):221-37.

Dwight-Johnson M, Aisenberg E, Golinelli D, Hong S, O'Brien M, Ludman E. Telephone-based cognitive-behavioral therapy for Latino patients living in rural areas: A randomized pilot study. Psychiatric Services. 2011;62(8):936-42.

Ebert DD, Buntrock C, Lehr D, et al. Effectiveness of Web- and Mobile-Based Treatment of Subthreshold Depression With Adherence-Focused Guidance: a Single-Blind Randomized Controlled Trial. Behavior therapy 2018; 49(1): 71-83.

Ebert, D. D., Lehr, D., Boß, L., Riper, H., Cuijpers, P., Andersson, G., . . . Berking, M. (2014). Efficacy of an internet-based problem-solving training for teachers: results of a randomized controlled trial. Scandinavian journal of work, environment & health, 582-596.

Ede MO, Igbo JN, Eseadi C, et al. Effect of group cognitive behavioural therapy on depressive symptoms in a sample of college adolescents in Nigeria. Journal of Rational-Emotive & Cognitive-Behavior Therapy. 2020;38(3):306-318.

Ekers D, Richards D, McMillan D, Bland JM, Gilbody S. Behavioural activation delivered by the non-specialist: Phase II randomised controlled trial. The British Journal of Psychiatry. 2011;198(1):66-72.

Ekkers W, Korrelboom K, Huijbrechts I, Smits N, Cuijpers P, Gaag M. Competitive memory training for treating depression and rumination in depressed older adults: A randomized controlled trial. Behaviour Research and Therapy. 2011;49(10):588-96.

Elkin I, Shea MT, Watkins JT, Imber SD, Sotsky SM, Collins JF, et al. National institute of mental health treatment of depression collaborative research program. General effectiveness of treatments. Archives of General Psychiatry. 1989;46(11):971-82; discussion 83.

Embling S. The effectiveness of cognitive behavioural therapy in depression. Nursing Standard. 2002;17(14-15):33-41.

Eseadi C, Obidoa MA, Ogbuabor SE, Ikechukwu-Ilomuanya AB. Effects of Group-Focused Cognitive-Behavioral Coaching Program on Depressive Symptoms in a Sample of Inmates in a Nigerian Prison. International journal of offender therapy and comparative criminology 2018; 62(6): 1589-602.

Eseadi, C., Ilechukwu, L. C., Victor-Aigbodion, V., Sewagegn, A. A., & Amedu, A. N. (2022). Intervention for depression among undergraduate religious education students: A randomized controlled trial. Medicine (Baltimore), 101(41), e31034. doi:10.1097/md.0000000000031034

Euteneuer, F., Dannehl, K., Del Rey, A., Engler, H., Schedlowski, M., & Rief, W. (2017). Immunological effects of behavioral activation with exercise in major depression: An exploratory randomized controlled trial. Translational Psychiatry, 7(5).

Evans RL, Connis RT. Comparison of brief group therapies for depressed cancer patients receiving radiation treatment. Public Health Reports. 1995;110(3):306-11.

Ewais, T., et al. (2021). "Mindfulness based cognitive therapy for youth with inflammatory bowel disease and depression - Findings from a pilot randomised controlled trial." J Psychosom Res 149: 110594.

Fann JR, Bombardier CH, Vannoy S, Dyer J, Ludman E, Dikmen S, et al. Telephone and in-person cognitive behavioral therapy for major depression after traumatic brain injury: A randomized controlled trial. Journal of neurotrauma. 2015;32(1):45-57.

Faramarzi M, Alipor A, Esmaelzadeh S, Kheirkhah F, Poladi K, Pash H. Treatment of depression and anxiety in infertile women: Cognitive behavioral therapy versus fluoxetine. Journal of Affective Disorders. 2008;108(1-2):159-64.

Fereydouni, S., & Forstmeier, S. (2022). An Islamic Form of Logotherapy in the Treatment of Depression, Anxiety and Stress Symptoms in University Students in Iran. J Relig Health, 61(1), 139-157. doi:10.1007/s10943-021-01495-0

Fissler, M., Winnebeck, E., Schroeter, T. A., Gummbersbach, M., Huntenburg, J. M., Gärtner, M., & Barnhofer, T. (2017). Brief training in mindfulness may normalize a blunted error-related negativity in chronically depressed patients. Cognitive, Affective & Behavioral Neuroscience, 17(6), 1164-1175. doi:10.3758/s13415-017-0540-x

Fledderus M, Bohlmeijer ET, Pieterse ME, Schreurs KM. Acceptance and commitment therapy as guided self-help for psychological distress and positive mental health: A randomized controlled trial. Psychological Medicine. 2012;42(3):485-95.

Floyd M, Scogin F, McKendree-Smith NL, Floyd DL, Rokke PD. Cognitive therapy for depression: A comparison of individual psychotherapy and bibliotherapy for depressed older adults. Behavior modification. 2004;28(2):297-318.

Flygare AL, Engström I, Hasselgren M, et al. Internet-based CBT for patients with depressive disorders in primary and psychiatric care: Is it effective and does comorbidity affect outcome? Internet Interventions. 2020;19.

Folke F, Parling T, Melin L. Acceptance and commitment therapy for depression: A preliminary randomized clinical trial for unemployed on long-term sick leave. Cognitive and Behavioral Practice. 2012;19(4):583-94.

Fonagy P, Lemma A, Target M, O'Keeffe S, Constantinou MP, Ventura Wurman T, et al. Dynamic interpersonal therapy for moderate to severe depression: a pilot randomized controlled and feasibility trial. Psychological medicine. 2019:1-10.

Fonagy P, Rost F, Carlyle JA, McPherson S, Thomas R, Pasco Fearon RM, et al. Pragmatic randomized controlled trial of long-term psychoanalytic psychotherapy for treatment-resistant depression: The Tavistock Adult Depression Study (TADS). World Psychiatry. 2015;14(3):312-21.

Forand NR, Barnett JG, Strunk DR, Hindiyeh MU, Feinberg JE, Keefe JR. Efficacy of Guided iCBT for Depression and Mediation of Change by Cognitive Skill Acquisition. Behavior Therapy 2018; 49(2): 295-307.

Forsell E, Bendix M, Holländare F, et al. Internet delivered cognitive behavior therapy for antenatal depression: A randomised controlled trial. Journal of Affective Disorders 2017; 221: 56-64.

Frangou, E., Bertelli, G., Love, S., Mackean, M. J., Glasspool, R. M., Fotopoulou, C., Cook, A., Nicum, S., Lord, R., Ferguson, M., Roux, R. L., Martinez, M., Butcher, C., Hulbert-Williams, N., Howells, L., & Blagden, S. P. (2021). OVPSYCH2: A randomized controlled trial of psychological support versus standard of care following chemotherapy for ovarian cancer. Gynecol Oncol, 162(2), 431-439. https://doi.org/10.1016/j.ygyno.2021.05.024

Freedland KE, Carney RM, Rich MW, Steinmeyer BC, Rubin EH. Cognitive behavior therapy for depression and self-care in heart failure patients: A randomized clinical trial. JAMA Internal Medicine. 2015;175(11):1773-82.

Freedland KE, Skala JA, Carney RM, Rubin EH, Lustman PJ, D·vila-Rom·n VG, et al. Treatment of depression after coronary artery bypass surgery: A randomized controlled trial. Archives of General Psychiatry. 2009;66(4):387-96.

Funderburk, J. S., Pigeon, W. R., Shepardson, R. L., Wade, M., Acker, J., Fivecoat, H., Wray, L. O., & Maisto, S. A. (2021). Treating depressive symptoms among veterans in primary care: A multi-site RCT of brief behavioral activation. J Affect Disord, 283, 11-19. https://doi.org/10.1016/j.jad.2021.01.033

Furukawa TA, Horikoshi M, Kawakami N, Kadota M, Sasaki M, Sekiya Y, et al. Telephone cognitive-behavioral therapy for subthreshold depression and presenteeism in workplace: A randomized controlled trial. PLoS One. 2012;7(4):e35330.

Garcia, A., Yáñez, A. M., Bennasar-Veny, M., Navarro, C., Salva, J., Ibarra, O., . . . Garcia-Toro, M. (2023). Efficacy of an adjuvant non-face-to-face multimodal lifestyle modification program for patients with treatment-resistant major depression: A randomized controlled trial. Psychiatry Res, 319, 114975. doi:10.1016/j.psychres.2022.114975

Gawrysiak M, Nicholas C, Hopko DR. Behavioral activation for moderately depressed university students: Randomized controlled trial. Journal of Counseling Psychology. 2009;56(3):468-75.

Gellis ZD, Bruce ML. Problem solving therapy for subthreshold depression in home healthcare patients with cardiovascular disease. The American Journal of Geriatric Psychiatry. 2010;18(6):464-74.

Gellis ZD, McGinty J, Tierney L, Jordan C, Burton J, Misener E. Randomized controlled trial of problem-solving therapy for minor depression in home care. Research on Social Work Practice. 2008;18(6):596-606.

Geraedts AS, Kleiboer AM, Wiezer NM, van Mechelen W, Cuijpers P. Short-term effects of a web-based guided self-help intervention for employees with depressive symptoms: Randomized controlled trial. Journal of Medical Internet Research. 2014;16(5):e121.

Ghorbani, V., Zanjani, Z., Omidi, A., & Sarvizadeh, M. (2021). Efficacy of acceptance and commitment therapy (ACT) on depression, pain acceptance, and psychological flexibility in married women with breast cancer: a pre- and post-test clinical trial. Trends Psychiatry Psychother, 43(2), 126-133. https://doi.org/10.47626/2237-6089-2020-0022

Gibbons MB, Thompson SM, Scott K, Schauble LA, Mooney T, Thompson D, et al. Supportive-expressive dynamic psychotherapy in the community mental health system: A pilot effectiveness trial for the treatment of depression. Psychotherapy (Chicago, Ill). 2012;49(3):303-16.

Gitlin LN, Harris LF, McCoy MC, Chernett NL, Pizzi LT, Jutkowitz E, et al. A home-based intervention to reduce depressive symptoms and improve quality of life in older African Americans: A randomized trial. Annals of Internal Medicine. 2013;159(4):243-52.

Goodman JH, Prager J, Goldstein R, Freeman M. Perinatal Dyadic Psychotherapy for postpartum depression: A randomized controlled pilot trial. Archives of Women's Mental Health. 2015;18(3):493-506.

Greenberg J, Datta T, Shapero BG, Sevinc G, Mischoulon D, Lazar SW. Compassionate hearts protect against wandering minds: Self-compassion moderates the effect of mind-wandering on depression. Spirituality in Clinical Practice 2018; 5(3): 155-69.

Grote NK, Swartz HA, Geibel SL, Zuckoff A, Houck PR, Frank E. A randomized controlled trial of culturally relevant, brief interpersonal psychotherapy for perinatal depression. Psychiatric Services. 2009;60(3):313-21.

Gumley, A., White, R., Briggs, A., Ford, I., Barry, S., Stewart, C., . . . McLeod, H. (2017). A parallel group randomised open blinded evaluation of Acceptance and Commitment Therapy for depression after psychosis: pilot trial outcomes (ADAPT). Schizophrenia research, 183, 143-150. Retrieved from http://onlinelibrary.wiley.com/o/cochrane/clcentral/articles/430/CN-01373430/frame.html doi:10.1016/j.schres.2016.11.026

Gureje, O., Oladeji, B. D., Kola, L., Bello, T., Ayinde, O., Faregh, N., . . . Zelkowitz, P. (2022). Effect of intervention delivered by frontline maternal care providers to improve outcome and parenting skills among adolescents with perinatal depression in Nigeria (the RAPiD study): A cluster randomized controlled trial. J Affect Disord, 312, 169-176. doi:10.1016/j.jad.2022.06.032

Hagen, R., Hjemdal, O., Solem, S., Kennair, L. E. O., Nordahl, H. M., Fisher, P., & Wells, A. (2017). Metacognitive therapy for depression in adults: A waiting list randomized controlled trial with six months follow-up. Frontiers in Psychology, 8.

Hallford DJ, Mellor D. Autobiographical memory-based intervention for depressive symptoms in young adults: A randomized controlled trial of cognitive-reminiscence therapy. Psychotherapy and Psychosomatics. 2016;85(4):246-9.

Hallgren M, Kraepelien M, Öjehagen A, Lindefors N, Zeebari Z, Kaldo V, et al. Physical exercise and internet-based cognitive-behavioural therapy in the treatment of depression: Randomised controlled trial. British Journal of Psychiatry. 2015;207(3):227-34.

Hamamci Z. Integrating psychodrama and cognitive behavioral therapy to treat moderate depression. Arts in Psychotherapy. 2006;33(3):199-207.

Hamdan-Mansour AM, Puskar K, Bandak AG. Effectiveness of cognitive-behavioral therapy on depressive symptomatology, stress and coping strategies among Jordanian university students. Issues in mental health nursing. 2009;30(3):188-96.

Han YMY, Sze SL, Wong QY, Chan AS. A mind-body lifestyle intervention enhances emotional control in patients with major depressive disorder: a randomized, controlled study. Cognitive, affective & behavioral neuroscience. 2020;20(5):1056-1069.

Haringsma R, Engels G, Cuijpers P, Spinhoven P. Effectiveness of the Coping With Depression (CWD) course for older adults provided by the community-based mental health care system in the Netherlands: A randomized controlled field trial. International Psychogeriatrics. 2006;18(02):307-25.

Harley R, Sprich S, Safren S, Jacobo M, Fava M. Adaptation of dialectical behavior therapy skills training group for treatment-resistant depression. Journal of Nervous and Mental Disease. 2008;196(2):136-43.

Harrer, M., Apolinário-Hagen, J., Fritsche, L., Salewski, C., Zarski, A. C., Lehr, D., Baumeister, H., Cuijpers, P., & Ebert, D. D. (2021). Effect of an internet- and app-based stress intervention compared to online psychoeducation in university students with depressive symptoms: Results of a randomized controlled trial [Article]. Internet Interventions, 24. https://doi.org/10.1016/j.invent.2021.100374

Hashemi, Z., Eyni, S., & Ebadi, M. (2022). Effectiveness of Acceptance and Commitment Therapy in Depression and Anxiety in People with Substance Use Disorder. Iranian Journal of Psychiatry and Behavioral Sciences, 16(1). doi:10.5812/ijpbs.110135

Hassiotis A, Serfaty M, Azam K, Strydom A, Blizard R, Romeo R, et al. Manualised individual cognitive behavioural therapy for mood disorders in people with mild to moderate intellectual disability: A feasibility randomised controlled trial. Journal of Affective Disorders. 2013;151(1):186-95.

Hautzinger M, Welz S. Kognitive Verhaltenstherapie bei Depressionen im Alter: Ergebnisse einer kontrollierten Vergleichsstudie unter ambulanten Bedingungen an Depressionen mittleren Schweregrads. = Cognitive behavioral therapy for depressed older outpatients: A controlled, randomized trial. Zeitschrift für Gerontologie und Geriatrie. 2004;37(6):427-35.

Heckman TG, Sikkema KJ, Hansen N, Kochman A, Heh V, Neufeld S, et al. A randomized clinical trial of a coping improvement group intervention for HIV-infected older adults. Journal of behavioral medicine. 2011;34(2):102-11.

Heckman, T. G., Heckman, B. D., Anderson, T., Lovejoy, T. I., Mohr, D., Sutton, M., . . . Gau, J.-T. (2013). Supportive-expressive and coping group teletherapies for HIV-infected older adults: a randomized clinical trial. AIDS Behav, 17(9), 3034-3044.

Heim, E., Ramia, J. A., Hana, R. A., Burchert, S., Carswell, K., Cornelisz, I., . . . van't Hof, E. (2021). Step-by-step: Feasibility randomised controlled trial of a mobile-based intervention for depression among populations affected by adversity in Lebanon. Internet Interventions, 24, 100380. doi:https://doi.org/10.1016/j.invent.2021.100380

Hemanny, C., Carvalho, C., Maia, N., Reis, D., Botelho, A. C., Bonavides, D., . . . De Oliveira, I. R. (2019). Efficacy of trial-based cognitive therapy, behavioral activation and treatment as usual in the treatment of major depressive disorder: Preliminary findings from a randomized clinical trial. CNS Spectrums. doi:10.1017/S1092852919001457

Hermanns N, Schmitt A, Gahr A, Herder C, Nowotny B, Roden M, et al. The effect of a diabetes-specific cognitive behavioral treatment program (DIAMOS) for patients with diabetes and subclinical depression: Results of a randomized controlled trial. Diabetes care. 2015;38(4):551-60.

Herrmann-Lingen C, Beutel ME, Bosbach A, Deter HC, Fritzsche K, Hellmich M, et al. A stepwise psychotherapy intervention for reducing risk in coronary artery disease (SPIRR-CAD): Results of an observer-blinded, multicenter, randomized trial in depressed patients with coronary artery disease. Psychosomatic Medicine. 2016;78(6):704-15.

Hoifodt RS, Lillevoll KR, Griffiths KM, et al. The clinical effectiveness of web-based cognitive behavioral therapy with face-to-face therapist support for depressed primary care patients: randomized controlled trial. Journal of medical Internet research 2013; 15(8): e153.

Honey KL, Bennett P, Morgan M. A brief psycho-educational group intervention for postnatal depression. British Journal of Clinical Psychology. 2002;41(4):405-9.

Horrell L, Goldsmith KA, Tylee AT, Schmidt UH, Murphy CL, Bonin E-M, et al. One-day cognitive–behavioural therapy self-confidence workshops for people with depression: Randomised controlled trial. The British Journal of Psychiatry. 2014;204(3):222-33.

Hou Y, Hu P, Zhang Y, Lu Q, Wang D, Yin L, et al. Cognitive behavioral therapy in combination with systemic family therapy improves mild to moderate postpartum depression. Revista Brasileira de Psiquiatria. 2014;36(1):47-52.

Hsiao, F.-H., Lai, Y.-M., Chen, Y.-T., Yang, T.-T., Liao, S.-C., Ho, R. T., Ng, S.-M., Chan, C. L., & Jow, G.-M. (2014). Efficacy of psychotherapy on diurnal cortisol patterns and suicidal ideation in adjustment disorder with depressed mood. General hospital psychiatry, 36(2), 214-219. http://ac.els-cdn.com/S0163834313003113/1-s2.0-S0163834313003113-main.pdf?_tid=685fbc62-cf4e-11e5-b7e9-00000aab0f6c&acdnat=1455037317_a8c762fea010c7ac31f2152769dad94c

Huang, C.-Y., Lai, H.-L., Chen, C.-I., Lu, Y.-C., Li, S.-C., Wang, L.-W., & Su, Y. (2016). Effects of motivational enhancement therapy plus cognitive behaviour therapy on depressive symptoms and health-related quality of life in adults with type II diabetes mellitus: A randomised controlled trial. Quality of Life Research: An International Journal of Quality of Life Aspects of Treatment, Care & Rehabilitation, 25(5), 1275-1283.

Hum, K. M., Chan, C. J., Gane, J., Conway, L., McAndrews, M. P., & Smith, M. L. (2019). Do distance-delivery group interventions improve depression in people with epilepsy? Epilepsy & Behavior, 98, 153-160. doi:10.1016/j.yebeh.2019.06.037

Hummel, J., Weisbrod, C., Boesch, L., Himpler, K., Hauer, K., Hautzinger, M., . . . Kopf, D. (2017). AIDE–Acute Illness and Depression in Elderly Patients. Cognitive Behavioral Group Psychotherapy in Geriatric Patients With Comorbid Depression: A Randomized, Controlled Trial. Journal of the american medical directors association, 18(4), 341-349.

Husain, N., Kiran, T., Fatima, B., Chaudhry, I. B., Husain, M., Shah, S., Bassett, P., Cohen, N., Jafri, F., Naeem, S., Zadeh, Z., Roberts, C., Rahman, A., Naeem, F., Husain, M. I., & Chaudhry, N. (2021). An integrated parenting intervention for maternal depression and child development in a low-resource setting: Cluster randomized controlled trial [Article]. Depress Anxiety, 38(9), 925-939. https://doi.org/10.1002/da.23169

Husain, N., Kiran, T., Shah, S., Rahman, A., Raza Ur, R., Saeed, Q., Naeem, S., Bassett, P., Husain, M., Haq, S. U., Jaffery, F., Cohen, N., Naeem, F., & Chaudhry, N. (2021). Efficacy of learning through play plus intervention to reduce maternal depression in women with malnourished children: A randomized controlled trial from Pakistan(☆). J Affect Disord, 278, 78-84. https://doi.org/10.1016/j.jad.2020.09.001

Husain, N., Zulqernain, F., Carter, L.-A., Chaudhry, I., Fatima, B., Kiran, T., . . . Rahman, A. (2017). Treatment of maternal depression in urban slums of Karachi, Pakistan: a randomized controlled trial (RCT) of an integrated maternal psychological and early child development intervention. Asian journal of psychiatry, 29, 63-70.

Jalali, F., Hasani, A., Hashemi, S. F., Kimiaei, S. A., & Babaei, A. (2019). Cognitive Group Therapy Based on Schema-Focused Approach for Reducing Depression in Prisoners Living With HIV. International journal of offender therapy and comparative criminology, 63(2), 276-288. doi:10.1177/0306624X18784185

Jamison C, Scogin F. The outcome of cognitive bibliotherapy with depressed adults. Journal of Consulting and Clinical Psychology. 1995;63(4):644-50.

Jarrett RB, Schaffer M, McIntire D, Witt-Browder A, Kraft D, Risser RC. Treatment of atypical depression with cognitive therapy or phenelzine: A double-blind, placebo-controlled trial. Archives of General Psychiatry. 1999;56(5):431-7.

Jelinek L, Hauschildt M, Wittekind CE, Schneider BC, Kriston L, Moritz S. Efficacy of metacognitive training for depression: A randomized controlled trial. Psychotherapy and Psychosomatics. 2016;85(4):231-4.

Jesse DE, Gaynes BN, Feldhousen EB, Newton ER, Bunch S, Hollon SD. Performance of a culturally tailored cognitive-behavioral intervention integrated in a public health setting to reduce risk of antepartum depression: A randomized controlled trial. Journal of Midwifery and Women's Health. 2015;60(5):578-92.

Jiang L, Wang ZZ, Qiu LR, Wan GB, Lin Y, Wei Z. Psychological intervention for postpartum depression. Journal of Huazhong University of Science and Technology: Medical sciences. 2014;34(3):437-42.

Johansson R, Ekbladh S, Hebert A, Lindström M, Möller S, Petitt E, et al. Psychodynamic guided self-help for adult depression through the internet: A randomised controlled trial. PloS One. 2012;7(5):e38021.

Johansson R, Sjöberg E, Sjögren M, Johnsson E, Carlbring P, Andersson T, et al. Tailored vs. standardized internet-based cognitive behavior therapy for depression and comorbid symptoms: A randomized controlled trial. PloS One. 2012;7(5):e36905.

Johansson, O., Bjärehed, J., Andersson, G., Carlbring, P., & Lundh, L. G. (2019). Effectiveness of guided internet-delivered cognitive behavior therapy for depression in routine psychiatry: A randomized controlled trial. Internet Interventions, 17. doi:10.1016/j.invent.2019.100247

Johnson JE, Zlotnick C. Pilot study of treatment for major depression among women prisoners with substance use disorder. Journal of Psychiatric Research. 2012;46(9):1174-83.

Joling KJ, Hout HP, van't Veer-Tazelaar PJ, Horst HE, Cuijpers P, Ven PM, et al. How effective is bibliotherapy for very old adults with subthreshold depression? A randomized controlled trial. American Journal of Geriatric Psychiatry. 2011;19(3):256-65.

Kamga, H., McCusker, J., Yaffe, M., Sewitch, M., Sussman, T., Strumpf, E., . . . Freeman, E. (2017). Self-care tools to treat depressive symptoms in patients with age-related eye disease: a randomized controlled clinical trial. Clinical & experimental ophthalmology, 45(4), 371-378.

Kanter JW, Santiago-Rivera AL, Santos MM, Nagy G, López M, Hurtado GD, et al. A randomized hybrid efficacy and effectiveness trial of behavioral activation for latinos with depression. Behavior Therapy. 2015;46(2):177-92.

Kay-Lambkin, F. J., Baker, A. L., Lewin, T. J., & Carr, V. J. (2009). Computer-based psychological treatment for comorbid depression and problematic alcohol and/or cannabis use: A randomized controlled trial of clinical efficacy. Addiction, 104(3), 378-388. doi:10.1111/j.1360-0443.2008.02444.x

Keeley RD, Brody DS, Engel M, Burke BL, Nordstrom K, Moralez E, et al. Motivational interviewing improves depression outcome in primary care: A cluster randomized trial. Journal of Consulting and Clinical Psychology. 2016;84(11):993-1007.

Kelly JA, Murphy DA, Bahr GR, Kalichman SC, Morgan MG, Stevenson LY, et al. Outcome of cognitive-behavioral and support group brief therapies for depressed, HIV-infected persons. American Journal of Psychiatry. 1993;150(11):1679-86.

Kemmeren, L. L., van Schaik, A., Draisma, S., Kleiboer, A., Riper, H., & Smit, J. H. (2023). Effectiveness of Blended Cognitive Behavioral Therapy Versus Treatment as Usual for Depression in Routine Specialized Mental Healthcare: E-COMPARED Trial in the Netherlands. Cognitive Therapy and Research. doi:10.1007/s10608-023-10363-y

Kenter, R. M. F., Cuijpers, P., Beekman, A., & van Straten, A. (2016). Effectiveness of a Web-based guided self-help intervention for outpatients with a depressive disorder: Short-term results from a randomized controlled trial. Journal of medical Internet research, 18(3).

Kessler D, Lewis G, Kaur S, Wiles N, King M, Weich S, et al. Therapist-delivered Internet psychotherapy for depression in primary care: A randomised controlled trial. Lancet. 2009;374(9690):628-34.

Khazraee, H., Bakhtiari, M., Kianimoghadam, A. S., & Ghorbanikhah, E. (2023). The Effectiveness of Mindful Hypnotherapy on Depression, Self-Compassion, and Psychological Inflexibility in Females with Major Depressive Disorder: A Single-Blind, Randomized Clinical Trial. Int J Clin Exp Hypn, 71(1), 63-78. doi:10.1080/00207144.2022.2160257

Khoshbooii, R., Hassan, S. A., Deylami, N., Muhamad, R., Engku Kamarudin, E. M., & Alareqe, N. A. (2021). Effects of Group and Individual Culturally Adapted Cognitive Behavioral Therapy on Depression and Sexual Satisfaction among Perimenopausal Women. Int J Environ Res Public Health, 18(14). https://doi.org/10.3390/ijerph18147711

Kim YH, Choi KS, Han K, Kim HW. A psychological intervention programme for patients with breast cancer under chemotherapy and at a high risk of depression: a randomised clinical trial. Journal of clinical nursing 2018; 27(3-4): 572-81.

King M, Sibbald B, Ward E, Bower P, Lloyd M, Gabbay M, et al. Randomised controlled trial of non-directive counselling, cognitive-behaviour therapy and usual general practitioner care in the management of depression as well as mixed anxiety and depression in primary care. Health Technology Assessment. 2000;4(19):1-83.

Korrelboom K, Maarsingh M, Huijbrechts I. Competitive memory training (COMET) for treating low self-esteem in patients with depressive disorders: A randomized clinical trial. Depression and anxiety. 2012;29(2):102-10.

Korte J, Bohlmeijer ET, Cappeliez P, Smit F, Westerhof GJ. Life review therapy for older adults with moderate depressive symptomatology: A pragmatic randomized controlled trial. Psychological Medicine. 2012;42(6):1163-73.

Kramer, J., Conijn, B., Oijevaar, P., & Riper, H. (2014). Effectiveness of a web-based solution-focused brief chat treatment for depressed adolescents and young adults: randomized controlled trial. J Med Internet Res, 16(5), e141. doi:10.2196/jmir.3261

Krämer, L. V., Grünzig, S. D., Baumeister, H., Ebert, D. D., & Bengel, J. (2021). Effectiveness of a Guided Web-Based Intervention to Reduce Depressive Symptoms before Outpatient Psychotherapy: A Pragmatic Randomized Controlled Trial. Psychother Psychosom, 90(4), 233-242. https://doi.org/10.1159/000515625

Laidlaw K, Davidson K, Toner H, Jackson G, Clark S, Law J, et al. A randomised controlled trial of cognitive behaviour therapy vs treatment as usual in the treatment of mild to moderate late life depression. International Journal of Geriatric Psychiatry. 2008;23(8):843-50.

Lamers F, Jonkers CC, Bosma H, Kempen GI, Meijer JA, Penninx BW, et al. A minimal psychological intervention in chronically ill elderly patients with depression: A randomized trial. Psychotherapy and Psychosomatics. 2010;79(4):217-26.

Lamers SMA, Bohlmeijer ET, Korte J, Westerhof GJ. The efficacy of life-review as online-guided self-help for adults: A randomized trial. The Journals of Gerontology: Series B: Psychological Sciences and Social Sciences 2015; 70B(1): 24-34.

Landreville P, Bissonnette L. Effects of cognitive bibliotherapy for depressed older adults with a disability. Clinical Gerontologist. 1997;17(4):35-55.

Lappalainen P, Langrial S, Oinas-Kukkonen H, Tolvanen A, Lappalainen R. Web-based acceptance and commitment therapy for depressive symptoms with minimal support: A randomized controlled trial. Behavior modification. 2015;39(6):805-34.

Larcombe NA, Wilson PH. An evaluation of cognitive-behaviour therapy for depression in patients with multiple sclerosis. The British Journal of Psychiatry. 1984;145:366-71.

Lee, E., Han, Y., Cha, Y. J., Oh, J. H., Hwang, N. R., Seo, H. J., & Choi, K. H. (2021). Community-Based Multi-Site Randomized Controlled Trial of Behavioral Activation for Patients with Depressive Disorders [Article in Press]. Community mental health journal. https://doi.org/10.1007/s10597-021-00828-3

Lemma A, Fonagy P. Feasibility study of a psychodynamic online group intervention for depression. Psychoanalytic Psychology. 2013;30(3):367-80.

Lenze SN, Potts MA, Rodgers J, Luby J. Lessons learned from a pilot randomized controlled trial of dyadic interpersonal psychotherapy for perinatal depression in a low-income population. Journal of Affective Disorders. 2020;271:286-292.

Lenze, S., & Potts, M. (2017). Brief Interpersonal Psychotherapy for depression during pregnancy in a low-income population: a randomized controlled trial. Journal of Affective Disorders, 210, 151-157.

Lerner D, Adler DA, Rogers WH, Chang H, Greenhill A, Cymerman E, et al. A randomized clinical trial of a telephone depression intervention to reduce employee presenteeism and absenteeism. Psychiatric Services. 2015;66(6):570-7.

Leung S, Lee A, Wong D, Wong C, Leung K, Chiang V, et al. A brief group intervention using a cognitive-behavioural approach to reduce postnatal depressive symptoms: A randomised controlled trial. Hong Kong Medical Journal. 2016;22(1 Supplement 2).

Leung SS, Lee AM, Chiang VC, Lam SK, Kuen YW, Wong DF. Culturally sensitive, preventive antenatal group cognitive-behavioural therapy for Chinese women with depression. Internationan Journal of Nursing Practice. 2013;19(Supp 1):28-37.

Lexis MA, Jansen NW, Huibers MJ, Amelsvoort LG, Berkouwer A, Tjin ATG, et al. Prevention of long-term sickness absence and major depression in high-risk employees: A randomised controlled trial. Occupational and Environmental Medicine. 2011;68(6):400-7.

Liang, L., Feng, L., Zheng, X., Wu, Y., Zhang, C., & Li, J. (2021). Effect of dialectical behavior group therapy on the anxiety and depression of medical students under the normalization of epidemic prevention and control for the COVID-19 epidemic: a randomized study. Ann Palliat Med, 10(10), 10591-10599. https://doi.org/10.21037/apm-21-2466

Liu ET-H, Chen W-L, Li Y-H, Wang CH, Mok TJ, Huang HS. Exploring the efficacy of cognitive bibliotherapy and a potential mechanism of change in the treatment of depressive symptoms among the Chinese: A randomized controlled trial. Cognitive Therapy and Research. 2009;33(5):449-61.

Liu, H., & Yang, Y. (2021). Effects of a psychological nursing intervention on prevention of anxiety and depression in the postpartum period: a randomized controlled trial [Article]. Annals of General Psychiatry, 20(1). https://doi.org/10.1186/s12991-020-00320-4

Lloyd-Williams M, Shiels C, Ellis J, et al. Pilot randomised controlled trial of focused narrative intervention for moderate to severe depression in palliative care patients: DISCERN trial. Palliative medicine 2018; 32(1): 206-15.

Lök, N., Bademli, K., & Selçuk-Tosun, A. (2019). The effect of reminiscence therapy on cognitive functions, depression, and quality of life in Alzheimer patients: randomized controlled trial. International Journal of Geriatric Psychiatry, 34(1), 47-53. doi:10.1002/gps.4980

Losada A, Márquez-González M, Romero-Moreno R, Mausbach BT, López J, Fernández-Fernández V, et al. Cognitive–behavioral therapy (CBT) versus acceptance and commitment therapy (ACT) for dementia family caregivers with significant depressive symptoms: Results of a randomized clinical trial. Journal of Consulting and Clinical Psychology. 2015;83(4):760-72.

Lovell K, Bower P, Richards D, Barkham Mi, Sibbald B, Roberts C, et al. Developing guided self-help for depression using the medical research council complex interventions framework: A description of the modelling phase and results of an exploratory randomised controlled trial. BMC psychiatry. 2008;8.

Lund, C., Schneider, M., Garman, E. C., Davies, T., Munodawafa, M., Honikman, S., . . . Susser, E. (2019). Task-sharing of psychological treatment for antenatal depression in Khayelitsha, South Africa: Effects on antenatal and postnatal outcomes in an individual randomised controlled trial. Behaviour Research and. doi:10.1016/j.brat.2019.103466

Lundgren JG, Dahlstrom O, Andersson G, Jaarsma T, Karner Kohler A, Johansson P. The effect of guided web-based cognitive behavioral therapy on patients with depressive symptoms and heart failure: A pilot randomized controlled trial. Journal of Medical Internet Research. 2016;18(8):e194.

Lynch, T. R., Hempel, R. J., Whalley, B., Byford, S., Chamba, R., Clarke, P., . . . Russell, I. T. (2019). Refractory depression - mechanisms and efficacy of radically open dialectical behaviour therapy (RefraMED): findings of a randomised trial on benefits and harms. The British journal of psychiatry : the journal of mental science, 1-9. doi:10.1192/bjp.2019.53

MacLean S, Corsi DJ, Litchfield S, et al. Coach-facilitated web-based therapy compared with information about web-based resources in patients referred to secondary mental health care for depression: Randomized controlled trial. Journal of Medical Internet Research. 2020;22(6).

MacPherson H, Richmond S, Bland M, Brealey S, Gabe R, Hopton A, et al. Acupuncture and counselling for depression in primary care: A randomised controlled trial. PLoS medicine. 2013;10(9):e1001518.

Mahmoodi, M., Bakhtiyari, M., Masjedi Arani, A., Mohammadi, A., & Saberi Isfeedvajani, M. (2021). The comparison between CBT focused on perfectionism and CBT focused on emotion regulation for individuals with depression and anxiety disorders and dysfunctional perfectionism: A randomized controlled trial. Behavioural and Cognitive Psychotherapy, 49(4), 454-471. https://doi.org/10.1017/S1352465820000909

Maina G, Forner F, Bogetto F. Randomized controlled trial comparing brief dynamic and supportive therapy with waiting list condition in minor depressive disorders. Psychotherapy and Psychosomatics. 2005;74(1):43-50.

Malouff JM, Lanyon RI, Schutte NS. Effectiveness of a brief group RET treatment for divorce-related dysphoria. Journal of Rational-Emotive and Cognitive-Behavior Therapy. 1988;6(3):162-71.

Mansour, N., Labib, N., Khalil, M., & Esmat, S. (2022). Brief Cognitive Behavioral Therapy for Patients with Comorbid Depression and Type 2 Diabetes in an Urban Primary Care Facility: Randomized Controlled Trial. Open Access Macedonian Journal of Medical Sciences, 10, 60-67. doi:10.3889/oamjms.2022.7883

Martin PR, Aiello R, Gilson K, Meadows G, Milgrom J, Reece J. Cognitive behavior therapy for comorbid migraine and/or tension-type headache and major depressive disorder: An exploratory randomized controlled trial. Behaviour Research and Therapy. 2015;73:8-18.

Matsuzaka, C., Wainberg, M., Norcini, P. A., Hoffmann, E., Coimbra, B., Braga, R., . . . Mello, M. (2017). Task shifting interpersonal counseling for depression: a pragmatic randomized controlled trial in primary care. BMC Psychiatry, 17(1)

McCusker, J., Jones, J. M., Li, M., Faria, R., Yaffe, M. J., Lambert, S. D., Ciampi, A., Belzile, E., & de Raad, M. (2021). CanDirect: Effectiveness of a Telephone-Supported Depression Self-Care Intervention for Cancer Survivors. J Clin Oncol, 39(10), 1150-1161. https://doi.org/10.1200/jco.20.01802

McIndoo CC, File AA, Preddy T, Clark CG, Hopko DR. Mindfulness-based therapy and behavioral activation: A randomized controlled trial with depressed college students. Behaviour Research and Therapy. 2016;77:118-28.

McKee MD, Zayas LH, Fletcher J, Boyd RC, Nam SH. Results of an intervention to reduce perinatal depression among low-income minority women in community primary care. Journal of Social Service Research. 2006;32(4):63-81.

Mennen, F. E., Palmer Molina, A., Monro, W. L., Duan, L., Stuart, S., & Sosna, T. (2021). Effectiveness of an Interpersonal Psychotherapy (IPT) Group Depression Treatment for Head Start Mothers: A Cluster-Randomized Controlled Trial. J Affect Disord, 280(Pt B), 39-48. https://doi.org/10.1016/j.jad.2020.11.074

Michalak J, Schultze M, Heidenreich T, Schramm E. A randomized controlled trial on the efficacy of mindfulness-based cognitive therapy and a group version of cognitive behavioral analysis system of psychotherapy for chronically depressed patients. Journal of Consulting and Clinical Psychology. 2015;83(5):951-63.

Milgrom J, Danaher BG, Gemmill AW, Holt C, Holt CJ, Seeley JR, et al. Internet cognitive behavioral therapy for women with postnatal depression: A randomized controlled trial of MumMoodBooster. Journal of Medical Internet Research. 2016;18(3):e54.

Milgrom J, Holt C, Holt CJ, Ross J, Ericksen J, Gemmill AW. Feasibility study and pilot randomised trial of an antenatal depression treatment with infant follow-up. Archives of Women's Mental Health. 2015;18(5):717-30.

Milgrom J, Holt CJ, Gemmill AW, Ericksen J, Leigh B, Buist A, et al. Treating postnatal depressive symptoms in primary care: A randomised controlled trial of GP management, with and without adjunctive counselling. BMC psychiatry. 2011;11:95.

Milgrom, J., Danaher, B. G., Seeley, J. R., Holt, C. J., Holt, C., Ericksen, J., Tyler, M. S., Gau, J. M., & Gemmill, A. W. (2021). Internet and Face-to-face Cognitive Behavioral Therapy for Postnatal Depression Compared With Treatment as Usual: Randomized Controlled Trial of MumMoodBooster. J Med Internet Res, 23(12), e17185. https://doi.org/10.2196/17185

Miller L, Weissman M. Interpersonal psychotherapy delivered over the telephone to recurrent depressives. A pilot study. Depression and anxiety. 2002;16(3):114-7.

Miranda J, Chung JY, Green BL, Krupnick J, Siddique J, Revicki DA, et al. Treating depression in predominantly low-income young minority women: A randomized controlled trial. Jama. 2003;290(1):57-65.

Mohr DC, Carmody T, Erickson L, Jin L, Leader J. Telephone-administered cognitive behavioral therapy for veterans served by community-based outpatient clinics. Journal of Consulting and Clinical Psychology. 2011;79(2):261-5.

Mohr DC, Duffecy J, Ho J, Kwasny M, Cai X, Burns MN, et al. A randomized controlled trial evaluating a manualized TeleCoaching protocol for improving adherence to a web-based intervention for the treatment of depression. PLoS One. 2013;8(8):e70086.

Mohr DC, Likosky W, Bertagnolli A, Goodkin DE, Van Der Wende J, Dwyer P, et al. Telephone-administered cognitive-behavioral therapy for the treatment of depressive symptoms in multiple sclerosis. Journal of Consulting and Clinical Psychology. 2000;68(2):356-61.

Moldovan R, Cobeanu O, David D. Cognitive bibliotherapy for mild depressive symptomatology: Randomized clinical trial of efficacy and mechanisms of change. Clinical Psychology and Psychotherapy. 2013;20(6):482-93.

Montero-Marín, J., Araya, R., Pérez-Yus, M. C., Mayoral, F., Gili, M., Botella, C., . . . López-Del-Hoyo, Y. (2016). An internet-based intervention for depression in primary Care in Spain: a randomized controlled trial. Journal of medical Internet research, 18(8), e231.

Moon, J. R., Huh, J., Song, J., Kang, I. S., Park, S. W., Chang, S. A., . . . Han, J. S. (2021). The effects of rational emotive behavior therapy for depressive symptoms in adults with congenital heart disease. Heart Lung, 50(6), 906-913. doi:10.1016/j.hrtlng.2021.07.011

Mukhtar, F., Oei, T. P., & Yaacob, M. (2011). Effectiveness of group cognitive behaviour therapy augmentation in reducing negative cognitions in the treatment of depression in Malaysia. ASEAN Journal of Psychiatry, 12(1), 50-65.

Mulcahy R, Reay RE, Wilkinson RB, Owen C. A randomised control trial for the effectiveness of group interpersonal psychotherapy for postnatal depression. Archives of Women's Mental Health. 2010;13(2):125-39.

Musa, Z. A., Soh, K. L., Mukhtar, F., Soh, K. Y., Oladele, T. O., & Soh, K. G. (2021). Effectiveness of mindfulness-based cognitive therapy among depressed individuals with disabilities in Nigeria: A randomized controlled trial. Psychiatry Res, 296, 113680. doi:10.1016/j.psychres.2020.113680

Mynors-Wallis L, Gath D, Lloyd-Thomas A, Tomlinson D. Randomised controlled trial comparing problem solving treatment with amitriptyline and placebo for major depression in primary care. BMJ. 1995;310(6977):441-5.

Nadort, E., Schouten, R. W., Boeschoten, R. E., Smets, Y., Chandie Shaw, P., Vleming, L. J., . . . Siegert, C. E. H. (2022). Internet-based treatment for depressive symptoms in hemodialysis patients: A cluster randomized controlled trial. Gen Hosp Psychiatry, 75, 46-53. doi:10.1016/j.genhosppsych.2022.01.008

Naeem F, Gul M, Irfan M, Munshi T, Asif A, Rashid S, et al. Brief Culturally adapted CBT (CaCBT) for depression: A randomized controlled trial from Pakistan. Journal of Affective Disorders. 2015;177:101-7.

Naeem F, Sarhandi I, Gul M, Khalid M, Aslam M, Anbrin A. A multicentre randomised controlled trial of a carer supervised culturally adapted cbt (cacbt) based self-help for depression in pakistan. Journal of Affective Disorders. 2013;156:224-7.

Nakagawa, A., Mitsuda, D., Sado, M., Abe, T., Fujisawa, D., Kikuchi, T., . . . Ono, Y. (2017). Effectiveness of supplementary cognitive-behavioral therapy for pharmacotherapy-resistant depression: A randomized controlled trial. Journal of clinical psychiatry, 78(8), 1126-1135

Nakimuli-Mpungu E, Wamala K, Okello J, Alderman S, Odokonyero R, Mojtabai R, et al. Group support psychotherapy for depression treatment in people with HIV/AIDS in northern Uganda: A single-centre randomised controlled trial. The Lancet HIV. 2015;2(5):e190-e9.

Nasrin, F., Rimes, K., Reinecke, A., Rinck, M., & Barnhofer, T. (2017). Effects of Brief Behavioural Activation on Approach and Avoidance Tendencies in Acute Depression: preliminary Findings. Behavioural and Cognitive Psychotherapy, 45(1), 58-72.

Neugebauer R, Kline J, Markowitz JC, Bleiberg KL, Baxi L, Rosing MA, et al. Pilot randomized controlled trial of interpersonal counseling for subsyndromal depression following miscarriage. Journal of Clinical Psychiatry. 2006;67(8):1299-304.

Newby JM, Lang T, Werner-Seidler A, Holmes E, Moulds ML. Alleviating distressing intrusive memories in depression: A comparison between computerised cognitive bias modification and cognitive behavioural education. Behaviour Research and Therapy. 2014;56:60-7.

Newby, J. M., et al. (2013). "Internet cognitive behavioural therapy for mixed anxiety and depression: a randomized controlled trial and evidence of effectiveness in primary care." Psychol Med 43(12): 2635-2648.

Newby, J., Robins, L., Wilhelm, K., Smith, J., Fletcher, T., Gillis, I., . . . Andrews, G. (2017). Web-Based Cognitive Behavior Therapy for Depression in People With Diabetes Mellitus: a Randomized Controlled Trial. Journal of medical Internet research, 19(5), e157.

Nezu AM, Perri MG. Social problem-solving therapy for unipolar depression: An initial dismantling investigation. Journal of Consulting and Clinical Psychology. 1989;57(3):408-13.

Nezu AM. Efficacy of a social problem-solving therapy approach for unipolar depression. Journal of Consulting and Clinical Psychology. 1986;54(2):196-202.

Ng SE, Tien A, Thayala JN, Ho RC, Chan MF. The effect of life story review on depression of older community-dwelling Chinese adults in Singapore: A preliminary result. International Journal of Geriatric Psychiatry. 2013;28(3):328-30.

Niedermoser DW, Kalak N, Kiyhankhadiv A, et al. Workplace-Related Interpersonal Group Psychotherapy to Improve Life at Work in Individuals With Major Depressive Disorders: a Randomized Interventional Pilot Study. Frontiers in psychiatry. 2020;11.

Nobis S, Lehr D, Ebert DD, Baumeister H, Snoek F, Riper H, et al. Efficacy of a web-based intervention with mobile phone support in treating depressive symptoms in adults with type 1 and type 2 diabetes: A randomized controlled trial. Diabetes care. 2015;38(5):776-83.

Noone, D., Payne, J., Stott, J., Aguirre, E., Patel-Palfreman, M. M., Stoner, C., . . . Spector, A. (2022). The Feasibility of a Mindfulness Intervention for Depression in People with Mild Dementia: A Pilot Randomized Controlled Trial. Clinical gerontologist, 1-13. doi:10.1080/07317115.2022.2094741

Northwood AK, Vukovich MM, Beckman A, et al. Intensive psychotherapy and case management for Karen refugees with major depression in primary care: a pragmatic randomized control trial. BMC Fam Pract. 2020;21(1):17.

Nyström, M., Stenling, A., Sjöström, E., Neely, G., Lindner, P., HassmÈn, P., . . . Carlbring, P. (2017). Behavioral activation versus physical activity via the internet: a randomized controlled trial. Journal of Affective Disorders, 215, 85-93.

O'Hara MW, Stuart S, Gorman LL, Wenzel A. Efficacy of interpersonal psychotherapy for postpartum depression. Archives of General Psychiatry. 2000;57(11):1039-45.

O'Mahen H, Himle JA, Fedock G, Henshaw E, Flynn H. A pilot randomized controlled trial of cognitive behavioral therapy for perinatal depression adapted for women with low incomes. Depression and anxiety. 2013;30(7):679-87.

O'Moore K, A., Newby, J. M., Andrews, G., Hunter, D. J., Bennell, K., Smith, J., & Williams, A. D. (2018). Internet Cognitive-Behavioral Therapy for Depression in Older Adults With Knee Osteoarthritis: A Randomized Controlled Trial. Arthritis Care Res (Hoboken), 70(1), 61-70. doi:10.1002/acr.23257

O'Neil A, Taylor B, Sanderson K, Cyril S, Chan B, Hawkes AL, et al. Efficacy and feasibility of a tele-health intervention for acute coronary syndrome patients with depression: Results of the "MoodCare" randomized controlled trial. Annals of Behavioral Medicine. 2014;48(2):163-74.

Oehler C, Görges F, Rogalla M, Rummel-Kluge C, Hegerl U. Efficacy of a Guided Web-Based Self-Management Intervention for Depression or Dysthymia: Randomized Controlled Trial With a 12-Month Follow-Up Using an Active Control Condition. J Med Internet Res. 2020;22(7):e15361.

Omidi A, Mohammadkhani P, Mohammadi A, Zargar F. Comparing mindfulness based cognitive therapy and traditional cognitive behavior therapy with treatments as usual on reduction of major depressive disorder symptoms. Iranian Red Crescent Medical Journal. 2013;15(2):142-6.

Onuigbo, L. N., Eseadi, C., Ebifa, S., Ugwu, U. C., Onyishi, C. N., & Oyeoku, E. K. (2019). Effect of rational emotive behavior therapy program on depressive symptoms among university students with blindness in Nigeria. Journal of Rational-Emotive & Cognitive-Behavior Therapy, 37(1), 17-38. doi:10.1007/s10942-018-0297-3

Onyechi, K. C. N., Eseadi, C., Okere, A. U., Onuigbo, L. N., Umoke, P. C. I., Anyaegbunam, N. J., . . . Ugorji, N. J. (2016). Effects of cognitive behavioral coaching on depressive symptoms in a sample of type 2 diabetic inpatients in Nigeria. Medicine (United States), 95(31).

Pace TM, Dixon DN. Changes in depressive self-schemata and depressive symptoms following cognitive therapy. Journal of Counseling Psychology. 1993;40(3):288.

Pecheur DR, Edwards KJ. A comparison of secular and religious versions of cognitive therapy with depressed Christian college students. Journal of Psychology and Theology. 1984.

Pellas, J., Renner, F., Ji, J. L., & Damberg, M. (2022). Telephone-based behavioral activation with mental imagery for depression: A pilot randomized clinical trial in isolated older adults during the Covid-19 pandemic. Int J Geriatr Psychiatry, 37(1). doi:10.1002/gps.5646

Penckofer SM, Ferrans C, Mumby P, Byrn M, Emanuele MA, Harrison PR, et al. A psychoeducational intervention (SWEEP) for depressed women with diabetes. Annals of Behavioral Medicine. 2012;44(2):192-206.

Perini S, Titov N, Andrews G. Clinician-assisted Internet-based treatment is effective for depression: Randomized controlled trial. Australian and New Zealand Journal of Psychiatry. 2009;43(6):571-8.

Petersen I, Hanass Hancock J, Bhana A, Govender K. A group-based counselling intervention for depression comorbid with HIV/AIDS using a task shifting approach in South Africa: A randomized controlled pilot study. Journal of Affective Disorders. 2014;158:78-84.

Pibernik-Okanović M, Hermanns N, Ajduković D, Kos J, Prašek M, Šekerija M, Lovrenčić MV. Does treatment of subsyndromal depression improve depression-related and diabetes-related outcomes? A randomised controlled comparison of psychoeducation, physical exercise and enhanced treatment as usual. Trials. 2015 Jul 15;16:305.

Piers, R. J., Farchione, T. J., Wong, B., Rosellini, A. J., & Cronin-Golomb, A. (2023). Telehealth Transdiagnostic Cognitive Behavioral Therapy for Depression in Parkinson's Disease: A Pilot Randomized Controlled Trial. Movement Disorders Clinical Practice, 10(1), 79-85. doi:10.1002/mdc3.13587

Pinniger, R., Brown, R. F., Thorsteinsson, E. B., & McKinley, P. (2012). Argentine tango dance compared to mindfulness meditation and a waiting-list control: A randomised trial for treating depression. Complement Ther Med, 20(6), 377-384. doi:10.1016/j.ctim.2012.07.003

Poleshuck EL, Gamble SA, Bellenger K, Lu N, Tu X, Sorensen S, et al. Randomized controlled trial of interpersonal psychotherapy versus enhanced treatment as usual for women with co-occurring depression and pelvic pain. Journal of psychosomatic research. 2014;77(4):264-72.

Pot AM, Bohlmeijer ET, Onrust S, Melenhorst A-S, Veerbeek M, De Vries W. The impact of life review on depression in older adults: A randomized controlled trial. International Psychogeriatrics. 2010;22(4):572-81.

Pots WT, Fledderus M, Meulenbeek PA, ten Klooster PM, Schreurs KM, Bohlmeijer ET. Acceptance and commitment therapy as a web-based intervention for depressive symptoms: Randomised controlled trial. The British Journal of Psychiatry. 2016;208(1):69-77.

Pots WTM, Meulenbeek PAM, Veehof MM, Klungers J, Bohlmeijer ET. The efficacy of mindfulness-based cognitive therapy as a public mental health intervention for adults with mild to moderate depressive symptomatology: A randomized controlled trial. PLoS One. 2014;9(10).

Pott, S. L., Kellett, S., Green, S., Daughters, S., & Delgadillo, J. (2022). Behavioral activation for depression delivered by drug and alcohol treatment workers: A pilot randomized controlled trial. J Subst Abuse Treat, 139, 108769. doi:10.1016/j.jsat.2022.108769

Power MJ, Freeman C. A randomized controlled trial of IPT versus CBT in primary care: With some cautionary notes about handling missing values in clinical trials. Clinical Psychology and Psychotherapy. 2012;19(2):159-69.

Prendergast J, Austin MP. Early childhood nurse-delivered cognitive behavioural counselling for post-natal depression. Australasian Psychiatry. 2001;9(3):255-9.

Preschl B, Maercker A, Wagner B, Forstmeier S, Banos RM, Alcaniz M, et al. Life-review therapy with computer supplements for depression in the elderly: A randomized controlled trial. Aging and Mental Health. 2012;16(8):964-74.

Propst LR, Ostrom R, Watkins P, Dean T, Mashburn D. Comparative efficacy of religious and nonreligious cognitive-behavioral therapy for the treatment of clinical depression in religious individuals. Journal of Consulting and Clinical Psychology. 1992;60(1):94-103.

Psaros, C., Stanton, A. M., Raggio, G. A., Mosery, N., Goodman, G. R., Briggs, E. S., . . . Safren, S. A. (2022). Optimizing PMTCT Adherence by Treating Depression in Perinatal Women with HIV in South Africa: A Pilot Randomized Controlled Trial. International journal of behavioral medicine. doi:10.1007/s12529-022-10071-z

Psarraki, E. E., Bacopoulou, F., Panagoulias, E., Michou, M., Pelekasis, P., Artemiadis, A., . . . Darviri, C. (2021). The effects of Pythagorean Self-Awareness Intervention on patients with major depressive disorder: A pilot randomized controlled trial. J Psychiatr Res, 138, 326-334. doi:10.1016/j.jpsychires.2021.03.067

Puckering C, McIntosh E, Hickey A, Longford J. Mellow Babies: A group intervention for infants and mothers experiencing postnatal depression. Counselling Psychology Review. 2010;25(1):28-38.

Pugh, N. E., Hadjistavropoulos, H. D., & Dirkse, D. (2016). A Randomised Controlled Trial of Therapist-Assisted, Internet-Delivered Cognitive Behavior Therapy for Women with Maternal Depression. PLoS ONE, 11(3), e0149186.

Qiu J, Chen W, Gao X, Xu Y, Tong H, Yang M, et al. A randomized controlled trial of group cognitive behavioral therapy for Chinese breast cancer patients with major depression. Journal of psychosomatic obstetrics and gynaecology. 2013;34(2):60-7.

Raevuori, A., Vahlberg, T., Korhonen, T., Hilgert, O., Aittakumpu-Hyden, R., & Forman-Hoffman, V. (2021). A therapist-guided smartphone app for major depression in young adults: A randomized clinical trial. J Affect Disord, 286, 228-238. doi:10.1016/j.jad.2021.02.007

Rahman A, Malik A, Sikander S, Roberts C, Creed F. Cognitive behaviour therapy-based intervention by community health workers for mothers with depression and their infants in rural Pakistan: A cluster-randomised controlled trial. Lancet. 2008;372(9642):902-9.

Raji Lahiji, M., Sajadian, A., Haghighat, S., Zarrati, M., Dareini, H., Raji Lahiji, M., & Razmpoosh, E. (2022). Effectiveness of logotherapy and nutrition counseling on psychological status, quality of life, and dietary intake among breast cancer survivors with depressive disorder: a randomized clinical trial. Supportive Care in Cancer. doi:10.1007/s00520-022-07237-6

Ransom D, Heckman TG, Anderson T, Garske J, Holroyd K, Basta T. Telephone-delivered, interpersonal psychotherapy for HIV-infected rural persons with depression: A pilot trial. Psychiatric Services. 2008;59(8):871-7.

Raue, P. J., Sirey, J. A., Dawson, A., Berman, J., & Bruce, M. L. (2019). Lay-delivered behavioral activation for depressed senior center clients: Pilot RCT. International journal of geriatric psychiatry, 34(11), 1715-1723. doi:10.1002/gps.5186

Raya-Tena, A., Fernández-San-Martin, M. I., Martin-Royo, J., Casañas, R., Sauch-Valmaña, G., Cols-Sagarra, C., . . . Jiménez-Herrera, M. F. (2021). Effectiveness of a Psychoeducational Group Intervention Carried Out by Nurses for Patients with

Depression and Physical Comorbidity in Primary Care: Randomized Clinical Trial. Int J Environ Res Public Health, 18(6). doi:10.3390/ijerph18062948

Rehm LP, Kornblith SJ, O'Hara MW, Lamparski DM, Romano JM, Volkin JI. An evaluation of major components in a self-control therapy program for depression. Behavior modification. 1981;5(4):459-89.

Reins, J. A., Boß, L., Lehr, D., Berking, M., & Ebert, D. D. (2019). The more I got, the less I need? Efficacy of Internet-based guided self-help compared to online psychoeducation for major depressive disorder. Journal of, 246, 695-705. doi:10.1016/j.jad.2018.12.065

Richards D, Timulak L, O'Brien E, Hayes C, Vigano N, Sharry J, et al. A randomized controlled trial of an internet-delivered treatment: Its potential as a low-intensity community intervention for adults with symptoms of depression. Behaviour Research and Therapy. 2015;75:20-31.

Richards SH, Dickens C, Anderson R, et al. Assessing the effectiveness of Enhanced Psychological Care for patients with depressive symptoms attending cardiac rehabilitation compared with treatment as usual (CADENCE): a pilot cluster randomised controlled trial. Trials 2018; 19(1).

Rief W, Bleichhardt G, Dannehl K, Euteneuer F, Wambach K. Comparing the Efficacy of CBASP with Two Versions of CBT for Depression in a Routine Care Center: a Randomized Clinical Trial. Psychotherapy and psychosomatics 2018;

Rohan KJ, Roecklein KA, Lindsey KT, Johnson LG, Lippy RD, Lacy TJ, et al. A randomized controlled trial of cognitive-behavioral therapy, light therapy, and their combination for seasonal affective disorder. Journal of Consulting and Clinical Psychology. 2007;75(3):489-500.

Rohde P, Stice E, Shaw H, Gau JM. Pilot trial of a dissonance-based cognitive-behavioral group depression prevention with college students. Behaviour Research and Therapy. 2016;82:21-7.

Rohde, P., Stice, E., Shaw, H., & Gau, J. M. (2014). Cognitive-behavioral group depression prevention compared to bibliotherapy and brochure control: Nonsignificant effects in pilot effectiveness trial with college students. Behaviour Research and Therapy, 55(1), 48-53.

Rohricht F, Papadopoulos N, Priebe S. An exploratory randomized controlled trial of body psychotherapy for patients with chronic depression. Journal of Affective Disorders. 2013;151(1):85-91.

Ross M, Scott M. An evaluation of the effectiveness of individual and group cognitive therapy in the treatment of depressed patients in an inner city health centre. The Journal of the Royal College of General Practitioners. 1985;35(274):239-42.

Rosso, I., Killgore, W., Olson, E., Webb, C., Fukunaga, R., Auerbach, R., . . . Rauch, S. (2017). Internet-based cognitive behavior therapy for major depressive disorder: a randomized controlled trial. Depress Anxiety, 34(3), 236-245.

Russell, A., Gaunt, D. M., Cooper, K., Barton, S., Horwood, J., Kessler, D., . . . et al. (2019). The feasibility of low-intensity psychological therapy for depression co-occurring with autism in adults: the Autism Depression Trial (ADEPT) â€“ a pilot randomised controlled trial. Autism. doi:10.1177/1362361319889272

Ruwaard J, Schrieken B, Schrijver M, Broeksteeg J, Dekker J, Vermeulen H, et al. Standardized web-based cognitive behavioural therapy of mild to moderate depression: A randomized controlled trial with a long-term follow-up. Cognitive Behaviour Therapy. 2009;38(4):206-21.

Sadler, P., McLaren, S., Klein, B., Harvey, J., & Jenkins, M. (2018). Cognitive behavior therapy for older adults with insomnia and depression: a randomized controlled trial in community mental health services. Sleep, 41(8) (no pagination). doi:10.1093/sleep/zsy105

Safren SA, Bedoya CA, O'Cleirigh C, Biello KB, Pinkston MM, Stein MD, et al. Cognitive behavioural therapy for adherence and depression in patients with HIV: A three-arm randomised controlled trial. The Lancet HIV. 2016;3(11):e529-e38.

Safren SA, Gonzalez JS, Wexler DJ, Psaros C, Delahanty LM, Blashill AJ, et al. A randomized controlled trial of cognitive behavioral therapy for adherence and depression (CBT-AD) in patients with uncontrolled type 2 diabetes. Diabetes care. 2014;37(3):625-33.

Safren SA, O'Cleirigh C, Tan JY, Raminani SR, Reilly LC, Otto MW, et al. A randomized controlled trial of cognitive behavioral therapy for adherence and depression (CBT-AD) in HIV-infected individuals. Health Psychology. 2009;28(1):1-10.

Safren, S. A., O'Cleirigh, C., Andersen, L. S., Magidson, J. F., Lee, J. S., Bainter, S. A., . . . Joska, J. A. (2021). Treating depression and improving adherence in HIV care with task-shared cognitive behavioural therapy in Khayelitsha, South Africa: a randomized controlled trial. J Int AIDS Soc, 24(10), e25823. doi:10.1002/jia2.25823

Saisanan Na Ayudhaya, W., Pityaratstian, N., & Jiamjarasrangsi, W. (2020). Effectiveness of Behavioral Activation in Treating Thai Older Adults with Subthreshold Depression Residing in the Community. Clin Interv Aging, 15, 2363-2374. doi:10.2147/cia.S274262

Salamanca-Sanabria A, Richards D, Timulak L, et al. A culturally adapted cognitive behavioral internet-delivered intervention for depressive symptoms: Randomized controlled trial. JMIR Mental Health. 2020;7(1).

Sander LB, Paganini S, Terhorst Y, et al. Effectiveness of a Guided Web-Based Self-help Intervention to Prevent Depression in Patients with Persistent Back Pain: The PROD-BP Randomized Clinical Trial. JAMA Psychiatry. 2020;77(10):1001-1011.

Savard J, Simard S, Giguere I, Ivers H, Morin CM, Maunsell E, et al. Randomized clinical trial on cognitive therapy for depression in women with metastatic breast cancer: Psychological and immunological effects. Palliative and Supportive Care. 2006;4(3):219-37.

Savari, Y., Mohagheghi, H., & Petrocchi, N. (2021). A preliminary investigation on the effectiveness of compassionate mind training for students with major depressive disorder: A randomized controlled trial. Mindfulness, 12(5), 1159-1172. doi:10.1007/s12671-020-01584-3

Scazufca, M., Nakamura, C. A., Seward, N., Moreno-Agostino, D., van de Ven, P., Hollingworth, W., . . . Araya, R. (2022). A task-shared, collaborative care psychosocial intervention for improving depressive symptomatology among older adults in a socioeconomically deprived area of Brazil (PROACTIVE): a pragmatic, two-arm, parallel-group, cluster-randomised controlled trial. Lancet Healthy Longev, 3(10), e690-e702. doi:10.1016/s2666-7568(22)00194-5

Schlicker S, Baumeister H, Buntrock C, et al. A web- And mobile-based intervention for comorbid, recurrent depression in patients with chronic back pain on sick leave (get.back): Pilot randomized controlled trial on feasibility, user satisfaction, and effectiveness. JMIR Mental Health. 2020;7(4).

Schmidt MM, Miller WR. Amount of therapist contact and outcome in a multidimensional depression treatment program. Acta Psychiatrica Scandinavica. 1983;67(5):319-32.

Schulberg HC, Block MR, Madonia MJ, Scott CP, Rodriguez E, Imber SD, et al. Treating major depression in primary care practice. Eight-month clinical outcomes. Archives of General Psychiatry. 1996;53(10):913-9.

Schuster, R., Leitner, I., Carlbring, P., & Laireiter, A.-R. (2017). Exploring blended group interventions for depression: randomised controlled feasibility study of a blended computer- and multimedia-supported psychoeducational group intervention for adults with depressive symptoms. Internet Interventions, 8, 63-71.

Scogin F, Hamblin D, Beutler L. Bibliotherapy for depressed older adults: A self-help alternative. The Gerontologist. 1987;27(3):383-7.

Scogin F, Jamison C, Gochneaur K. Comparative efficacy of cognitive and behavioral bibliotherapy for mildly and moderately depressed older adults. Journal of Consulting and Clinical Psychology. 1989;57(3):403-7.

Scogin F, Lichstein K, DiNapoli EA, et al. Effects of integrated telehealth-delivered cognitive-behavioral therapy for depression and insomnia in rural older adults. Journal of psychotherapy integration 2018; 28(3): 292-309.

Scott AI, Freeman CP. Edinburgh primary care depression study: Treatment outcome, patient satisfaction, and cost after 16 weeks. BMJ. 1992;304(6831):883-7.

Scott C, Tacchi MJ, Jones R, Scott J. Acute and one-year outcome of a randomised controlled trial of brief cognitive therapy for major depressive disorder in primary care. The British Journal of Psychiatry. 1997;171:131-4.

Scott MJ, Stradling SG. Group cognitive therapy for depression produces clinically significant reliable change in community-based settings. Behavioural Psychotherapy. 1990;18(01):1-19.

Segre LS, Brock RL, O'Hara MW. Depression treatment for impoverished mothers by point-of-care providers: A randomized controlled trial. Journal of Consulting and Clinical Psychology. 2015;83(2):314-24.

Selmi PM, Klein MH, Greist JH, Sorrell SP, Erdman HP. Computer-administered cognitive-behavioral therapy for depression. American Journal of Psychiatry. 1990;147(1):51-6.

Serfaty MA, Haworth D, Blanchard M, Buszewicz M, Murad S, King M. Clinical effectiveness of individual cognitive behavioral therapy for depressed older people in primary care: A randomized controlled trial. Archives of General Psychiatry. 2009;66(12):1332-40.

Serfaty, M., King, M., Nazareth, I., Moorey, S., Aspden, T., Tookman, A., . . . Jones, L. (2019). Manualised cognitive and behavioural therapy in treating depression in advanced cancer: the CanTalk RCT. Health Technology Assessment, 23(19), 1-106. doi:10.3310/hta23190

Serrano JP, Latorre JM, Gatz M, Montanes J. Life review therapy using autobiographical retrieval practice for older adults with depressive symptomatology. Psychology and Aging. 2004;19(2):270-7.

Serrano Selva JP, Latorre Postigo JM, Ros Segura L, Navarro Bravo B, Aguilar Corcoles MJ, Nieto Lopez M, et al. Life review therapy using autobiographical retrieval practice for older adults with clinical depression. Psicothema. 2012;24(2):224-9.

Sheeber LB, Seeley JR, Feil EG, Davis B, Sorensen E, Kosty DB, et al. Development and pilot evaluation of an Internet-facilitated cognitive-behavioral intervention for maternal depression. Journal of Consulting and Clinical Psychology. 2012;80(5):739-49.

Sheeber, L., Feil, E., Seeley, J., Leve, C., Gau, J., Davis, B., . . . Allan, S. (2017). Mom-net: evaluation of an internet-facilitated cognitive behavioral intervention for low-income depressed mothers. Journal of Consulting and Clinical Psychology, 85(4), 355-366.

Shih, V. W. Y., Chan, W. C., Tai, O. K., Wong, H. L., Cheng, C. P. W., & Wong, C. S. M. (2021). Mindfulness-Based Cognitive Therapy for Late-Life Depression: a Randomised Controlled Trial. East Asian Arch Psychiatry, 31(2), 27-35. doi:10.12809/eaap2075

Simoni JM, Wiebe JS, Sauceda JA, Huh D, Sanchez G, Longoria V, et al. A preliminary RCT of CBT-AD for adherence and depression among HIV-positive Latinos on the U.S.-Mexico border: The Nuevo Dia study. AIDS and behavior. 2013;17(8):2816-29.

Simpson S, Corney R, Beecham J. A randomized controlled trial to evaluate the effectiveness and cost-effectiveness of psychodynamic counselling for general practice patients with chronic depression. Psychological Medicine. 2003;33(2):229-39.

Simson U, Nawarotzky U, Friese G, Porck W, Schottenfeld-Naor Y, Hahn S, et al. Psychotherapy intervention to reduce depressive symptoms in patients with diabetic foot syndrome. Diabetic Medicine. 2008;25(2):206-12.

Sinniah, A., Oei, T., Maniam, T., & Subramaniam, P. (2017). Positive effects of Individual Cognitive Behavior Therapy for patients with unipolar mood disorders with suicidal ideation in Malaysia: a randomised controlled trial. Psychiatry research, 254, 179-189.

Smith, J., Newby, J. M., Burston, N., Murphy, M. J., Michael, S., Mackenzie, A., . . . Andrews, G. (2017). Help from home for depression: A randomised controlled trial comparing internet-delivered cognitive behaviour therapy with bibliotherapy for depression. Internet Interventions, 9, 25-37.

Spek V, Nyklicek I, Smits N, Cuijpers P, Riper H, Keyzer J, et al. Internet-based cognitive behavioural therapy for subthreshold depression in people over 50 years old: A randomized controlled clinical trial. Psychological Medicine. 2007;37(12):1797-806.

Spruill, T. M., Friedman, D., Diaz, L., Butler, M. J., Goldfeld, K. S., O'Kula, S., . . . Devinsky, O. (2021). Telephone-based depression self-management in Hispanic adults with epilepsy: a pilot randomized controlled trial. Transl Behav Med, 11(7), 1451-1460. doi:10.1093/tbm/ibab045

Sreevani R, Reddemma K, Chan CL, Leung PP, Wong V, Chan CH. Effectiveness of integrated body-mind-spirit group intervention on the well-being of Indian patients with depression: A pilot study. Journal of Nursing Research. 2013;21(3):179-86.

Stiles-Shields, C., Montague, E., Kwasny, M. J., & Mohr, D. C. (2019). Behavioral and cognitive intervention strategies delivered via coached apps for depression: Pilot trial. Psychological services, 16(2), 233-238. doi:10.1037/ser0000261

Strauss C, Hayward M, Chadwick P. Group person-based cognitive therapy for chronic depression: A pilot randomized controlled trial. British Journal of Clinical Psychology. 2012;51(3):345-50.

Stuart, R., Fischer, H., Leitzke, A. S., Becker, D., Saheba, N., & Coleman, K. J. (2022). The Effectiveness of Internet-Based Cognitive Behavioral Therapy for the Treatment of Depression in a Large Real-World Primary Care Practice: A Randomized Trial. The Permanente journal, 1-8. doi:10.7812/TPP/21.183

Sugg HVR, Richards DA, Frost J. Morita Therapy for depression (Morita Trial): a pilot randomised controlled trial. BMJ open 2018; 8(8).

Sun, Q., Xu, H., Zhang, W., Zhou, Y., & Lv, Y. (2022). Behavioral Activation Therapy for Subthreshold Depression in Stroke Patients: An Exploratory Randomized Controlled Trial. Neuropsychiatric Disease and Treatment, 18, 2795-2805. doi:10.2147/NDT.S392403

Swartz HA, Frank E, Zuckoff A, Cyranowski JM, Houck PR, Cheng Y, et al. Brief interpersonal psychotherapy for depressed mothers whose children are receiving psychiatric treatment. American Journal of Psychiatry. 2008;165(9):1155-62.

Szumska I, Gola M, Rusanowska M, et al. Mindfulness-based cognitive therapy reduces clinical symptoms, but do not change frontal alpha asymmetry in people with major depression disorder. International Journal of Neuroscience. 2020.

Takagaki K, Okamoto Y, Jinnin R, Mori A, Nishiyama Y, Yamamura T, et al. Behavioral activation for late adolescents with subthreshold depression: A randomized controlled trial. European Child and Adolescent Psychiatry. 2016;25(11):1171-82.

Talbot NL, Chaudron LH, Ward EA, Duberstein PR, Conwell Y, O'Hara MW, et al. A randomized effectiveness trial of interpersonal psychotherapy for depressed women with sexual abuse histories. Psychiatric Services. 2011;62(4):374-80.

Taylor CB, Conrad A, Wilhelm FH, Strachowski D, Khaylis A, Neri E, et al. Does improving mood in depressed patients alter factors that may affect cardiovascular disease risk? Journal of Psychiatric Research. 2009;43(16):1246-52.

Taylor FG, Marshall WL. Experimental analysis of a cognitive-behavioral therapy for depression. Cognitive Therapy and Research. 1977;1(1):59-72.

Teichman Y, Bar-el Z, Shor H, Sirota P, Elizur A. A comparison of two modalities of cognitive therapy (individual and marital) in treating depression. Psychiatry. 1995;58(2):136-48.

Thomas, S. A., Drummond, A. E., Lincoln, N. B., Palmer, R. L., das Nair, R., Latimer, N. R., . . . Topcu, G. (2019). Behavioural activation therapy for post-stroke depression: the BEADS feasibility RCT. Health Technol Assess, 23(47), 1-176. doi:10.3310/hta23470

Titov N, Andrews G, Davies M, McIntyre K, Robinson E, Solley K. Internet treatment for depression: A randomized controlled trial comparing clinician vs. technician assistance. PLoS One. 2010;5(6):e10939.

Titov N, Dear BF, Ali S, Zou JB, Lorian CN, Johnston L, et al. Clinical and cost-effectiveness of therapist-guided internet-delivered cognitive behavior therapy for older adults with symptoms of depression: A randomized controlled trial. Behavior Therapy. 2015;46(2):193-205.

Tobin, K., Davey-Rothwell, M. A., Nonyane, B. A. S., Knowlton, A., Wissow, L., & Latkin, C. A. (2017). RCT of an integrated CBT-HIV intervention on depressive symptoms and HIV risk.

Tomasino, K., Lattie, E., Ho, J., Palac, H., Kaiser, S., & Mohr, D. (2017). Harnessing Peer Support in an Online Intervention for Older Adults with Depression. American Journal of Geriatric Psychiatry, 25(10), 1109-1119.

Tong, P., Bu, P., Yang, Y., Dong, L., Sun, T., & Shi, Y. (2019). Group cognitive behavioural therapy can reduce stigma and improve treatment compliance in major depressive disorder patients. Early intervention in psychiatry. doi:10.1111/eip.12841

Tovote KA, Fleer J, Snippe E, Peeters A, Emmelkamp PMG, Sanderman R, et al. Individual mindfulness-based cognitive therapy and cognitive behavior therapy for treating depressive symptoms in patients with diabetes: Results of a randomized controlled trial. Diabetes care. 2014;37(9):2427-34.

Tulbure BT, Andersson G, Salagean N, Pearce M, Koenig HG. Religious versus Conventional Internet-based Cognitive Behavioral Therapy for Depression. J Relig Health 2018; 57(5): 1634-48.

Turner A, Hambridge J, Baker A, Bowman J, McElduff P. Randomised controlled trial of group cognitive behaviour therapy versus brief intervention for depression in cardiac patients. Australian and New Zealand Journal of Psychiatry. 2013;47(3):235-43.

Tyson GM, Range LM. Gestalt dialogues as a treatment for mild depression: Time works just as well. Journal of clinical psychology. 1987;43(2):227-31.

van der Zanden, R., Kramer, J., Gerrits, R., & Cuijpers, P. (2012). Effectiveness of an online group course for depression in adolescents and young adults: a randomized trial. Journal of medical Internet research, 14(3), e86.

Van Horne, B. S., Nong, Y. H., Cain, C. M., Sampson, M., Greeley, C. S., & Puryear, L. (2022). A promising new model of care for postpartum depression: A randomised controlled trial of a brief home visitation program conducted in Houston, Texas, USA. Health Soc Care Community, 30(5), e2203-e2213. doi:10.1111/hsc.13658

Van Lieshout, R. J., Layton, H., Savoy, C. D., Brown, J. S. L., Ferro, M. A., Streiner, D. L., . . . Hanna, S. (2021). Effect of Online 1-Day Cognitive Behavioral Therapy-Based Workshops Plus Usual Care vs Usual Care Alone for Postpartum Depression: A Randomized Clinical Trial. JAMA Psychiatry, 78(11), 1200-1207. doi:10.1001/jamapsychiatry.2021.2488

Van Lieshout, R. J., Layton, H., Savoy, C. D., Haber, E., Feller, A., Biscaro, A., . . . Ferro, M. A. (2022). Public Health Nurse-delivered Group Cognitive Behavioural Therapy for Postpartum Depression: A Randomized Controlled Trial. Can J Psychiatry, 67(6), 432-440. doi:10.1177/07067437221074426

van Luenen, S., Garnefski, N., Spinhoven, P., & Kraaij, V. (2018). Guided internet-based intervention for people with HIV and depressive symptoms: a randomised controlled trial in the Netherlands. The lancet HIV, 5(9), e488-e497.

van Schaik A, van Marwijk H, Adèr H, van Dyck R, de Haan M, Penninx B, et al. Interpersonal psychotherapy for elderly patients in primary care. The American Journal of Geriatric Psychiatry. 2006;14(9):777-86.

Vázquez FL, López L, Torres Á J, et al. Analysis of the Components of a Cognitive-Behavioral Intervention for the prevention of Depression Administered via Conference Call to Nonprofessional Caregivers: A Randomized Controlled Trial. Int J Environ Res Public Health. 2020;17(6).

Vázquez González FL, Otero Otero P, Torres Iglesias A, Hermida Garcìa E, Blanco Seoane V, Dìaz Fernández O. A brief problem-solving indicated-prevention intervention for prevention of depression in nonprofessional caregivers. Psicothema. 2013;25(1):87-92.

Vázquez, F. L., Torres, Á., Otero, P., Blanco, V., Díaz, O., & Estévez, L. E. (2017). Analysis of the components of a cognitive-behavioral intervention administered via conference call for preventing depression among non-professional caregivers: A pilot study. Aging Ment Health, 21(9), 938-946.

Verduyn C, Barrowclough C, Roberts J, Tarrier N, Harrington R. Maternal depression and child behaviour problems: Randomised placebo-controlled trial of a cognitive-behavioural group intervention. British Journal of Psychiatry. 2003;183(OCT.):342-8.

Vernmark K, Lenndin J, Bjärehed J, Carlsson M, Karlsson J, Oberg J, et al. Internet administered guided self-help versus individualized e-mail therapy: A randomized trial of two versions of CBT for major depression. Behaviour Research and Therapy. 2010;48(5):368-76.

Victor-Aigbodion, V., Eseadi, C., Ardi, Z., Sewagegn, A. A., Ololo, K., Abonor, L. B., . . . Effanga, O. A. (2023). Effectiveness of rational emotive behavior therapy in reducing depression among undergraduate medical students. Medicine (Baltimore), 102(4), e32724. doi:10.1097/md.0000000000032724

Vigod, S. N., Slyfield Cook, G., Macdonald, K., Hussain-Shamsy, N., Brown, H. K., de Oliveira, C., . . . Dennis, C.-L. (2021). Mother matters: Pilot randomized wait-list controlled trial of an online therapist-facilitated discussion board and support group for postpartum depression symptoms. Depress Anxiety, 38(8), 816-825. doi:10.1002/da.23163

Vitriol VG, Ballesteros ST, Florenzano RU, Weil KP, Benadof DF. Evaluation of an outpatient intervention for women with severe depression and a history of childhood trauma. Psychiatric Services. 2009;60(7):936-42.

Warmerdam L, Straten A, Twisk J, Riper H, Cuijpers P. Internet-based treatment for adults with depressive symptoms: Randomized controlled trial. Journal of Medical Internet Research. 2008;10(4):e44.

Watkins ER, Taylor RS, Byng R, Baeyens C, Read R, Pearson K, et al. Guided self-help concreteness training as an intervention for major depression in primary care: A Phase II randomized controlled trial. Psychological Medicine. 2012;42(7):1359-71.

Watt LM, Cappeliez P. Integrative and instrumental reminiscence therapies for depression in older adults: Intervention strategies and treatment effectiveness. Aging and Mental Health. 2000;4(2):166-77.

Westerhof, G. J., Lamers, S. M. A., Postel, M. G., & Bohlmeijer, E. T. (2019). Online Therapy for Depressive Symptoms: An Evaluation of Counselor-Led and Peer-Supported Life Review Therapy. The Gerontologist, 59(1), 135-146. doi:10.1093/geront/gnx140

Wiersma JE, Schaik DJF, Hoogendorn AW, Dekker JJ, Van HL, Schoevers RA, et al. The effectiveness of the cognitive behavioral analysis system of psychotherapy for chronic depression: A randomized controlled trial. Psychotherapy and Psychosomatics. 2014;83(5):263-9.

Williams AD, Blackwell SE, Mackenzie A, Holmes EA, Andrews G. Combining imagination and reason in the treatment of depression: A randomized controlled trial of internet-based cognitive-bias modification and internet-CBT for depression. Journal of Consulting and Clinical Psychology. 2013;81(5):793-9.

Williams C, McClay CA, Matthews L, et al. Community-based group guided self-help intervention for low mood and stress: Randomised controlled trial. British Journal of Psychiatry 2018; 212(2): 88-95.

Williams C, Wilson P, Morrison J, McMahon A, Andrew W, Allan L, et al. Guided self-help cognitive behavioural therapy for depression in primary care: A randomised controlled trial. PLoS One. 2013;8(1):e52735.

Wilson PH, Goldin JC, Charbonneau-Powis M. Comparative efficacy of behavioral and cognitive treatments of depression. Cognitive Therapy and Research. 1983;7(2):111-24.

Winnebeck, E., Fissler, M., Gärtner, M., Chadwick, P., & Barnhofer, T. (2017). Brief training in mindfulness meditation reduces symptoms in patients with a chronic or recurrent lifetime history of depression: A randomized controlled study. Behaviour Research and Therapy, 99, 124-130. doi:10.1016/j.brat.2017.10.005

Wollersheim JP, Wilson GL. Group treatment of unipolar depression: A comparison of coping, supportive, bibliotherapy, and delayed treatment groups. Professional Psychology: Research and Practice. 1991;22(6):496.

Wong DF. Cognitive and health-related outcomes of group cognitive behavioural treatment for people with depressive symptoms in Hong Kong: Randomized wait-list control study. Australian and New Zealand Journal of Psychiatry. 2008;42(8):702-11.

Wong DF. Cognitive behavioral treatment groups for people with chronic depression in Hong Kong: A randomized wait-list control design. Depression and anxiety. 2008;25(2):142-8.

Wong SYS, Sun YY, Chan ATY, et al. Treating Subthreshold Depression in Primary Care: a Randomized Controlled Trial of Behavioral Activation With Mindfulness. Annals of family medicine 2018; 16(2): 111-9.

Wright, J. H., Owen, J., Eells, T. D., Antle, B., Bishop, L. B., Girdler, R., . . . Ali, S. (2022). Effect of Computer-Assisted Cognitive Behavior Therapy vs Usual Care on Depression Among Adults in Primary Care: A Randomized Clinical Trial. JAMA Netw Open, 5(2), e2146716. doi:10.1001/jamanetworkopen.2021.46716

Wuthrich VM, Rapee RM, Kangas M, Perini S. Randomized controlled trial of group cognitive behavioral therapy compared to a discussion group for co-morbid anxiety and depression in older adults. Psychological Medicine. 2016;46(4):785-95.

Wuthrich VM, Rapee RM. Randomised controlled trial of group cognitive behavioural therapy for comorbid anxiety and depression in older adults. Behaviour Research and Therapy. 2013;51(12):779-86.

Xie, J., He, G., Ding, S., Pan, C., Zhang, X., Zhou, J., & Iennaco, J. D. (2019). A randomized study on the effect of modified behavioral activation treatment for depressive symptoms in rural left-behind elderly. Psychotherapy research : journal of the Society for Psychotherapy Research, 29(3), 372-382. doi:10.1080/10503307.2017.1364444

Yang X, Zhao J, Chen Y, Zu S, Zhao J. Comprehensive self-control training benefits depressed college students: a six-month randomized controlled intervention trial. Journal of affective disorders 2018; 226: 251-60.

Ying, Y., Ji, Y., Kong, F., Wang, M., Chen, Q., Wang, L., . . . Ruan, L. (2022). Efficacy of an internet-based cognitive behavioral therapy for subthreshold depression among Chinese adults: a randomized controlled trial. Psychological medicine, 1-11. doi:10.1017/S0033291722000599

Yuan J, Yin Y, Tang X, et al. Culturally adapted and lay-delivered cognitive behaviour therapy for older adults with depressive symptoms in rural China: a pilot trial. Behavioural and cognitive psychotherapy. 2020:1-5.

Zemestani M, Davoodi I, Honarmand MM, Zargar Y, Ottaviani C. Comparative effects of group metacognitive therapy versus behavioural activation in moderately depressed students. Journal of Mental Health. 2016;25(6):479-85.

Zemestani M, Mozaffari S. Acceptance and commitment therapy for the treatment of depression in persons with physical disability: a randomized controlled trial. Clin Rehabil. 2020;34(7):938-947.

Zemestani, M., & Fazeli Nikoo, Z. (2020). Effectiveness of mindfulness-based cognitive therapy for comorbid depression and anxiety in pregnancy: a randomized controlled trial. Archives of Women's Mental Health. doi:10.1007/s00737-019-00962-8

Zhao, Y., Munro-Kramer, M. L., Shi, S., Wang, J., & Zhao, Q. (2019). Effects of antenatal depression screening and intervention among Chinese high-risk pregnant women with medically defined complications: A randomized controlled trial. Early intervention in psychiatry, 13(5), 1090-1098. doi:10.1111/eip.12731

Zu S, Xiang Y-T, Liu J, Zhang L, Wang G, Ma X, et al. A comparison of cognitive-behavioral therapy, antidepressants, their combination and standard treatment for Chinese patients with moderate–severe major depressive disorders. Journal of Affective Disorders. 2014;152-154:262-7.

Psychotherapy versus Antidepressive Medication

Altamura, M., Iuso, S., Terrone, G., Balzotti, A., Carnevale, R., Malerba, S., . . . Petito, A. (2017). Comparing interpersonal counseling and antidepressant treatment in primary care patients with anxious and nonanxious major depression disorder: A randomized control trial. Clinical Neuropsychiatry, 14(4), 257-262.

Bastos AG, Guimaraes LSP, Trentini CM. The efficacy of long-term psychodynamic psychotherapy, fluoxetine and their combination in the outpatient treatment of depression. Psychotherapy Research. 2015;25(5):612-624.

Bedi N, Chilvers C, Churchill R, Dewey M, Duggan C, Fielding K, et al. Assessing effectiveness of treatment of depression in primary care. Partially randomised preference trial. The British Journal of Psychiatry. 2000;177:312-8.

Blackburn IM, Moore RG. Controlled acute and follow-up trial of cognitive therapy and pharmacotherapy in out-patients with recurrent depression. The British Journal of Psychiatry. 1997;171:328-34.

Blom MB, Jonker K, Dusseldorp E, Spinhoven P, Hoencamp E, Haffmans J, et al. Combination treatment for acute depression is superior only when psychotherapy is added to medication. Psychotherapy and Psychosomatics. 2007;76(5):289-97.

Browne G, Steiner M, Roberts J, Gafni A, Byrne C, Dunn E, et al. Sertraline and/or interpersonal psychotherapy for patients with dysthymic disorder in primary care: 6-month comparison with longitudinal 2-year follow-up of effectiveness and costs. Journal of Affective Disorders. 2002;68(2-3):317-30.

Chibanda D, Shetty AK, Tshimanga M, Woelk G, Stranix-Chibanda L, Rusakaniko S. Group problem-solving therapy for postnatal depression among HIV-positive and HIV-negative mothers in Zimbabwe. Journal of the International Association of Providers of AIDS Care. 2014;13(4):335-41.

David D, Szentagotai A, Lupu V, Cosman D. Rational emotive behavior therapy, cognitive therapy, and medication in the treatment of major depressive disorder: A randomized clinical trial, posttreatment outcomes, and six-month follow-up. Journal of clinical psychology. 2008;64(6):728-46.

Dekker JJ, Koelen JA, Van HL, Schoevers RA, Peen J, Hendriksen M, et al. Speed of action: The relative efficacy of short psychodynamic supportive psychotherapy and pharmacotherapy in the first 8 weeks of a treatment algorithm for depression. Journal of Affective Disorders. 2008;109(1-2):183-8.

Dimidjian S, Hollon SD, Dobson KS, Schmaling KB, Kohlenberg RJ, Addis ME, et al. Randomized trial of behavioral activation, cognitive therapy, and antidepressant medication in the acute treatment of adults with major depression. Journal of Consulting and Clinical Psychology. 2006;74(4):658-70.

Dunlop, B. W., Kelley, M. E., Aponte-Rivera, V., Mletzko-Crowe, T., Kinkead, B., Ritchie, J. C., . . . Mayberg, H. S. (2017). Effects of patient preferences on outcomes in the Predictors of Remission in Depression to Individual and Combined Treatments (PReDICT) study. Am J Psychiatry, 174(6), 546-556.

Dunn RJ. Cognitive modification with depression-prone psychiatric patients. Cognitive Therapy and Research. 1979;3(3):307-17.

Dunner DL, Schmaling KB, Hendrickson H, Becker J, Lehman A, Bea C. Cognitive therapy versus fluoxetine in the treatment of dysthymic disorder. Depression. 1996;4(1):34-41.

Elkin I, Shea MT, Watkins JT, Imber SD, Sotsky SM, Collins JF, et al. National institute of mental health treatment of depression collaborative research program. General effectiveness of treatments. Archives of General Psychiatry. 1989;46(11):971-82; discussion 83.

Faramarzi M, Alipor A, Esmaelzadeh S, Kheirkhah F, Poladi K, Pash H. Treatment of depression and anxiety in infertile women: Cognitive behavioral therapy versus fluoxetine. Journal of Affective Disorders. 2008;108(1-2):159-64.

Finkenzeller W, Zobel I, Rietz S, Schramm E, Berger M. [Interpersonal psychotherapy and pharmacotherapy for post-stroke depression. Feasibility and effectiveness]. Der Nervenarzt. 2009;80(7):805-12.

Gater R, Waheed W, Husain N, Tomenson B, Aseem S, Creed F. Social intervention for British Pakistani women with depression: Randomised controlled trial. The British Journal of Psychiatry. 2010;197(3):227-33.

Gilliam, F. G., Black, K. J., Carter, J., Freedland, K. E., Sheline, Y. I., Tsai, W. Y., & Lustman, P. J. (2019). A Trial of Sertraline or Cognitive Behavior Therapy for Depression in Epilepsy. Annals of Neurology, 86(4), 552-560. doi:10.1002/ana.25561

Hautzinger M, De Jong-Meyer R, Treiber R, Rudolf GAE, Thien U. Efficacy of cognitive behavior therapy, pharmacotherapy, and the combination of both in non-melancholic, unipolar depression. Zeitschrift fur Klinische Psychologie. 1996;25(2):130-45.

Hollon SD, DeRubeis RJ, Evans MD, Wiemer MJ, Garvey MJ, Grove WM, et al. Cognitive therapy and pharmacotherapy for depression. Singly and in combination. Archives of General Psychiatry. 1992;49(10):774-81.

Husain N, Chaudhry N, Fatima B, Husain M, Rizwana A, Chaudhry IB, et al. Antidepressant and group psychosocial treatment for depression: A rater blind exploratory RCT from a low income country. Behavioural and Cognitive Psychotherapy. 2014;42(6):693-705.

Jarrett RB, Schaffer M, McIntire D, Witt-Browder A, Kraft D, Risser RC. Treatment of atypical depression with cognitive therapy or phenelzine: A double-blind, placebo-controlled trial. Archives of General Psychiatry. 1999;56(5):431-7.

Keller MB, McCullough JP, Klein DN, Arnow B, Dunner DL, Gelenberg AJ, et al. A comparison of nefazodone, the cognitive behavioral-analysis system of psychotherapy, and their combination for the treatment of chronic depression. The New England journal of medicine. 2000;342(20):1462-70.

Kennedy SH, Konarski JZ, Segal ZV, Lau MA, Bieling PJ, McIntyre RS, et al. Differences in brain glucose metabolism between responders to CBT and venlafaxine in a 16-week randomized controlled trial. American Journal of Psychiatry. 2007;164(5):778-88.

Maldonado López A. Terapia de conducta y depresión: Un análisis experimental de los modelos conductual y cognitivo. Revista de Psicologia General y Aplicada. 1982.

Markowitz JC, Kocsis JH, Bleiberg KL, Christos PJ, Sacks M. A comparative trial of psychotherapy and pharmacotherapy for "pure" dysthymic patients. Journal of Affective Disorders. 2005;89(1-3):167-75.

Marshall MB, Zuroff DC, McBride C, Bagby RM. Self-criticism predicts differential response to treatment for major depression. Journal of clinical psychology. 2008;64(3):231-44.

Martin SD, Martin E, Rai SS, Richardson MA, Royall R. Brain blood flow changes in depressed patients treated with interpersonal psychotherapy or venlafaxine hydrochloride: Preliminary findings. Archives of General Psychiatry. 2001;58(7):641-8.

McKnight DL, Nelson-Gray RO, Barnhill J. Dexamethasone suppression test and response to cognitive therapy and antidepressant medication. Behavior Therapy. 1992;23(1):99-111.

McLean PD, Hakstian AR. Clinical depression: Comparative efficacy of outpatient treatments. Journal of Consulting and Clinical Psychology. 1979;47(5):818-36.

Mehrotra, R., Cukor, D., Unruh, M., Rue, T., Heagerty, P., Cohen, S. D., . . . Hedayati, S. S. (2019). Comparative Efficacy of Therapies for Treatment of Depression for Patients Undergoing Maintenance Hemodialysis: A Randomized Clinical Trial. Annals of internal medicine, 170(6), 369-379. doi:10.7326/M18-2229

Milgrom J, Gemmill AW, Ericksen J, Burrows G, Buist A, Reece J. Treatment of postnatal depression with cognitive behavioural therapy, sertraline and combination therapy: A randomised controlled trial. Australian and New Zealand Journal of Psychiatry. 2015;49(3):236-45.

Miranda J, Chung JY, Green BL, Krupnick J, Siddique J, Revicki DA, et al. Treating depression in predominantly low-income young minority women: A randomized controlled trial. Jama. 2003;290(1):57-65.

Mohr DC, Boudewyn AC, Goodkin DE, Bostrom A, Epstein L. Comparative outcomes for individual cognitive-behavior therapy, supportive-expressive group psychotherapy, and sertraline for the treatment of depression in multiple sclerosis. Journal of Consulting and Clinical Psychology. 2001;69(6):942-9.

Murphy GE, Simons AD, Wetzel RD, Lustman PJ. Cognitive therapy and pharmacotherapy. Singly and together in the treatment of depression. Archives of General Psychiatry. 1984;41(1):33-41.

Murphy GE, Carney RM, Knesevich MA, Wetzel RD, Whitworth P. Cognitive behavior therapy, relaxation training, and tricyclic antidepressant medication in the treatment of depression. Psychological reports. 1995;77(2):403-20.

Mynors-Wallis L, Gath D, Lloyd-Thomas A, Tomlinson D. Randomised controlled trial comparing problem solving treatment with amitriptyline and placebo for major depression in primary care. BMJ. 1995;310(6977):441-5.

Mynors-Wallis LM, Gath DH, Day A, Baker F. Randomised controlled trial of problem solving treatment, antidepressant medication, and combined treatment for major depression in primary care. BMJ. 2000;320(7226):26-30.

Parker G, Blanch B, Paterson A, Hadzi-Pavlovic D, Sheppard E, Manicavasagar V, et al. The superiority of antidepressant medication to cognitive behavior therapy in melancholic depressed patients: A 12-week single-blind randomized study. Acta Psychiatrica Scandinavica. 2013;128(4):271-81.

Quilty LC, Dozois DJA, Lobo DSS, Ravindran LN, Bagby RM. Cognitive structure and processing during cognitive behavioral therapy vs. pharmacotherapy for depression. International Journal of Cognitive Therapy. 2014;7(3):235-50.

Rush AJ, Beck AT, Kovacs M, Hollon S. Comparative efficacy of cognitive therapy and pharmacotherapy in the treatment of depressed outpatients. Cognitive Therapy and Research. 1977;1(1):17-37.

Schramm E, Zobel I, Schoepf D, Fangmeier T, Schnell K, Walter H, et al. Cognitive behavioral analysis system of psychotherapy versus escitalopram in chronic major depression. Psychotherapy and Psychosomatics. 2015;84(4):227-40.

Schulberg HC, Block MR, Madonia MJ, Scott CP, Rodriguez E, Imber SD, et al. Treating major depression in primary care practice. Eight-month clinical outcomes. Archives of General Psychiatry. 1996;53(10):913-9.

Scott AI, Freeman CP. Edinburgh primary care depression study: Treatment outcome, patient satisfaction, and cost after 16 weeks. BMJ. 1992;304(6831):883-7.

Shamsaei F, Rahimi A, Zarabian MK, Sedehi M. Efficacy of pharmacotherapy and cognitive therapy, alone and in combination in major depressive disorder. Hong Kong Journal of Psychiatry. 2008;18(2):76-80.

Sharp DJ, Chew-Graham C, Tylee A, Lewis G, Howard L, Anderson I, et al. A pragmatic randomised controlled trial to compare antidepressants with a community-based psychosocial intervention for the treatment of women with postnatal depression: the RESPOND trial. Health Technology Assessment. 2010;14(43):1-153.

Thompson LW, Coon DW, Gallagher-Thompson D, Sommer BR, Koin D. Comparison of desipramine and cognitive/behavioral therapy in the treatment of elderly outpatients with mild-to-moderate depression. The American Journal of Geriatric Psychiatry. 2001;9(3):225-40.

Zu S, Xiang Y-T, Liu J, Zhang L, Wang G, Ma X, et al. A comparison of cognitive-behavioral therapy, antidepressants, their combination and standard treatment for Chinese patients with moderate–severe major depressive disorders. Journal of Affective Disorders. 2014;152-154:262-7.

Basirat, Z., Kheirkhah, F., Faramarzi, M., Esmaelzadeh, S., Khafri, S., & Tajali, Z. (2022). Pharmacotherapy or Psychotherapy? Selective Treatment Depression in The Infertile Women with Recurrent Pregnancy Loss: A Triple-Arm Randomized Controlled Trial. International Journal of Fertility and Sterility, 16(3), 211-219. doi:10.22074/ijfs.2021.529258.1124
