# Supplementary material for: Standardized effect sizes are far from “Standardized”: A primer and empirical illustration in depression psychotherapy meta-analyses
Source: PLOS Ment Health. 2025 Jul 1;2(7):e0000347. doi: 10.1371/journal.pmen.0000347 (PMC12798590; doi:10.1371/journal.pmen.0000347)
Supplement: S1 Table — SMDEP/EP = SMD calculated by dividing the mean endpoint difference by the pooled endpoint SD; SMDCS/BL = SMD calculated by dividing the mean change score difference by the pooled baseline SD; SMDCS/CS = SMD calculated by dividing the mean change score difference by the pooled change score SD; SMDCS/EP = SMD calculated by dividing the mean change score difference by the pooled endpoint SD. (PDF) [file pmen.0000347.s004.pdf]

S1. *Pooled Effects of Psychotherapy versus Control Groups, Based on Different Calculation Methods of the SMD.*

Analysis Model	Calculation Method (SMD)	SMD	95% CI	I^2	95% CI	95% PI	NNT
**Assuming ρ=0.2**							
Combined	$SMD_{EP/EP}$	0.76	[0.70; 0.83]	84.04	[82.77; 85.22]	[-0.44; 1.97]	3.72
	$SMD_{CS/EP}$	0.80	[0.73; 0.87]	79.98	[78.27; 81.56]	[-0.45; 2.06]	3.51
	$SMD_{CS/CS}$	0.65	[0.59; 0.70]	72.91	[70.38; 75.22]	[-0.26; 1.55]	4.49
	$SMD_{CS/BL}$	0.90	[0.82; 0.98]	82.73	[81.32; 84.03]	[-0.42; 2.21]	3.10
Influence Analysis	$SMD_{EP/EP}$	0.68	[0.63; 0.73]	78.84	[76.98; 80.55]	[-0.16; 1.52]	4.26
	$SMD_{CS/EP}$	0.70	[0.65; 0.75]	71.33	[68.58; 73.85]	[-0.11; 1.52]	4.08
	$SMD_{CS/CS}$	0.58	[0.54; 0.62]	60.16	[55.97; 63.95]	[-0.01; 1.17]	5.10
	$SMD_{CS/BL}$	0.81	[0.75; 0.87]	77.44	[75.43; 79.30]	[-0.11; 1.73]	3.48
One ES/study (highest)	$SMD_{EP/EP}$	0.88	[0.79; 0.98]	86.78	[85.67; 87.81]	[-0.59; 2.36]	3.15
	$SMD_{CS/EP}$	0.94	[0.84; 1.03]	83.63	[82.16; 84.97]	[-0.58; 2.45]	2.96
	$SMD_{CS/CS}$	0.74	[0.67; 0.81]	76.97	[74.68; 79.05]	[-0.32; 1.81]	3.84
	$SMD_{CS/BL}$	1.06	[0.96; 1.16]	86.04	[84.85; 87.14]	[-0.53; 2.65]	2.58
One ES/study (lowest)	$SMD_{EP/EP}$	0.67	[0.6; 0.75]	83.69	[82.23; 85.03]	[-0.56; 1.91]	4.29
	$SMD_{CS/EP}$	0.70	[0.62; 0.78]	79.20	[77.20; 81.02]	[-0.56; 1.97]	4.09
	$SMD_{CS/CS}$	0.56	[0.49; 0.62]	71.15	[68.10; 73.92]	[-0.32; 1.43]	5.33
	$SMD_{CS/BL}$	0.77	[0.68; 0.86]	82.06	[80.41; 83.57]	[-0.52; 2.06]	3.68
Three-Level Model (CHE)	$SMD_{EP/EP}$	0.78	[0.71; 0.86]	90.10	-	[-0.59; 2.16]	3.61
	$SMD_{CS/EP}$	0.82	[0.75; 0.90]	86.40	-	[-0.59; 2.24]	3.42
	$SMD_{CS/CS}$	0.65	[0.60; 0.71]	76.40	-	[-0.35; 1.65]	4.43
	$SMD_{CS/BL}$	0.92	[0.84; 1.00]	86.70	-	[-0.55; 2.39]	3.02
**Assuming ρ=0.4**							
Combined	$SMD_{EP/EP}$	0.76	[0.70; 0.83]	84.04	[82.77; 85.22]	[-0.44; 1.97]	3.72
	$SMD_{CS/EP}$	0.80	[0.73; 0.87]	83.47	[82.14; 84.71]	[-0.48; 2.09]	3.51
	$SMD_{CS/CS}$	0.74	[0.68; 0.81]	81.52	[79.98; 82.94]	[-0.37; 1.86]	3.84
	$SMD_{CS/BL}$	0.90	[0.82; 0.97]	85.75	[84.65; 86.77]	[-0.45; 2.25]	3.11
Influence Analysis	$SMD_{EP/EP}$	0.68	[0.63; 0.73]	78.84	[76.98; 80.55]	[-0.16; 1.52]	4.26
	$SMD_{CS/EP}$	0.71	[0.66; 0.76]	77.32	[75.28; 79.19]	[-0.16; 1.58]	4.05
	$SMD_{CS/CS}$	0.67	[0.62; 0.71]	74.76	[72.43; 76.90]	[-0.10; 1.44]	4.34
	$SMD_{CS/BL}$	0.81	[0.75; 0.87]	81.99	[80.49; 83.38]	[-0.16; 1.78]	3.48
One ES/study (highest)	$SMD_{EP/EP}$	0.88	[0.79; 0.98]	86.78	[85.67; 87.81]	[-0.59; 2.36]	3.15
	$SMD_{CS/EP}$	0.94	[0.85; 1.04]	86.38	[85.22; 87.44]	[-0.61; 2.49]	2.94
	$SMD_{CS/CS}$	0.86	[0.78; 0.94]	84.21	[82.81; 85.50]	[-0.44; 2.16]	3.26
	$SMD_{CS/BL}$	1.07	[0.96; 1.17]	88.42	[87.49; 89.29]	[-0.56; 2.69]	2.57
One ES/study (lowest)	$SMD_{EP/EP}$	0.67	[0.60; 0.75]	83.69	[82.23; 85.03]	[-0.56; 1.91]	4.29
	$SMD_{CS/EP}$	0.70	[0.62; 0.79]	82.93	[81.38; 84.35]	[-0.60; 2.01]	4.07
	$SMD_{CS/CS}$	0.64	[0.57; 0.71]	80.58	[78.75; 82.26]	[-0.46; 1.74]	4.52
	$SMD_{CS/BL}$	0.77	[0.69; 0.86]	85.32	[84.05; 86.50]	[-0.56; 2.11]	3.67
Three-Level Model (CHE)	$SMD_{EP/EP}$	0.78	[0.71; 0.86]	90.10	-	[-0.59; 2.16]	3.61
	$SMD_{CS/EP}$	0.83	[0.75; 0.90]	89.80	-	[-0.63; 2.28]	3.40
	$SMD_{CS/CS}$	0.76	[0.69; 0.82]	86.40	-	[-0.47; 1.98]	3.76
	$SMD_{CS/BL}$	0.92	[0.84; 1.00]	90.00	-	[-0.58; 2.43]	3.01
**Assuming ρ=0.6**							
Combined	$SMD_{EP/EP}$	0.76	[0.7; 0.83]	84.04	[82.77; 85.22]	[-0.44; 1.97]	3.72
	$SMD_{CS/EP}$	0.80	[0.73; 0.87]	87.28	[86.33; 88.16]	[-0.52; 2.12]	3.52
	$SMD_{CS/CS}$	0.89	[0.81; 0.96]	88.86	[88.06; 89.61]	[-0.52; 2.30]	3.14
	$SMD_{CS/BL}$	0.89	[0.81; 0.97]	89.04	[88.26; 89.77]	[-0.49; 2.28]	3.12

Analysis Model	Calculation Method (SMD)	SMD	95% CI	I^2	95% CI	95% PI	NNT
Influence Analysis	$SMD_{EP/EP}$	0.68	[0.63; 0.73]	78.84	[76.98; 80.55]	[-0.16; 1.52]	4.26
	$SMD_{CS/EP}$	0.71	[0.66; 0.76]	83.38	[82.01; 84.64]	[-0.21; 1.62]	4.06
	$SMD_{CS/CS}$	0.81	[0.75; 0.86]	86.34	[85.29; 87.32]	[-0.24; 1.85]	3.49
	$SMD_{CS/BL}$	0.80	[0.75; 0.86]	86.65	[85.63; 87.60]	[-0.20; 1.81]	3.51
One ES/study (highest)	$SMD_{EP/EP}$	0.88	[0.79; 0.98]	86.78	[85.67; 87.81]	[-0.59; 2.36]	3.15
	$SMD_{CS/EP}$	0.95	[0.85; 1.04]	89.39	[88.56; 90.17]	[-0.64; 2.53]	2.92
	$SMD_{CS/CS}$	1.04	[0.94; 1.14]	90.38	[89.65; 91.06]	[-0.59; 2.67]	2.64
	$SMD_{CS/BL}$	1.07	[0.97; 1.17]	91.02	[90.34; 91.64]	[-0.58; 2.72]	2.56
One ES/study (lowest)	$SMD_{EP/EP}$	0.67	[0.60; 0.75]	83.69	[82.23; 85.03]	[-0.56; 1.91]	4.29
	$SMD_{CS/EP}$	0.71	[0.63; 0.79]	86.99	[85.90; 87.99]	[-0.64; 2.06]	4.06
	$SMD_{CS/CS}$	0.78	[0.69; 0.86]	88.56	[87.64; 89.41]	[-0.64; 2.19]	3.65
	$SMD_{CS/BL}$	0.77	[0.69; 0.86]	88.86	[87.97; 89.68]	[-0.60; 2.15]	3.67
Three-Level Model (CHE)	$SMD_{EP/EP}$	0.78	[0.71; 0.86]	90.10	-	[-0.59; 2.16]	3.61
	$SMD_{CS/EP}$	0.83	[0.75; 0.91]	93.10	-	[-0.66; 2.32]	3.39
	$SMD_{CS/CS}$	0.92	[0.84; 1.00]	93.50	-	[-0.64; 2.47]	3.04
	$SMD_{CS/BL}$	0.93	[0.85; 1.00]	93.10	-	[-0.61; 2.47]	3.00
**Assuming ρ=0.8**							
Combined	$SMD_{EP/EP}$	0.76	[0.70; 0.83]	84.04	[82.77; 85.22]	[-0.44; 1.97]	3.72
	$SMD_{CS/EP}$	0.79	[0.72; 0.87]	91.70	[91.15; 92.21]	[-0.56; 2.15]	3.56
	$SMD_{CS/CS}$	1.16	[1.06; 1.27]	94.82	[94.53; 95.10]	[-0.79; 3.12]	2.35
	$SMD_{CS/BL}$	0.88	[0.80; 0.96]	92.82	[92.37; 93.24]	[-0.54; 2.30]	3.18
Influence Analysis	$SMD_{EP/EP}$	0.68	[0.63; 0.73]	78.84	[76.98; 80.55]	[-0.16; 1.52]	4.26
	$SMD_{CS/EP}$	0.71	[0.66; 0.76]	90.18	[89.49; 90.82]	[-0.28; 1.69]	4.06
	$SMD_{CS/CS}$	1.06	[0.98; 1.13]	94.27	[93.93; 94.59]	[-0.44; 2.55]	2.59
	$SMD_{CS/BL}$	0.79	[0.73; 0.85]	91.73	[91.19; 92.25]	[-0.26; 1.84]	3.58
One ES/study (highest)	$SMD_{EP/EP}$	0.88	[0.79; 0.98]	86.78	[85.67; 87.81]	[-0.59; 2.36]	3.15
	$SMD_{CS/EP}$	0.95	[0.85; 1.05]	92.92	[92.43; 93.38]	[-0.67; 2.57]	2.91
	$SMD_{CS/CS}$	1.41	[1.28; 1.54]	95.4	[95.13; 95.67]	[-0.85; 3.67]	1.94
	$SMD_{CS/BL}$	1.07	[0.97; 1.18]	94.0	[93.61; 94.37]	[-0.61; 2.76]	2.55
One ES/study (lowest)	$SMD_{EP/EP}$	0.67	[0.6; 0.75]	83.69	[82.23; 85.03]	[-0.56; 1.91]	4.29
	$SMD_{CS/EP}$	0.71	[0.63; 0.79]	91.64	[91.03; 92.21]	[-0.69; 2.11]	4.04
	$SMD_{CS/CS}$	1.05	[0.93; 1.16]	94.85	[94.53; 95.16]	[-0.93; 3.02]	2.62
	$SMD_{CS/BL}$	0.77	[0.69; 0.86]	92.87	[92.38; 93.33]	[-0.64; 2.19]	3.66
Three-Level Model (CHE)	$SMD_{EP/EP}$	0.78	[0.71; 0.86]	90.10	-	[-0.59; 2.16]	3.61
	$SMD_{CS/EP}$	0.83	[0.76; 0.91]	96.40	-	[-0.70; 2.37]	3.37
	$SMD_{CS/CS}$	1.24	[1.13; 1.35]	97.90	-	[-0.93; 3.41]	2.20
	$SMD_{CS/BL}$	0.93	[0.85; 1.01]	96.30	-	[-0.65; 2.51]	2.99

Note. $SMD_{EP/EP}$ = SMD calculated by dividing the mean endpoint difference by the pooled endpoint SD; $SMD_{CS/BL}$ = SMD calculated by dividing the mean change score difference by the pooled baseline SD; $SMD_{CS/CS}$ = SMD calculated by dividing the mean change score difference by the pooled change score SD; $SMD_{CS/EP}$ = SMD calculated by dividing the mean change score difference by the pooled endpoint SD.
