# Supplementary material for: Standardized effect sizes are far from “Standardized”: A primer and empirical illustration in depression psychotherapy meta-analyses
Source: PLOS Ment Health. 2025 Jul 1;2(7):e0000347. doi: 10.1371/journal.pmen.0000347 (PMC12798590; doi:10.1371/journal.pmen.0000347)
Supplement: S2 Table — SMDEP/EP = SMD calculated by dividing the mean endpoint difference by the pooled endpoint SD; SMDCS/BL = SMD calculated by dividing the mean change score difference by the pooled baseline SD; SMDCS/CS = SMD calculated by dividing the mean change score difference by the pooled change score SD; SMDCS/EP = SMD calculated by dividing the mean change score difference by the pooled endpoint SD. (PDF) [file pmen.0000347.s005.pdf]

S2. *Pooled Effects of Psychotherapy versus Pharmacotherapy Trials, Based on Different Calculation Methods of the SMD.*

Analysis Model	Calculation Method (SMD)	SMD	95% CI	I^2	95% CI	95% PI	NNT
**Assuming ρ=0.2**							
Combined	$SMD_{EP/EP}$	0.10	[-0.06; 0.25]	82.10	[77.40; 85.82]	[-0.94; 1.14]	35.50
	$SMD_{CS/EP}$	0.06	[-0.11; 0.23]	78.67	[72.75; 83.30]	[-1.04; 1.16]	58.02
	$SMD_{CS/CS}$	0.03	[-0.10; 0.17]	69.68	[60.31; 76.84]	[-0.79; 0.85]	107.08
	$SMD_{CS/BL}$	0.02	[-0.17; 0.22]	80.85	[75.72; 84.90]	[-1.18; 1.23]	151.65
Influence Analysis	$SMD_{EP/EP}$	0.02	[-0.10; 0.14]	63.54	[51.50; 72.59]	[-0.61; 0.66]	161.36
	$SMD_{CS/EP}$	-0.02	[-0.15; 0.11]	56.25	[40.99; 67.56]	[-0.64; 0.60]	166.86
	$SMD_{CS/CS}$	-0.05	[-0.15; 0.05]	37.28	[13.09; 54.74]	[-0.35; 0.25]	70.56
	$SMD_{CS/BL}$	-0.06	[-0.21; 0.10]	65.29	[53.99; 73.81]	[-0.85; 0.74]	63.36
One ES/study (highest)	$SMD_{EP/EP}$	0.20	[0.02; 0.38]	82.81	[77.90; 86.63]	[-0.88; 1.28]	16.35
	$SMD_{CS/EP}$	0.17	[-0.02; 0.37]	80.19	[74.27; 84.74]	[-1.00; 1.35]	19.18
	$SMD_{CS/CS}$	0.13	[-0.03; 0.29]	71.24	[61.58; 78.47]	[-0.74; 0.99]	26.56
	$SMD_{CS/BL}$	0.18	[-0.04; 0.39]	81.32	[75.84; 85.56]	[-1.03; 1.38]	18.97
One ES/study (lowest)	$SMD_{EP/EP}$	0.05	[-0.13; 0.22]	83.33	[78.61; 87.00]	[-1.05; 1.14]	74.70
	$SMD_{CS/EP}$	-0.03	[-0.22; 0.17]	80.21	[74.30; 84.76]	[-1.20; 1.15]	141.31
	$SMD_{CS/CS}$	-0.04	[-0.19; 0.11]	71.22	[61.55; 78.45]	[-0.92; 0.84]	91.61
	$SMD_{CS/BL}$	-0.10	[-0.31; 0.11]	82.05	[76.86; 86.08]	[-1.39; 1.19]	34.39
Three-Level Model (CHE)	$SMD_{EP/EP}$	0.13	[-0.04; 0.30]	84.90	-	[-0.95; 1.21]	25.99
	$SMD_{CS/EP}$	0.09	[-0.10; 0.28]	80.40	-	[-1.07; 1.24]	40.23
	$SMD_{CS/CS}$	0.05	[-0.10; 0.21]	69.30	-	[-0.80; 0.91]	64.77
	$SMD_{CS/BL}$	0.05	[-0.15; 0.26]	82.30	-	[-1.20; 1.31]	64.47
**Assuming ρ=0.4**							
Combined	$SMD_{EP/EP}$	0.10	[-0.06; 0.25]	82.10	[77.4;0 85.82]	[-0.94; 1.14]	35.50
	$SMD_{CS/EP}$	0.06	[-0.11; 0.23]	82.71	[78.23; 86.27]	[-1.07; 1.20]	54.69
	$SMD_{CS/CS}$	0.05	[-0.11; 0.20]	80.43	[75.15; 84.59]	[-0.97; 1.06]	75.78
	$SMD_{CS/BL}$	0.03	[-0.16; 0.22]	84.48	[80.58; 87.59]	[-1.21; 1.27]	127.27
Influence Analysis	$SMD_{EP/EP}$	0.02	[-0.10; 0.14]	63.54	[51.50; 72.59]	[-0.61; 0.66]	161.36
	$SMD_{CS/EP}$	-0.01	[-0.14; 0.12]	65.85	[54.79; 74.20]	[-0.74; 0.72]	414.73
	$SMD_{CS/CS}$	-0.02	[-0.15; 0.10]	61.27	[48.25; 71.02]	[-0.64; 0.59]	145.11
	$SMD_{CS/BL}$	-0.04	[-0.20; 0.11]	72.55	[64.24; 78.93]	[-0.92; 0.83]	80.15
One ES/study (highest)	$SMD_{EP/EP}$	0.20	[0.02; 0.38]	82.81	[77.90; 86.63]	[-0.88; 1.28]	16.35
	$SMD_{CS/EP}$	0.18	[-0.02; 0.38]	83.93	[79.44; 87.44]	[-1.03; 1.38]	18.59
	$SMD_{CS/CS}$	0.16	[-0.02; 0.34]	81.45	[76.03; 85.65]	[-0.90; 1.22]	21.42
	$SMD_{CS/BL}$	0.18	[-0.03; 0.40]	84.90	[80.77; 88.15]	[-1.05; 1.42]	18.35
One ES/study (lowest)	$SMD_{EP/EP}$	0.05	[-0.13; 0.22]	83.33	[78.61; 87.00]	[-1.05; 1.14]	74.70
	$SMD_{CS/EP}$	-0.02	[-0.22; 0.17]	83.96	[79.48; 87.46]	[-1.22; 1.18]	152.94
	$SMD_{CS/CS}$	-0.04	[-0.21; 0.14]	81.53	[76.14; 85.71]	[-1.11; 1.03]	90.95
	$SMD_{CS/BL}$	-0.10	[-0.31; 0.12]	85.57	[81.68; 88.64]	[-1.43; 1.23]	35.25
Three-Level Model (CHE)	$SMD_{EP/EP}$	0.13	[-0.04; 0.30]	84.90	-	[-0.95; 1.21]	25.99
	$SMD_{CS/EP}$	0.09	[-0.10; 0.28]	85.20	-	[-1.10; 1.28]	38.50
	$SMD_{CS/CS}$	0.07	[-0.10; 0.24]	82.00	-	[-0.99; 1.13]	49.04
	$SMD_{CS/BL}$	0.06	[-0.14; 0.26]	86.90	-	[-1.24; 1.35]	60.09
**Assuming ρ=0.6**							
Combined	$SMD_{EP/EP}$	0.10	[-0.06; 0.25]	82.10	[77.40; 85.82]	[-0.94; 1.14]	35.50
	$SMD_{CS/EP}$	0.07	[-0.10; 0.24]	86.87	[83.76; 89.39]	[-1.10; 1.24]	51.60
	$SMD_{CS/CS}$	0.07	[-0.12; 0.25]	88.53	[85.92; 90.66]	[-1.24; 1.37]	53.29
	$SMD_{CS/BL}$	0.03	[-0.16; 0.22]	88.24	[85.54; 90.43]	[-1.24; 1.30]	106.54

Analysis Model	Calculation Method (SMD)	SMD	95% CI	I^2	95% CI	95% PI	NNT
Influence Analysis	$SMD_{EP/EP}$	0.02	[-0.10; 0.14]	63.54	[51.50; 72.59]	[-0.61; 0.66]	161.36
	$SMD_{CS/EP}$	0.00	[-0.13; 0.14]	75.60	[68.47; 81.11]	[-0.83; 0.83]	1031.83
	$SMD_{CS/CS}$	0.00	[-0.15; 0.15]	79.46	[73.78; 83.91]	[-0.95; 0.95]	1232.80
	$SMD_{CS/BL}$	-0.03	[-0.19; 0.13]	80.11	[74.66; 84.39]	[-0.99; 0.93]	109.98
One ES/study (highest)	$SMD_{EP/EP}$	0.20	[0.02; 0.38]	82.81	[77.90; 86.63]	[-0.88; 1.28]	16.35
	$SMD_{CS/EP}$	0.18	[-0.01; 0.38]	87.78	[84.65; 90.27]	[-1.06; 1.43]	17.95
	$SMD_{CS/CS}$	0.20	[-0.02; 0.42]	89.14	[86.46; 91.28]	[-1.16; 1.56]	16.43
	$SMD_{CS/BL}$	0.19	[-0.03; 0.40]	88.62	[85.77; 90.89]	[-1.09; 1.46]	17.69
One ES/study (lowest)	$SMD_{EP/EP}$	0.05	[-0.13; 0.22]	83.33	[78.61; 87.00]	[-1.05; 1.14]	74.70
	$SMD_{CS/EP}$	-0.02	[-0.21; 0.17]	87.82	[84.71; 90.30]	[-1.25; 1.21]	170.95
	$SMD_{CS/CS}$	-0.04	[-0.25; 0.17]	89.26	[86.62; 91.37]	[-1.40; 1.33]	94.18
	$SMD_{CS/BL}$	-0.09	[-0.31; 0.12]	89.19	[86.53; 91.32]	[-1.47; 1.28]	36.38
Three-Level Model (CHE)	$SMD_{EP/EP}$	0.13	[-0.04; 0.30]	84.90	-	[-0.95; 1.21]	25.99
	$SMD_{CS/EP}$	0.09	[-0.10; 0.28]	90.10	-	[-1.13; 1.32]	36.80
	$SMD_{CS/CS}$	0.10	[-0.11; 0.30]	91.80	-	[-1.26; 1.45]	36.12
	$SMD_{CS/BL}$	0.06	[-0.14; 0.27]	91.40	-	[-1.29; 1.41]	55.76
**Assuming ρ=0.8**							
Combined	$SMD_{EP/EP}$	0.10	[-0.06; 0.25]	82.10	[77.4;0 85.82]	[-0.94; 1.14]	35.50
	$SMD_{CS/EP}$	0.07	[-0.10; 0.24]	91.37	[89.58; 92.85]	[-1.13; 1.27]	49.01
	$SMD_{CS/CS}$	0.10	[-0.15; 0.35]	94.42	[93.42; 95.27]	[-1.68; 1.89]	34.20
	$SMD_{CS/BL}$	0.04	[-0.15; 0.23]	92.34	[90.81; 93.61]	[-1.25; 1.33]	86.12
Influence Analysis	$SMD_{EP/EP}$	0.02	[-0.10; 0.14]	63.54	[51.50; 72.59]	[-0.61; 0.66]	161.36
	$SMD_{CS/EP}$	0.01	[-0.12; 0.15]	85.73	[82.22; 88.55]	[-0.90; 0.93]	245.73
	$SMD_{CS/CS}$	0.02	[-0.18; 0.22]	91.93	[90.28; 93.30]	[-1.35; 1.39]	193.09
	$SMD_{CS/BL}$	-0.02	[-0.18; 0.14]	88.22	[85.50; 90.44]	[-1.04; 1.00]	190.5
One ES/study (highest)	$SMD_{EP/EP}$	0.20	[0.02; 0.38]	82.81	[77.90; 86.63]	[-0.88; 1.28]	16.35
	$SMD_{CS/EP}$	0.19	[-0.01; 0.39]	91.94	[90.14; 93.41]	[-1.10; 1.48]	17.26
	$SMD_{CS/CS}$	0.28	[-0.01; 0.58]	94.77	[93.76; 95.62]	[-1.65; 2.22]	11.32
	$SMD_{CS/BL}$	0.19	[-0.02; 0.41]	92.68	[91.10; 93.98]	[-1.13; 1.52]	17.00
One ES/study (lowest)	$SMD_{EP/EP}$	0.05	[-0.13; 0.22]	83.33	[78.61; 87.00]	[-1.05; 1.14]	74.7
	$SMD_{CS/EP}$	-0.02	[-0.21; 0.17]	92.00	[90.22; 93.46]	[-1.29; 1.26]	201.15
	$SMD_{CS/CS}$	-0.03	[-0.32; 0.25]	94.84	[93.85; 95.67]	[-1.97; 1.90]	108.80
	$SMD_{CS/BL}$	-0.09	[-0.31; 0.13]	93.08	[91.61; 94.29]	[-1.52; 1.34]	37.87
Three-Level Model (CHE)	$SMD_{EP/EP}$	0.13	[-0.04; 0.30]	84.90	-	[-0.95; 1.21]	25.99
	$SMD_{CS/EP}$	0.10	[-0.09; 0.29]	95.10	-	[-1.17; 1.37]	35.07
	$SMD_{CS/CS}$	0.14	[-0.14; 0.42]	97.70	-	[-1.78; 2.06]	23.84
	$SMD_{CS/BL}$	0.07	[-0.14; 0.27]	95.80	-	[-1.34; 1.48]	51.50

Note. $SMD_{EP/EP}$ = SMD calculated by dividing the mean endpoint difference by the pooled endpoint SD; $SMD_{CS/BL}$ = SMD calculated by dividing the mean change score difference by the pooled baseline SD; $SMD_{CS/CS}$ = SMD calculated by dividing the mean change score difference by the pooled change score SD; $SMD_{CS/EP}$ = SMD calculated by dividing the mean change score difference by the pooled endpoint SD.
