# Supplementary material for: Standardized effect sizes are far from “Standardized”: A primer and empirical illustration in depression psychotherapy meta-analyses
Source: PLOS Ment Health. 2025 Jul 1;2(7):e0000347. doi: 10.1371/journal.pmen.0000347 (PMC12798590; doi:10.1371/journal.pmen.0000347)
Supplement: S3 Table — ΔSMD = Difference between the pooled effect based on endpoint SMDs (SMDEP/EP), and SMDs calculated using change scores (SMDCS). “Attrition” refers to the proportion of participants who were lost to follow-up, pooled across both trial arms (continuous covariate); “Baseline Imbalance” to the absolute value of the between-group SMD at baseline (continuous covariate); and “Risk of Bias” to the number of domains assessed to have a low risk of bias (continuous covariate; 0–4). (PDF) [file pmen.0000347.s006.pdf]

S3. *Divergent Effect Estimates, Conditional on Study and Treatment Characteristics (Psychotherapy versus Control).*

Calculation Method (SMD)	Variable Type	Moderator/Subgroup	Δ_{SMD}	S.E.	Z	p
**Assuming ρ=0.2**						
Change Scores (Baseline SD)	*Trial Characteristics*	Risk of Bias (SD)	0.053	0.019	2.767	0.006
		Attrition (SD)	0.009	0.017	0.504	0.614
		Baseline Imbalance	-0.015	0.021	-0.753	0.452
	Treatments	Third-Wave CBT	0.099	0.065	1.525	0.127
		Behavioral Activation	0.259	0.060	4.324	<0.001
		CBT	0.169	0.023	7.335	<0.001
		Psychodynamic	0.180	0.105	1.717	0.086
		Interpersonal	0.230	0.076	3.027	0.002
		Life Review	0.206	0.108	1.902	0.057
		Problem-Solving	0.196	0.074	2.642	0.008
		Supportive Counseling	0.169	0.101	1.671	0.095
	Comparator	Care As Usual	0.181	0.025	7.231	<0.001
		Other Control	0.176	0.039	4.568	<0.001
		Waitlist	0.180	0.029	6.261	<0.001
Change Scores (Endpoint SD)	*Trial Characteristics*	Risk of Bias (SD)	0.022	0.018	1.260	0.208
		Attrition (SD)	0.012	0.015	0.768	0.443
		Baseline Imbalance	-0.056	0.020	-2.858	0.004
	Treatments	Third-Wave CBT	0.014	0.061	0.228	0.820
		Behavioral Activation	0.059	0.055	1.061	0.289
		CBT	0.036	0.021	1.754	0.079
		Psychodynamic	0.036	0.091	0.390	0.697
		Interpersonal	0.019	0.071	0.275	0.784
		Life Review	0.132	0.101	1.306	0.192
		Problem-Solving	0.094	0.070	1.342	0.180
		Supportive Counseling	0.046	0.097	0.475	0.635
	Comparator	Care As Usual	0.054	0.022	2.428	0.015
		Other Control	0.003	0.034	0.085	0.932
		Waitlist	0.051	0.026	1.974	0.048
Change Scores (Change SD)	*Trial Characteristics*	Risk of Bias (SD)	-0.013	0.018	-0.725	0.468
		Attrition (SD)	-0.020	0.016	-1.257	0.209
		Baseline Imbalance	-0.030	0.020	-1.541	0.123
	Treatments	Third-Wave CBT	-0.147	0.061	-2.413	0.016
		Behavioral Activation	-0.081	0.056	-1.451	0.147
		CBT	-0.102	0.021	-4.821	<0.001
		Psychodynamic	-0.025	0.095	-0.269	0.788
		Interpersonal	-0.071	0.072	-0.996	0.319
		Life Review	-0.069	0.101	-0.676	0.499
		Problem-Solving	-0.088	0.071	-1.249	0.212
		Supportive Counseling	-0.093	0.097	-0.963	0.336
	Comparator	Care As Usual	-0.066	0.023	-2.873	0.004
		Other Control	-0.100	0.035	-2.812	0.005
		Waitlist	-0.115	0.027	-4.333	<0.001
**Assuming ρ=0.4**						
Change Scores (Baseline SD)	*Trial Characteristics*	Risk of Bias (SD)	0.049	0.019	2.651	0.008
		Attrition (SD)	0.003	0.017	0.160	0.873
		Baseline Imbalance	-0.014	0.019	-0.697	0.486
	Treatments	Third-Wave CBT	0.090	0.061	1.469	0.142
		Behavioral Activation	0.257	0.057	4.467	<0.001
		CBT	0.157	0.022	7.106	<0.001
		Psychodynamic	0.186	0.101	1.837	0.066

Calculation Method (SMD)	Variable Type	Moderator/Subgroup	Δ_{SMD}	S.E.	*Z*	*p*
		Interpersonal	0.226	0.072	3.158	0.002
		Life Review	0.197	0.102	1.923	0.054
		Problem-Solving	0.187	0.070	2.647	0.008
		Supportive Counseling	0.163	0.097	1.687	0.092
	Comparator	Care As Usual	0.173	0.024	7.215	<0.001
		Other Control	0.165	0.037	4.431	<0.001
		Waitlist	0.170	0.028	6.192	<0.001
Change Scores (Endpoint SD)	*Trial Characteristics*	Risk of Bias (SD)	0.020	0.016	1.248	0.212
		Attrition (SD)	0.011	0.014	0.790	0.430
		Baseline Imbalance	-0.052	0.018	-2.833	0.005
	Treatments	Third-Wave CBT	0.010	0.056	0.175	0.861
		Behavioral Activation	0.055	0.051	1.081	0.280
		CBT	0.029	0.019	1.510	0.131
		Psychodynamic	0.034	0.084	0.404	0.686
		Interpersonal	0.018	0.066	0.282	0.778
		Life Review	0.125	0.094	1.333	0.182
		Problem-Solving	0.082	0.065	1.265	0.206
		Supportive Counseling	0.042	0.089	0.477	0.633
	Comparator	Care As Usual	0.051	0.021	2.467	0.014
		Other Control	-0.002	0.031	-0.064	0.949
		Waitlist	0.042	0.024	1.744	0.081
Change Scores (Change SD)	*Trial Characteristics*	Risk of Bias (SD)	0.008	0.016	0.485	0.627
		Attrition (SD)	-0.003	0.014	-0.243	0.808
		Baseline Imbalance	-0.033	0.018	-1.826	0.068
	Treatments	Third-Wave CBT	-0.054	0.056	-0.971	0.332
		Behavioral Activation	0.020	0.051	0.387	0.698
		CBT	-0.012	0.019	-0.612	0.540
		Psychodynamic	0.024	0.083	0.294	0.768
		Interpersonal	0.010	0.065	0.159	0.874
		Life Review	0.035	0.093	0.378	0.705
		Problem-Solving	0.013	0.065	0.207	0.836
		Supportive Counseling	0.001	0.089	0.008	0.993
	Comparator	Care As Usual	0.012	0.020	0.600	0.549
		Other Control	-0.025	0.031	-0.802	0.422
		Waitlist	-0.011	0.024	-0.478	0.633
**Assuming ρ=0.6**						
Change Scores (Baseline SD)	*Trial Characteristics*	Risk of Bias (SD)	0.043	0.018	2.433	0.015
		Attrition (SD)	-0.005	0.016	-0.308	0.758
		Baseline Imbalance	-0.012	0.018	-0.654	0.513
	Treatments	Third-Wave CBT	0.078	0.058	1.343	0.179
		Behavioral Activation	0.254	0.055	4.594	<0.001
		CBT	0.141	0.021	6.673	<0.001
		Psychodynamic	0.194	0.098	1.982	0.047
		Interpersonal	0.221	0.067	3.286	0.001
		Life Review	0.185	0.096	1.914	0.056
		Problem-Solving	0.172	0.067	2.579	0.010
		Supportive Counseling	0.155	0.092	1.696	0.090
	Comparator	Care As Usual	0.163	0.023	7.056	<0.001
		Other Control	0.150	0.036	4.176	<0.001
		Waitlist	0.158	0.026	5.976	<0.001
Change Scores (Endpoint SD)	*Trial Characteristics*	Risk of Bias (SD)	0.018	0.015	1.183	0.237
		Attrition (SD)	0.010	0.013	0.799	0.425
		Baseline Imbalance	-0.046	0.017	-2.718	0.007
	Treatments	Third-Wave CBT	0.004	0.051	0.085	0.932
		Behavioral Activation	0.050	0.046	1.083	0.279
		CBT	0.019	0.017	1.082	0.279

Calculation Method (SMD)	Variable Type	Moderator/Subgroup	Δ_{SMD}	S.E.	Z	p
		Psychodynamic	0.032	0.076	0.417	0.677
		Interpersonal	0.017	0.060	0.285	0.775
		Life Review	0.116	0.086	1.351	0.177
		Problem-Solving	0.066	0.059	1.113	0.266
		Supportive Counseling	0.039	0.081	0.480	0.631
	Comparator	Care As Usual	0.046	0.019	2.461	0.014
		Other Control	-0.008	0.028	-0.286	0.775
		Waitlist	0.029	0.022	1.330	0.183
Change Scores (Change SD)	*Trial Characteristics*	Risk of Bias (SD)	0.040	0.016	2.522	0.012
		Attrition (SD)	0.018	0.013	1.376	0.169
		Baseline Imbalance	-0.035	0.017	-2.068	0.039
	Treatments	Third-Wave CBT	0.089	0.053	1.698	0.090
		Behavioral Activation	0.186	0.048	3.873	<0.001
		CBT	0.130	0.019	7.002	<0.001
		Psychodynamic	0.119	0.083	1.444	0.149
		Interpersonal	0.148	0.062	2.404	0.016
		Life Review	0.195	0.089	2.189	0.029
		Problem-Solving	0.177	0.061	2.928	0.003
		Supportive Counseling	0.156	0.082	1.910	0.056
	Comparator	Care As Usual	0.136	0.020	6.720	<0.001
		Other Control	0.094	0.031	3.039	0.002
		Waitlist	0.156	0.023	6.657	<0.001
**Assuming ρ=0.8**						
Change Scores (Baseline SD)	*Trial Characteristics*	Risk of Bias (SD)	0.034	0.017	1.971	0.049
		Attrition (SD)	-0.015	0.016	-0.975	0.330
		Baseline Imbalance	-0.011	0.017	-0.669	0.504
	Treatments	Third-Wave CBT	0.056	0.054	1.032	0.302
		Behavioral Activation	0.246	0.053	4.616	<0.001
		CBT	0.115	0.020	5.662	<0.001
		Psychodynamic	0.204	0.095	2.149	0.032
		Interpersonal	0.211	0.063	3.366	0.001
		Life Review	0.164	0.091	1.812	0.070
		Problem-Solving	0.145	0.063	2.307	0.021
		Supportive Counseling	0.146	0.087	1.684	0.092
	Comparator	Care As Usual	0.145	0.022	6.491	<0.001
		Other Control	0.127	0.035	3.627	<0.001
		Waitlist	0.135	0.025	5.299	<0.001
Change Scores (Endpoint SD)	*Trial Characteristics*	Risk of Bias (SD)	0.013	0.013	0.951	0.341
		Attrition (SD)	0.008	0.011	0.730	0.465
		Baseline Imbalance	-0.035	0.015	-2.356	0.018
	Treatments	Third-Wave CBT	-0.005	0.046	-0.104	0.917
		Behavioral Activation	0.043	0.041	1.036	0.300
		CBT	0.003	0.016	0.206	0.837
		Psychodynamic	0.028	0.067	0.421	0.674
		Interpersonal	0.015	0.053	0.276	0.783
		Life Review	0.103	0.077	1.339	0.181
		Problem-Solving	0.042	0.053	0.796	0.426
		Supportive Counseling	0.035	0.072	0.493	0.622
	Comparator	Care As Usual	0.038	0.017	2.301	0.021
		Other Control	-0.017	0.025	-0.661	0.508
		Waitlist	0.009	0.019	0.471	0.638
Change Scores (Change SD)	*Trial Characteristics*	Risk of Bias (SD)	0.098	0.024	4.042	<0.001
		Attrition (SD)	0.053	0.017	3.167	0.002
		Baseline Imbalance	-0.036	0.017	-2.085	0.037
	Treatments	Third-Wave CBT	0.353	0.059	6.002	<0.001
		Behavioral Activation	0.517	0.055	9.415	<0.001

Calculation Method (SMD)	Variable Type	Moderator/Subgroup	Δ_{SMD}	S.E.	*Z*	*p*
		CBT	0.408	0.027	15.077	<0.001
		Psychodynamic	0.366	0.130	2.823	0.005
		Interpersonal	0.456	0.067	6.763	<0.001
		Life Review	0.516	0.107	4.820	<0.001
		Problem-Solving	0.514	0.065	7.968	<0.001
		Supportive Counseling	0.486	0.079	6.176	<0.001
	Comparator	Care As Usual	0.380	0.033	11.505	<0.001
		Other Control	0.329	0.053	6.180	<0.001
		Waitlist	0.493	0.036	13.520	<0.001

Note. Δ_{SMD} = Difference between the pooled effect based on endpoint SMDs ($SMD_{EP/EP}$), and SMDs calculated using change scores (SMD_{CS}). "Attrition" refers to the proportion of participants who were lost to follow-up, pooled across both trial arms (continuous covariate); "Baseline Imbalance" to the absolute value of the between-group SMD at baseline (continuous covariate); and "Risk of Bias" to the number of domains assessed to have a low risk of bias (continuous covariate; 0-4).
