# Supplementary material for: Standardized effect sizes are far from “Standardized”: A primer and empirical illustration in depression psychotherapy meta-analyses
Source: PLOS Ment Health. 2025 Jul 1;2(7):e0000347. doi: 10.1371/journal.pmen.0000347 (PMC12798590; doi:10.1371/journal.pmen.0000347)
Supplement: Attachment — Submitted filename: response.pdf [file pmen.0000347.s008.pdf]

RESPONSE TO THE EDITOR AND REVIEWER COMMENTS

Editor.. 2
Reviewer 1 7
Reviewer 2 15

EDITOR

Dear Dr. Hagger-Johnson,

Thank you for giving us the opportunity to submit a revised version of our manuscript "Standardized Effect Sizes Are Far From 'Standardized': A Primer and Empirical Illustration in Depression Psychotherapy Meta-Analyses" to *PLOS Mental Health*. We thank both you and the two reviewers for your feedback, which has been very helpful to further improve the quality of our manuscript. A point-by-point response to the editor and reviewer comments can be found below.

COMMENT 1:

"Why not simulate data and then show what happens to the true effect under various scenarios, including missing data, intention to treat etc.? I agree with the reviewer on this point. I would have done the whole thing as a simulation and then used published studies as examples. I'm not suggesting to rework the paper entirely, but it would be useful to see at least a visual simulation study for comparison."

RESPONSE:

We agree with the editor that it is probably out of scope to supplement this paper with an additional full-scale simulation study; particularly since several already exist on this topic (e.g. [1,2,3]), including studies mentioned in the manuscript. In this paper, our primary goal was not to study the bias, coverage etc. of different SMD calculation methods via simulation. Instead, we wanted to assess how much meta-analytic effects of psychological treatment can realistically diverge depending on the chosen SMD calculation method, using the same dataset as basis. We believe this is important, since SMD values reported in different meta-analyses are often directly compared to each other.

Nonetheless, we also agree with the editor that adding a small-scale, "visual" simulation of the relationships we outline in our primer section is helpful. In S1 in the supplement, we now describe in detail the setup and results of a small simulation study we conducted, assuming "realistic" parameter values for psychological treatment trials. This simulation is now also referenced in the manuscript itself. The section includes a visual summary of our simulation, similar to the summary plot of our empirical investigation in Figure 1 in the main manuscript.

This visualization may help readers build a better intuition about the expected divergences between SMD calculation methods, presuming several simplifying assumptions are made. The simulation results added to S1 also closely mirror the findings we obtained in the empirical investigation. Furthermore, they align with our recommendation against SMD calculation methods that rely heavily on imputed pre-post correlations. We have also uploaded the source code of the simulation to the paper's open material repository now (doi.org/10.5281/zenodo.10694719).

References

[1] Gnambs, T., & Schroeders, U. (2024). Accuracy and precision of fixed and random effects in meta-analyses of randomized control trials for continuous outcomes. *Research synthesis methods*, *15*(1), 86-106.

[2] Hedges, L. V., Tipton, E., Zejnullahi, R., & Diaz, K. G. (2023). Effect sizes in ANCOVA and difference-in-differences designs. *British Journal of Mathematical and Statistical Psychology*, *76*(2), 259-282.
[3] McKenzie, J. E., Herbison, G. P., & Deeks, J. J. (2016). Impact of analysing continuous outcomes using final values, change scores and analysis of covariance on the performance of meta-analytic methods: a simulation study. *Research synthesis methods*, *7*(4), 371-386.

CHANGES TO THE MANUSCRIPT:
Page 10, Line 216: "Depending on what approaches are used, this may heavily limit the comparability of effect sizes within and across meta-analyses. Researchers could seriously over- or underestimate the efficacy of a treatment if SMD estimates are compared to other trials or meta-analyses using a different standardization method, or if wrong assumptions about the pre-post correlation are made. Results of our "toy" simulation shown in the supplement (S1) further illustrate this issue."

Page 19, Line 438: "Our results co-align with statistical theory, which predicts divergences between SMD estimates depending on the denominator used to "standardize" the effect (see, e.g., results of our simulation in supplement S1, which closely mirror our empirical findings). Holding the raw mean difference constant, SMDs will increase as the SD in the denominator decreases, and this effect will be most pronounced when the raw mean difference is large."

S1 added to the supplement (2 pages), showing the methodology and results of a simulation study assuming plausible true parameter values for psychological treatments.

COMMENT 2:
"Add specific definition of 'generalisability' (P4, L87)"

RESPONSE:
We now specify the definition of generalizability used in the cited paper.

CHANGES TO THE MANUSCRIPT:
Page 4, Line 85: "Even when the same scale was used, SMDs may sometimes be favored over unstandardized MDs. For instance, health professionals are more likely to interpret SMDs correctly [5,6], and SMDs may also offer somewhat greater generalizability compared to MDs (defined as lower cross-study variability not attributable to sampling error, and greater agreement in effect estimates across studies [7])."

COMMENT 3:
"I do not understand why selection of baseline, change or endpoint scores is an issue specific to standardization - the same choice might be made for unstandardized scores. I may have misunderstood this, but then so might readers, so it should be rewritten here to ensure it cannot be misunderstood."

RESPONSE:
In a successfully randomized trial, if (unstandardized) mean differences are computed, both change scores (differences-in-differences) and endpoint scores provide an

asymptotically unbiased estimator of the true treatment effect. Only their precision may differ. This behavior is explained in the primer section (see eq. 2 and 3), and illustrated by the third column in Figure 1: if the same standardizer is used for both change score and endpoint mean differences, the computed effect sizes hardly diverge at all. The same pattern can also be found in the newly added simulation study in the supplement (S1; see third row in the figure), where it can be seen that both change and endpoint scores provide an unbiased estimator of the true effect.

In our assessment, divergences in the calculated effect size are therefore strongly related to the *standardizer* that researchers choose to apply. Our empirical investigation shows that the effect of depression psychotherapy, expressed as the SMD, can differ strongly depending on the standardizer that has been applied. Our practical recommendation is to rely on endpoint SMDs, since their standardizer is clearly defined, and does not depend on pre-post correlations, which are typically not reported in trials.

We have now revised our manuscript to make this point clearer.

CHANGES TO THE MANUSCRIPT:
Page 6, Line 139: "In RCTs, this approach remains asymptotically unbiased but is generally less efficient than ANCOVA with baseline score adjustment [13]. Notably, mean differences derived from both change scores (equation 2) and endpoint scores (equation 3) provide unbiased estimates of the treatment effect in successfully randomized trials, though their efficiency may vary [11]."

Page 9, Line 199: "In sum, this shows that different SMD calculation methods can produce widely varying results, even when the true treatment effect is the same. These discrepancies primarily stem from the choice of standardizing denominator. In S1 in the supplement, we provide a visual summary of these predicted differences as obtained by a simulation study."

COMMENT 4:
"When discussing different instruments (e.g. P7L138 and elsewhere) please be clear if you are referring to depression specifically - lots of trials use instruments which are arguably 'common mental disorder' or 'psychological distress' (GHQ, HADS) or proxie for neuroticism, rather than depression specifically. You return to the issue of specific measures in the discussion, but it needs to be clear throughout and in relation to your selection criteria."

RESPONSE:
We thank the editor for pointing this out. The databases included in this study indeed focus explicitly on depressive symptoms severity. We now make this point clearer throughout the manuscript.

CHANGES TO THE MANUSCRIPT:
Page 7, Line 144: "A further complication in meta-analyses is that different instruments (e.g., depression scales) may have been used across studies, and that mean differences $\hat{\theta}$ estimated from each trial are therefore not comparable. This can be resolved by calculating a "unit-free" [18] measure of the effect, viz., the SMD."

Page 13, Line 292: “Arm-specific mean change scores (m_{CS}) in the eligible trials were calculated by subtracting the mean depressive symptoms score at baseline from the endpoint mean ($m_{EP} – m_{BL}$). We assumed that sample sizes for the change score (n_{CS}) were identical to the sample size available at the endpoint (n_{EP}).”

Page 15, Line 326: “For each of the SMD variants, we calculated the pooled effect of psychotherapy versus control groups, and of psychotherapy versus ADM, on depressive symptom severity.”

COMMENT 5:
“I am not an expert in RCT methods and was surprised/alarmed to see (P16L351) that sudies could be published without pre/post test means, SDs and sample sizes! Why were 532 - 443 studies even published? Is study quality really that permissably low, or has something happened with the inclusion criteria? Amend for clarity.”

RESPONSE:
Overall, less than one fifth (17%) of the 532 clinical trials in our psychotherapy vs. control database had to be removed because they did not report both pre-test and post-test means and standard deviations (27% for antidepressant vs. psychotherapy trials). It should be noted that our database includes trials going as far back as the early 1980s, which is decades before reporting guidelines became common. Effect sizes in clinical trials can be calculated using endpoint scores alone, so personally we were not too surprised to see that some studies did not report pre-test scores. Furthermore, an SMD *could* be calculated even in the studies we excluded; just not using *all* the calculation methods we wanted to examine here (cf. the “Datasets” section, where this rationale is explained). In some psychotherapy trials, the primary outcome is also dichotomous by definition (e.g. diagnosis of depression), which means that an SMD can only be obtained via conversion formulas. However, suboptimal reporting indeed remains a problem in randomized (psychotherapy) trials. We now revised our discussion section to comment on this point.

CHANGES TO THE MANUSCRIPT:
Page 21, Line 493: “In our analysis, 16.7% to 27.3% of trials had to be removed because they did not report group means and standard deviations at both pre- and post-test. This indicates that, in general, outcome reporting in psychotherapy trials needs to be improved. To enhance transparency, researchers may also provide a pre-specification of the SMD calculation method they plan to employ, to prevent selective reporting of the one variant yielding the highest effect size. In this context, it is important to re-emphasize that none of the SMD calculation methods we examined here is inherently “wrong” or biased. Researchers may still select a different variant than $SMD_{EP/EP}$ for their analysis; but this choice should be clearly described and justified, since it could limit the comparability of the effect size.”

COMMENT 6:
“'SMDs can be much less "standardized" than their name suggests: [other details]' would make a nice title for the paper, it conveys the crux of the message. If not, it

should in my opinion be used in any summaries, press releases, dissemination activities etc.”

RESPONSE:
We agree with the editor, and have now changed the title of the paper to “Standardized Effect Sizes Are Far From ‘Standardized’: A Primer and Empirical Illustration in Depression Psychotherapy Meta-Analyses”. We believe this conveys the main issue addressed in our study (“standardization” of effect sizes), and this new title might also catch readers’ attention.

CHANGES TO THE MANUSCRIPT:
Title changed to “Standardized Effect Sizes Are Far From ‘Standardized’: A Primer and Empirical Illustration in Depression Psychotherapy Meta-Analyses”.

COMMENT 7:
“Presumably some researchers will use different approaches to standardization and then publish the one giving the largest effect. Would pre-registration of the choice address this? Please add thoughts in relation to this (P21).”

RESPONSE:
We agree that detailed preregistration of the SMD calculation method could prevent “cherry-picking” of the one variant yielding the highest effect size. We have added this point to the discussion.

CHANGES TO THE MANUSCRIPT:
Page 21, Line 494: “This indicates that, in general, outcome reporting in psychotherapy trials needs to be improved. To enhance transparency, researchers may also provide a pre-specification of the SMD calculation method they plan to employ, to prevent selective reporting of the one variant yielding the highest effect size. In this context, it is important to re-emphasize that none of the SMD calculation methods we examined here is inherently “wrong” or biased. Researchers may still select a different variant than $SMD_{EP/EP}$ for their analysis; but this should be clearly described, since it could limit the comparability of the effect size.”

REVIEWER 1

COMMENT 1:
"This work presents the results of a methodological review on the consequences of using different effect size indices from the 'standardized mean difference' family in meta-analyses on the efficacy of psychological interventions. Although the study focuses on research about the efficacy of psychological interventions for treating depression, its findings are generalizable to other meta-analyses on the efficacy of psychological interventions and may even be applicable to other types of interventions in the Social and Health Sciences. The essential requirement for this applicability is that the empirical studies feeding these meta-analyses must have employed a pretest-posttest design with a control group (either with or without random assignment, i.e., experimental and quasi-experimental studies). In such studies, the basic descriptive statistics needed to apply any version of the 'standardized mean difference' are the pretest and posttest means and SDs of the two groups being compared, the SDs of the change scores, and, if possible, the Pearson correlation coefficients between the pretest and posttest scores for the two groups. The authors of this study have conducted a very comprehensive investigation by analyzing a large database of primary studies (particularly regarding comparisons between treated and control groups, with k = 395 studies and 791 effect sizes). In my opinion, publishing methodological reviews of this kind is highly important as they provide a realistic overview of the extent to which using different effect size indices can impact the results of a meta-analysis. However, I find certain aspects of the work that would require further theoretical and conceptual development. My interpretation of the results of this research does not align with the authors' conclusions. Therefore, my recommendation is that the authors undertake a thorough revision of the aspects that I present below.

RESPONSE:
We thank the reviewer for the thorough and detailed feedback, which has been very helpful to further improve the quality of our manuscript. This was much appreciated.

To summarize, we believe the reviewer raised several important arguments. Many of these points we generally agree with, and now try to emphasize more clearly in the manuscript. For example, we fully agree that endpoint SMDs should not be viewed as a general "gold standard" to compute effect sizes.

However, we do disagree on some points. In our understanding, the reviewer's central counter-argument seems to be that, in contrast to endpoint scores, change scores are a generally adequate method to adjust for baseline differences. We have to respectfully disagree with the reviewer on this point, which is frequently discussed in the statistical literature, and also in the current paper. Once this argument is set aside, we believe it also becomes clearer why we come to the conclusion that, in the context of randomized psychotherapy trials, and on purely pragmatic grounds, endpoint scores may typically be preferable. A more detailed, point-by-point response to the provided feedback can be found right below.

COMMENT 2:
"In the Introduction of this paper, it should be made clear that different indices from the 'standardized mean difference' family estimate different parameters. These

parameters differ both in the numerator and denominator of the effect size formula in question. In the numerator, there are essentially two options: the posttest mean difference and the difference between the pretest-posttest mean changes of the two groups (setting aside the use of adjusted posttest means for the pretest). In the denominator, there are different standardizers, each of which leads to estimating different parameters: SDEP, SDBL, and SDCS. Therefore, in the Introduction, each estimator should be linked to its corresponding parameter. Specifically, I am referring to the estimators SMDEP/EP, SMDCS/BL, SMDCS/CS, and SMDCS/EP."

RESPONSE:
We now make it clearer that SMDEP/EP, SMDCS/BL, SMDCS/CS, and SMDCS/EP are distinct estimators that differ in both their numerator and denominator.

CHANGES TO THE MANUSCRIPT:
Page 13, Line 303: "$SMD_{EP/EP}$, $SMD_{CS/BL}$, $SMD_{CS/CS}$ and $SMD_{CS/EP}$ represent distinct estimators, differing in both their numerator (endpoint vs. change scores) and denominator (baseline, endpoint, or change score SD), which may lead to substantial variations in the numeric value of the resulting effect size."

COMMENT 3:
"Using the posttest mean difference in the numerator implies disregarding the baseline levels of the two groups in the pretest, which could result in biased estimates of the true population effect. This is particularly important in quasi-experimental studies (i.e., without random assignment) but can also occur in experimental studies. From the perspective of controlling threats to the internal validity of research findings, using the difference between the pretest-posttest mean changes of the two groups is preferable to ignoring the pretest means (cf., e.g., Shadish et al., 2002). Therefore, conceptually, it is preferable to use the difference between the pretest-posttest mean changes in the numerator rather than the posttest mean difference. Based on this premise, the parameter we wish to estimate is this difference divided by a standardizer. If the parameter of interest is that difference, then it no longer makes sense to use SMDEP/EP as the "gold standard." The authors' conclusion that SMDCS/BL or SMDCS/EP overestimates the true population effect is based on treating SMDEP/EP as the ideal effect size, which is an unwarranted assumption. In other meta-analyses (outside the study of depression), it is possible that using SMDCS/BL or SMDCS/EP could result in lower estimates than SMDEP/EP. This is an empirical question that may vary across fields. However, the important point is that conclusions about the performance of SMDCS/BL or SMDCS/EP cannot be drawn based on SMDEP/EP as the "gold standard" if the meta-analyst is interested in estimating a parameter different from that estimated by SMDEP/EP. In my experience replicating meta-analyses, I have encountered examples where using SMDEP/EP yielded a highly significant pooled effect favoring the treatment, while SMDCS/BL produced a mean effect of zero."

RESPONSE:
If we understand correctly, the reviewer argues that effect sizes based on change scores are preferable to endpoint scores because they are a generally appropriate method to control for baseline imbalances. We have to respectfully disagree with the reviewer on this point. Change scores will only adequately adjust for baseline in one

specific scenario: when the relationship between baseline and post-test β is linear, β is exactly 1, and when this is the case in both groups. This will rarely be the case [1]. This point is also described in the primer section of our article (Page 7), with equation (2) showing it algebraically (see also [1,2], for similar presentations). In the primer section, we also state that endpoint scores implicitly provide adjustment for baseline in the same way if we instead just assume β to be exactly 0.

The fact that change scores do not provide an acceptable adjustment for baseline scores is frequently mentioned in the statistical literature. We quote from a few authoritative sources:

- “The change score as the outcome measure does not address the problem of regression to the mean, nor does it take account of the baseline imbalance.” [3]

- “If the assignment to treatment groups of the individual trials at baseline has not been truly random and the changes in the treatment groups are not due to the intervention alone, using mean change scores will not account for factors such as regression to the mean that may explain findings.” [4]

- The Cochrane Handbook for Systematic Reviews of Interventions states that, although change from baseline is “sometimes used as a device to ‘correct’ for unlucky randomization, this practice is not recommended” [5].

- Further problematic assumptions of using change scores in intervention studies are discussed in a text by Frank Harrell [6].

In support of change scores, the reviewer cites Shadish et al. [7]. We have screened this book carefully, and want to apologize in advance in case we have simply missed the section the reviewer was referring to. Yet, we were not able to find a passage in this book suggesting that change scores are an adequate method to control for baseline imbalances. In contrast, the authors explicitly state that “here and elsewhere, we do not mean to imply that change scores would be desirable as measures of that improvement” in the context of randomized designs (chap. 8; page 261).

We want to emphasize that this does not mean that baseline imbalances, differential attrition, or other internal biases are not a problem in randomized trials. It only means that change scores are not a generally preferable method to address this issue than endpoints alone. In our assessment, a good (and commonly recommended) way to control for internal biases in the mental health field is to apply established tools such as Cochrane’s RoB tool, so that sensitivity analyses in studies with low risk of bias only can be performed. This applies to both change score and endpoint SMDs, both of which will provide unbiased effect estimates if the studies themselves are unbiased. Trowman et al. [4] suggest using meta-regression as another option if very strong baseline imbalances are found in a meta-analyses, but also explicitly state that using change scores is not appropriate for this purpose.

The reviewer states that “[t]he authors' conclusion that SMDCS/BL or SMDCS/EP overestimates the true population effect is based on treating SMDEP/EP as the ideal effect size, which is an unwarranted assumption”. We believe this misrepresents the goals of our paper. In the “Aims of the current study” section (Page 11-12), we state

that we aim to examine how strongly SMD calculation methods can diverge from each other, using a real-world meta-analytic database at basis; and that we want to inspect predictors of such divergences. We fully agree that it does not make sense to see one of these calculation methods as the "gold standard", or to say that one method generally "overestimates" the true effect, while the other does not. However, such claims were also never made in the paper. We have now revised our "Aims" section to make this point even clearer.

As described in our discussion (Page 20ff.), our preference for SMD(EP/EP) is based on pragmatic reasons: we believe SMD(EP/EP) is the most useful variant in our context because it is clearly defined (endpoint means only, endpoint SDs only); it can be computed from the endpoint scores alone; and because it does not require the derivation/guessing of quantities that are typically not reported (e.g., pre-post correlations). We believe this is important to avoid two analysts coming up with two strikingly different values of the "standardized" treatment effect, even when the data are the same. SMDs from different meta-analyses are often directly compared in the literature, and this could lead to misinterpretations if SMD calculation methods are used inconsistently in a field. For meta-analyses of psychological treatment trials, we would argue that consistent use of SMD(EP/EP) minimizes this risk.

However, we do agree that some of these points could be emphasized even more directly in our paper. In the manuscript, we now describe explicitly that one should not see SMD(EP/EP) as a "gold standard" from a statistical perspective; and that our recommendations may not generalize to other fields, including non-randomized studies. We also made our recommendation in the abstract less strong, suggesting that researchers "could" prioritize endpoint SMDs in meta-analyses of clinical trials.

References:
[1] McKenzie, J. E., Herbison, G. P., & Deeks, J. J. (2016). Impact of analysing continuous outcomes using final values, change scores and analysis of covariance on the performance of meta-analytic methods: a simulation study. *Research synthesis methods*, *7*(4), 371-386.
[2] Laird, N. (1983). Further comparative analyses of pretest-posttest research designs. *The American Statistician*, *37*(4a), 329-330.
[3] Clifton, L., & Clifton, D. A. (2019). The correlation between baseline score and post-intervention score, and its implications for statistical analysis. *Trials*, *20*, 1-6.
[4] Trowman, R., Dumville, J. C., Torgerson, D. J., & Cranny, G. (2007). The impact of trial baseline imbalances should be considered in systematic reviews: a methodological case study. *Journal of clinical epidemiology*, *60*(12), 1229-1233.
[5] Higgins JPT, Thomas J, Chandler J, Cumpston M, Li T, Page MJ, Welch VA (editors). *Cochrane Handbook for Systematic Reviews of Interventions (chap. 10)* version 6.5 (updated August 2024). Cochrane, 2024. https://training.cochrane.org/handbook/current/chapter-10#section-10-5-2.
[6] Harrell, F. (2017). Statistical Errors in the Medical Literature. www.fharrell.com/post/errmed/#change.
[7] Shadish, W. R., Cook, T. D., & Campbell, D. T. (2002). *Experimental and quasi-experimental designs for generalized causal inference.* Houghton, Mifflin and Company.

CHANGES TO THE MANUSCRIPT:
Page 11, Line 243: "In this study, we therefore aim to systematically investigate the impact of different SMD calculation methods on the estimated meta-analytic effect of psychotherapy for depression. Focusing on aggregate-data information reported in the publications, we will examine different approaches to obtain the mean difference

between groups (endpoint scores versus change scores), as well as the standardizing denominator (pooled pre-test, change score, or endpoint SD), and examine how strongly these SMD variants can diverge from each other. We will also assess the influence of pre-post correlations (ranging from low to high) that meta-analysts may be willing to assume when calculating the SMDs."

Page 21, Line 495: "To enhance transparency, researchers may also provide a pre-specification of the SMD calculation method they plan to employ, to prevent selective reporting of the one variant yielding the highest effect size. In this context, it is important to re-emphasize that none of the SMD calculation methods we examined here is inherently "wrong" or biased. Researchers may still select a different variant than $SMD_{EP/EP}$ for their analysis; but this should be clearly described, since it could limit the comparability of the effect size. We also want to underline that our recommendations in this paper are purely pragmatic, and may not translate to every context in mental health research. Examples include quasi-experimental designs, or sub-fields in which pre-post correlations are typically reported."

Page 3, Line 62 (Abstract): "Researchers could prioritize endpoint SMDs of randomized trials, and should consider standardization using population-level estimates to improve the comparability of meta-analytic effects in the field."

COMMENT 4:
"The use of SMDCS/BL or SMDCS/EP might be limited if the results varied drastically depending on the imputed correlation. However, the results presented in this study do not suggest this. In Table 1, we can see that the pooled effects of SMDCS/BL for correlations of 0.2, 0.4, 0.6, and 0.8 were 0.92, 0.92, 0.93, and 0.93, respectively. For SMDCS/EP, the mean effects were 0.82, 0.83, 0.83, and 0.83. The results presented in the supplementary tables are consistent with this. That is, the imputed correlation had a negligible effect on the pooled effect. Therefore, SMDCS/BL and SMDCS/EP are perfectly applicable estimators, even if the correlation needs to be imputed. Furthermore, conceptually, they are preferable to SMDEP/EP as they account for pretest scores."

RESPONSE:
We agree with the reviewer's interpretation that change score SMDs do not depend strongly on the assumed pre-post correlations if alternative standardizers (viz., baseline or endpoint SDs) are used. However, it should be noted that this approach deviates from the definition of change score SMDs in some widely used texts, such as in Cohen [1], or the Cochrane Handbook [2], both of which present versions of SMD(CS/CS). In contrast to SMD(EP/EP), there is no consensus on the most suitable standardizer for change score SMDs. This is exactly the problem we see with change score SMDs, acknowledging that effect estimates from different meta-analyses are often compared with each other.

It seems we are also in agreement with the reviewer that standardizing denominators should best not depend on (imputed) correlations. This is the case "by default" with SMD(EP/EP), since we simply standardize differences in endpoint scores by their pooled SD. The remaining disagreement is about the use of change scores in the numerator. Here, the reviewer re-emphasizes the claim that change scores are the

preferable method because they adequately control for baseline imbalances ("account for pretest scores"). This point has already been addressed in detail in our response to comment 3 above, where we explain our disagreement, and recapitulate our pragmatic arguments in favor of SMD(EP/EP).

As mentioned in our response to comment 3, we have now revised our manuscript to make our rationale clearer.

References:
[1] Cohen J. "Qualifying" Dependent Variables (chap. 11.4; eq. 11.4.5; p. 539). Statistical Power Analysis for the Behavioral Sciences 2nd ed Lawrence Erlbaum Associates; 1983.
[2] Higgins J, Thomas J. Imputing standard deviations for changes from baseline (chap. 6.5.2.8). Cochrane Handbook for Systematic Reviews of Interventions 6.4. 2023. Available from: https://training.cochrane.org/handbook/current/chapter-06#section-6-5-2-8

CHANGES TO THE MANUSCRIPT:
Page 21, Line 495: "To enhance transparency, researchers may also provide a pre-specification of the SMD calculation method they plan to employ, to prevent selective reporting of the one variant yielding the highest effect size. In this context, it is important to re-emphasize that none of the SMD calculation methods we examined here is inherently "wrong" or biased. Researchers may still select a different variant than $SMD_{EP/EP}$ for their analysis; but this should be clearly described, since it could limit the comparability of the effect size. We also want to underline that our recommendations in this paper are purely pragmatic, and may not translate to every context in mental health research. Examples include quasi-experimental designs, or sub-fields in which pre-post correlations are typically reported."

Page 3, Line 62 (Abstract): "Researchers could prioritize endpoint SMDs of randomized trials, and should consider standardization using population-level estimates to improve the comparability of meta-analytic effects in the field."

COMMENT 5:
"If the meta-analyst decides to use SMDCS/BL or SMDCS/EP, they can approximate a correlation by deriving it from studies in the meta-analysis that do report this correlation, averaging it, and imputing it to the rest of the studies that do not report it. This strategy would allow for a reasonably accurate approximation of the true pretest-posttest correlation. Note that some primary studies may not report the pretest-posttest correlation but do report descriptive statistics from which it can be calculated. For instance, if a study reports SDBL, SDEP, and SDCS, the correlation can be computed. If the study reports SDBL, SDEP, and the t-test comparing pretest and posttest means, the correlation can also be calculated. The authors should clarify these strategies to impute correlations as close as possible to the true parametric correlation."

RESPONSE:
We agree with the reviewer that this is very helpful information. In the manuscript, we now mention these methods as an approach to approximate the correlations if change score SMDs are chosen.

CHANGES TO THE MANUSCRIPT:
Page 8, Line 173: "A practical problem with (9) above is that such trial-specific correlation coefficients are rarely reported; the value of SMD_{CS} obtained using this method will therefore heavily depend on the value imputed for r_a. In meta-analyses, r_a values can be imputed using representative values from the literature, or sourced from other studies in the meta-analysis that provide empirical estimates. In some cases, r_a may also be approximated from other reported summary statistics [24]."

COMMENT 6:
"The decision of which effect size index to use in a meta-analysis must be based on the parameter the meta-analyst wishes to estimate. If the meta-analyst does not wish to account for pretest means, he/she should use SMDEP/EP. If the meta-analyst wants to consider the pretest-posttest mean changes of the two groups, he/she can use SMDCS/BL, SMDCS/EP, or SMDCS/CS. Among these options, I recommend always using the difference between the pretest-posttest mean changes. Once this decision is made, the next step is to choose the standardizer (i.e., the denominator). Since SMDCS/CS is heavily influenced by the imputation of the correlation coefficient, the decision would be between SMDCS/BL and SMDCS/EP. If the meta-analyst is interested in estimating the parametric effect relative to participants' status after treatment, the estimator of choice should be SMDCS/EP. If the meta-analyst considers that SDBL might be a more homogeneous estimator of variability across studies (as SDBL is unaffected by the treatment effect), he/she may opt for SMDCS/BL. In summary. Based on the results obtained by the authors, my conclusions differ significantly. The authors' preference for SMDEP/EP is neither empirically supported (SMDCS/BL and SMDCS/EP perform well) nor conceptually justified (using the difference between the pretest-posttest mean changes is conceptually superior to relying solely on posttest means)."

RESPONSE:
We agree with the reviewer that none of the calculation methods we discussed are inherently "wrong" or biased. Indeed, analysts are free to use different SMD calculation methods, and we do not see SMD(EP/EP) as a "gold standard". We have now revised our manuscript to make this point even clearer.

We also seem to be in agreement with the reviewer that, on purely pragmatic grounds, consistent use of one SMD calculation method would be desirable, for example because researchers often compare SMD values from different meta-analyses. Furthermore, we agree with the reviewer that it would be preferable to choose calculation methods that cannot not yield heavily diverging values depending on the imputed pre-post correlation (i.e., methods that use endpoint or baseline SDs to standardize), since those correlations are often not reported, and have to be approximated.

Thus, as mentioned in our response to previous comments, the central disagreement seems to revolve around the utility of change scores as a method to control for baseline imbalances. This point was addressed in detail in our response to comment 3, in which we summarize methodological literature emphasizing that change scores, in fact, do not provide a suitable method to adjust for baseline scores, even though

this is often assumed. Once this argument is set aside, we believe it becomes clearer why we argue in favor of SMD(EP/EP), since it is unambiguously defined, easy to compute, and requires the least amount of reported data. Any type of SMD can be biased if a randomized trial itself is biased. Methods such as Cochrane's RoB tool are a suitable method to address this issue, and have been developed exactly for this purpose.

Nevertheless, we do agree that this recommendation is based on a purely practical reasoning, which may not hold in some contexts (e.g., research fields in which pre-post correlations are routinely applied; fields in which change scores, but not endpoints scores can routinely be extracted; quasi-experimental research). This caveat has been added to the manuscript, and we have also revised our recommendation in the abstract to make it sound less strong ("should" to "could").

CHANGES TO THE MANUSCRIPT:
Page 21, Line 493: "In our analysis, 16.7% to 27.3% of trials had to be removed because they did not report group means and standard deviations at both pre- and post-test. This indicates that, in general, outcome reporting in psychotherapy trials needs to be improved. To enhance transparency, researchers may also provide a pre-specification of the SMD calculation method they plan to employ to prevent selective reporting of the one variant yielding the highest effect size. In this context, it is important to re-emphasize that none of the SMD calculation methods we examined here is inherently "wrong" or biased. Researchers may still select a different variant than $SMD_{EP/EP}$ for their analysis; but this should be clearly described, since it could limit the comparability of the effect size. We also want to underline that our recommendations in this paper are purely pragmatic, and may not translate to every context in mental health research; for example quasi-experimental designs, or sub-fields in which pre-post correlations are typically reported."

Page 3, Line 62 (Abstract): "Researchers could prioritize endpoint SMDs of randomized trials, and consider standardization using population-level estimates to improve the comparability of meta-analytic effects in the field."

Table 2: "Note. These are pragmatic recommendations for aggregate data meta-analyses of randomized trials in mental health research. Some of the points mentioned above may not translate to other fields, for example quasi-experimental designs. Please also note these are not recommendations for primary analyses of randomized trials, in which individual participant data is typically available. Suitable guidance on the primary evaluation of clinical trials in mental health research is provided elsewhere [2]."

REVIEWER 2

COMMENT 1:
"This manuscript tackles a longstanding and important problem in meta-analytic statistics for psychotherapy research: how to handle standardised mean differences (SMDs) when different approaches to calculating them can yield widely divergent summary estimates. The authors provide a thorough examination of change-score versus endpoint-based calculations, illustrating the practical implications through a large dataset on depression psychotherapy. Given the extensive reliance on SMDs in mental health research (and psychology research more broadly), the paper is both timely and methodologically instructive. The manuscript is well-organised: it starts by clearly laying out theoretical relationships among the various versions of SMD, then demonstrates these relationships empirically using a rich database. I commend the authors' careful approach to documenting and justifying each analytical decision. The open-data and open-code also enhances reproducibility."

RESPONSE:
Dear Dr. Noetel,
we thank you for this positive evaluation and the helpful feedback, which has allowed us to further improve the quality of our manuscript. A point-by-point response to your comments can be found below.

COMMENT 2:
"The finding that psychotherapy vs. control effects varied from 0.65 to 1.24, depending on the SMD calculation, is a striking result. This convincingly demonstrates to readers that "standardisation" is far from consistent. The manuscript rightly cautions against naïve comparisons of meta-analytic results that use different SMD methods. The authors rightly acknowledge several limitations—chief among them the reliance on aggregate data. It is not obvious to me why the study was not conducted using a public IPD dataset or just simulated data. From a readers perspective doing so would have meant the authors could claim the effect sizes not just vary wildly, but that some effect sizes are *wrong* by X. By relying on aggregate data, it seems the authors are deprived of 'ground truth.' Using IPD or simulated data would mean the authors could compare the meta-analytics estimates against that ground truth, showing that their recommended methods are both pragmatic (e.g., they don't need each study to report a pre-post correlation coefficient) and accurate (i.e., the estimate closely reflects the true value)."

RESPONSE:
We thank the reviewer for mentioning this point, which has also been picked up by the editor (see comment 1). We agree with the editor (and reviewer, we presume) that it may be out of scope to add an entire "full-scale" simulation study to this paper; particularly since several already exist on this topic (e.g. [1,2,3]). However, we do agree that a small-scale, "visual" simulation of the relationships we describe in the primer section is very helpful. This may help some readers to get a more intuitive understanding of our descriptions; and it also allows to gauge how different SMD variants perform if the true effect is known.

In S1 in the supplement, we now describe in detail the setup and results of a "toy" simulation study we conducted, assuming realistic parameter values for psychological

treatment trials. This simulation is also referenced in the manuscript itself. The new section includes a visual summary of our simulation, similar to the summary plot of our empirical investigation in Figure 1 in the main manuscript. This visualization may help readers to build a better understanding about the expected divergences between SMD calculation method, presuming several simplifying assumptions are made. The simulated results added to S1 also closely mirror the findings we obtained in the empirical investigation; and they align with our recommendation against SMD calculation methods that rely heavily on imputed pre-post correlations; just as predicted by the formulas described in our primer section.

Lastly, we also wanted to briefly explain why we chose to conduct an "empirical investigation" instead of conducting a simulation study (only). For this study, we were not primarily interested in the bias, coverage etc. of different SMD calculation methods. Instead, we wanted to assess how much meta-analytic effects of psychological treatment can realistically diverge depending on the chosen SMD calculation method, using the same dataset as basis. In a randomized trial, both change score and endpoint mean differences provide an asymptotically unbiased estimator of the average treatment effect; but this numerical value may start to diverge strongly as soon as effects are "standardized" using different denominators, with several plausible options being available. This is problematic from a pragmatic standpoint, because SMD values are often directly compared to each other to assess the effect of a treatment. We now tried to make this rationale even clearer throughout the manuscript.

References
[1] Gnambs, T., & Schroeders, U. (2024). Accuracy and precision of fixed and random effects in meta-analyses of randomized control trials for continuous outcomes. *Research synthesis methods*, *15*(1), 86-106.
[2] Hedges, L. V., Tipton, E., Zejnullahi, R., & Diaz, K. G. (2023). Effect sizes in ANCOVA and difference-in-differences designs. *British Journal of Mathematical and Statistical Psychology*, *76*(2), 259-282.
[3] McKenzie, J. E., Herbison, G. P., & Deeks, J. J. (2016). Impact of analysing continuous outcomes using final values, change scores and analysis of covariance on the performance of meta-analytic methods: a simulation study. *Research synthesis methods*, *7*(4), 371-386.

CHANGES TO THE MANUSCRIPT:
Page 10, Line 216: "Depending on what approaches are used, this may heavily limit the comparability of effect sizes within and across meta-analyses. Researchers could seriously over- or underestimate the efficacy of a treatment if SMD estimates are compared to other trials or meta-analyses using a different standardization method, or if wrong assumptions about the pre-post correlation are made. Results of our "toy" simulation shown in the supplement (S1) further illustrate this issue."

Page 19, Line 438: "Our results co-align with statistical theory, which predicts divergences between SMD estimates depending on the denominator used to "standardize" the effect (see, e.g., results of our simulation in supplement S1, which closely mirror our empirical findings)."

S1 added to the supplement (2 pages), showing the methodology and results of a simulation study assuming plausible true parameter values for psychological treatments.

Page 21, Line 494: “This indicates that, in general, outcome reporting in psychotherapy trials needs to be improved. To enhance transparency, researchers may also provide a pre-specification of the SMD calculation method they plan to employ, to prevent selective reporting of the one variant yielding the highest effect size. In this context, it is important to re-emphasize that none of the SMD calculation methods we examined here is inherently “wrong” or biased. Researchers may still select a different variant than $SMD_{EP/EP}$ for their analysis; but this should be clearly described, since it could limit the comparability of the effect size.”

COMMENT 3:
“The discussion section appropriately highlights the challenge of choosing an SMD. The authors ultimately suggest prioritising endpoint-based SMDs (SMDEP/EP) in meta-analyses and, when possible, using a common reference SD if outcomes are measured on the same scale. They also mention the possibility that future core outcome sets or standardised measurement approaches might do away with many of these complexities. In the meantime, I would encourage the authors to consider offering a pragmatic guideline or flowchart for researchers designing or synthesising new trials (a la https://bmcmedresmethodol.biomedcentral.com/articles/10.1186/s12874-017-0442-1). Such recommendations would be invaluable for future meta-analysts.”

RESPONSE:
We agree that this is a very helpful addition. While we were not able to create a visual flowchart as in the referenced paper, Table 2 now provides a concise textual summary of our practical recommendations, along with their explanations. We believe this new table will help readers distill the main “take-home” messages and implications of our paper.

CHANGES TO THE MANUSCRIPT:
Added Table 2, showing a summary of practical recommendations and their potential limitations.

Page 20, Line 468: “Table 2 presents practical recommendations for calculating SMDs from aggregate data from mental health trials.”

COMMENT 4:
“Some minor issues could likely be picked up in copy-editing and are of less substantive concern. For example, I assume IPD refers to 'individual patient data' but many psychotherapy researchers might not know this acronym; likewise clarify beta in equation 2.”

RESPONSE:
Many thanks to the reviewer for pointing this out. We have adapted the manuscript accordingly.

CHANGES TO THE MANUSCRIPT:
Page 6, Line 118: “Covariate adjustment typically requires that individual participant data (IPD) is available for the trial.”

Page 20, Line 464: “A recent investigation using IPD meta-analysis confirmed this [50], and further research may be helpful to corroborate this finding.” (IPD already introduced, IPD-MA not).

Page 6, Line 128: “This equation offers two insights. Firstly, it emphasizes that using change scores as the outcome will yield identical results to a standard ANCOVA if baseline scores are additionally controlled for. Secondly, it shows that a “crude” analysis of change scores without baseline adjustment will only be identical to ANCOVA when the slope between pre-test and post-test scores β is exactly one (since $(\beta - 1)X_a$ will only drop out of the equation when $\beta = 1$).”

COMMENT 5:
“Otherwise, this paper systematically quantifies the discrepancies in meta-analytic effect sizes that stem from different SMD calculation methods. It is of considerable importance for any researchers synthesising trial evidence on psychological interventions with heterogenous outcomes.”

RESPONSE:
We thank the reviewer once again for this positive evaluation.
